# Human Immunodeficiency Virus–Associated Proteomic Signature of Myocardial Fibrosis and Incident Heart Failure

**DOI:** 10.1093/infdis/jiag013

**Published:** 2026-04-29

**Authors:** Tess E. Peterson, Virginia S. Hahn, Ruin Moaddel, Min Zhu, Jinshui Fan, Supriyo De, Sabina A. Haberlen, Frank J. Palella, Michael Plankey, Joel S. Bader, Joao A. C. Lima, Robert E. Gerszten, Jerome I. Rotter, Gregory D. Kirk, Damani A. Piggott, Luigi Ferrucci, Joseph B. Margolick, Todd T. Brown, Wendy S. Post, Katherine C. Wu

**Affiliations:** 1 Division of Epidemiology and Community Health, University of Minnesota School of Public Health, Minneapolis, Minnesota, USA; 2 Division of Cardiology, Department of Medicine, Johns Hopkins University, Baltimore, Maryland, USA; 3 Biomedical Research Center, National Institute on Aging, National Institutes of Health, Baltimore, Maryland, USA; 4 Department of Epidemiology, Johns Hopkins Bloomberg School of Public Health, Baltimore, Maryland, USA; 5 Department of Internal Medicine, Northwestern University Feinberg School of Medicine, Chicago, Illinois, USA; 6 Division of General Internal Medicine, Department of Medicine, Georgetown University, Washington, District of Columbia, USA; 7 Department of Biomedical Engineering, Whiting School of Engineering, Johns Hopkins University, Baltimore, Maryland, USA; 8 Division of Cardiovascular Medicine, Beth Israel Deaconess Medical Center, Boston, Massachusetts, USA; 9 Institute for Translational Genomics and Population Sciences, Department of Pediatrics, The Lundquist Institute for Biomedical Innovation at Harbor-UCLA Medical Center, Torrance, California, USA; 10 Division of Infectious Diseases, Department of Medicine, Johns Hopkins University, Baltimore, Maryland, USA; 11 Department of Molecular Microbiology and Immunology, Johns Hopkins Bloomberg School of Public Health, Baltimore, Maryland, USA; 12 Division of Endocrinology, Diabetes, and Metabolism, Department of Medicine, Johns Hopkins University, Baltimore, Maryland, USA

**Keywords:** HIV, myocardial fibrosis, heart failure, inflammation, proteomics

## Abstract

**Background.:**

People with human immunodeficiency virus (HIV) (PWH) are at higher risk of myocardial fibrosis and subsequent heart failure (HF) compared to people without HIV (PWOH). Mechanisms underlying this risk and its specificity to PWH are unclear.

**Methods.:**

We measured 2594 proteins in plasma obtained concurrently with cardiovascular magnetic resonance imaging among 342 PWH and PWOH. We estimated associations with HIV serostatus and myocardial fibrosis (elevated extracellular volume fraction [ECV] ≥30% among women, ≥28% among men) using multivariable regression. Among an independent community-based cohort, we estimated associations between the identified signature and time to incident HF.

**Results.:**

Mean age of participants was 55 (standard deviation [SD], 6) years, 25% were female, 61% were PWH (88% on antiretroviral therapy, 74% with undetectable HIV RNA), and 52% had elevated ECV. We identified 39 proteins and 1 cluster of 42 proteins that were higher among PWH versus PWOH and positively associated with elevated ECV, independent of risk factors (false discovery rate <0.05). Among an independent cohort of 3223 PWOH (mean age, 68 [SD, 9] years; 52% female; 118 incident HF cases over a mean of 9.8 [SD, 1.4] years), we found that this protein cluster and 34 of 39 individual proteins were associated with time to incident HF. This signature was statistically enriched for T-cell activation, tumor necrosis factor signaling, ephrin signaling, and tissue maintenance and repair.

**Conclusions.:**

We identified an HIV-related proteomic signature associated with myocardial fibrosis regardless of HIV serostatus and that predicted incident HF among the general population. Our results identify several novel associations related to specific immune processes that may contribute to risk of myocardial fibrosis and subsequent HF among both PWH and PWOH.

Persons with human immunodeficiency virus (HIV-1) (PWH), even when virologically suppressed with antiretroviral therapy (ART), are at higher risk of heart failure (HF) [1] and the subclinical cardiac abnormalities that portend it, compared to persons without HIV (PWOH) [2]. This includes risk for diffuse interstitial myocardial fibro-inflammation (MF), which has emerged as a key mediator underlying both HIV-associated [2, 3] and aging-related cardiac dysfunction [4, 5]. MF is characterized by excess deposition of highly cross-linked type I collagen fibers and other extracellular matrix components in the interstitial and perivascular space. This accumulation distorts myocardial architecture and physiology, impairing function and contributing to development of clinical HF. We previously reported that PWH have higher diffuse MF compared to PWOH [6], supported by other studies spanning world regions [7].

Mechanisms contributing to HIV-associated MF and HF are hypothesized to be multifactorial, largely mediated by chronic inflammation and immune activation that persist despite ART-induced viral suppression [2]. However, therapeutic strategies to decrease this excess risk largely remain elusive. It also remains unclear whether mechanisms underlying this risk differ by HIV serostatus, considering that HIV-related immune dysfunction resembles aging-related immune activation [2], which is in turn a central pathogenic feature of MF and HF within the general population [8, 9]. Since preventive and therapeutic strategies for HF among PWH are currently guided by data from PWOH, addressing this knowledge gap could have significant clinical implications.

We performed high-throughput proteomic profiling on plasma obtained concurrently with cardiovascular magnetic resonance imaging (CMR) among largely ART-treated PWH and sociodemographically similar PWOH in the Subclinical Myocardial Abnormalities in HIV (SMASH) study [6]. We previously identified plasma proteome features that differed by HIV serostatus and were associated with left atrial remodeling in SMASH [10]. Guided by the same framework, we herein assess which HIV-associated proteome features associate with MF. We then externally evaluate this proteomic signature for associations with MF and time to incident adjudicated clinical HF among an independent community-based cohort of PWOH in the Multi-Ethnic Study of Atherosclerosis (MESA). Our objective was to identify novel HIV-associated markers of MF and to test the hypotheses that such markers are not HIV-specific and moreover predict incident HF in the general population.

## METHODS

### SMASH Study Population

SMASH participants were recruited from the Multicenter AIDS Cohort Study (MACS) [11], the Women’s Interagency HIV Study (WIHS) [12], and the AIDS Linked to the IntraVenous Experience (ALIVE) study [13] to undergo CMR with blood collection using a centralized protocol (2015–2018) [6]. SMASH enrolled participants aged 40–70 years from Chicago, Illinois and Baltimore/Washington, DC. Exclusion criteria included estimated glomerular filtration rate (eGFR) <45 mL/minute/1.73 m^2^, body weight >350 lb (159 kg), contrast allergy, and CMR contraindications. Proteomics was performed on the participant subset with complete CMR (Supplementary Figure 1A). Additional cohort details are shown in the Supplementary Methods. This study complies with the Declaration of Helsinki and was approved by the institutional review boards of Johns Hopkins University, Georgetown University, and Northwestern University. All participants provided written informed consent.

### SMASH Cardiovascular Magnetic Resonance

CMR was performed at Johns Hopkins Hospital or Northwestern Memorial Hospital on 1.5T Siemens Avanto or Aera scanners, using a standardized protocol that included gadolinium enhancement [6]. In brief, T1 mapping with a modified Look–Locker inversion (MOLLI) recovery sequence was performed before and after intravenous administration of 0.2 mmol/kg of gadobutrol (Gadavist). We used MRmap v1.2 (Charite University) to post-process MOLLI images. After contouring left ventricular (LV) endocardium and epicardium in basal, mid, and apical slices, each slice was segmented into 6 sections. A 3-parameter curve fit of MOLLI images was performed with automatic calculation of per-sector T1 values. The partition coefficient was determined by the slope of the linear relationship of 1/T1_myocardium_ versus 1/T1_blood_ pre- and postcontrast. Extracellular volume (ECV, %) was calculated as ECV = 100 × partition coefficient × [1 – hematocrit]) [14]. We used sex-specific ECV thresholds ≥1 standard deviation (SD) above the mean to define elevated ECV: ≥30% for women and ≥28% for men [15].

### SMASH Proteomics

We performed proteomics on ethylenediaminetetraacetic acid plasma stored at −80°C, using the Olink Explore 3072 platform at the National Institute on Aging’s Laboratory of Clinical Investigation [10]. Olink technology has been previously described [16]; details and quality control procedures are shown in the Supplementary Methods. Values are log_2_-transformed relative levels, which were winsorized to 5 SD to minimize the effect of outliers and standardized for comparability. In addition to individual protein assessment, we also derived clusters of correlated proteins using weighted gene co-expression network analysis (WGCNA) and the R ‘WGCNA’ package (Supplementary Methods), as reported previously [10].

### Statistical Modeling

**Figure 1.**
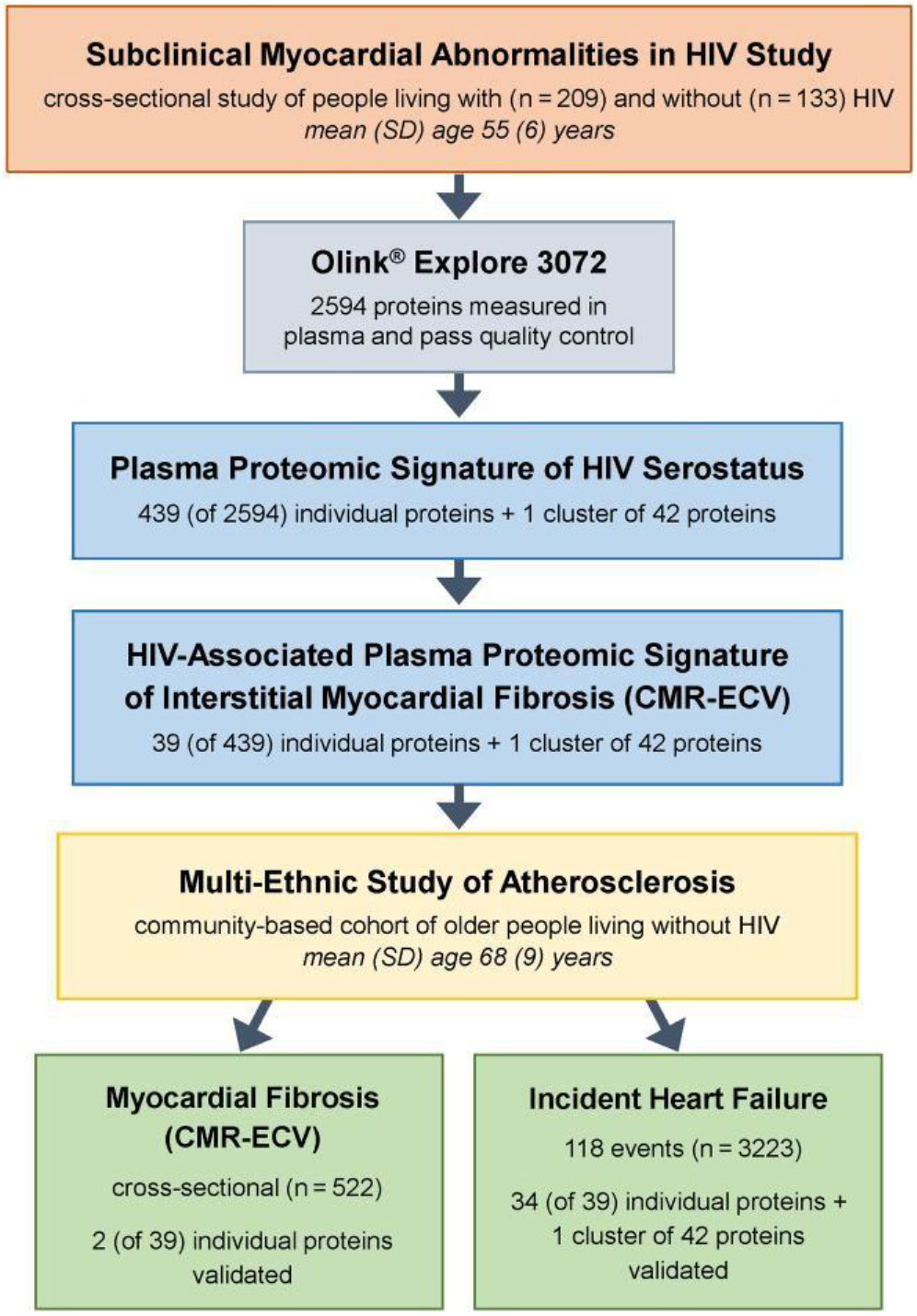
Study overview and analytic flow. Abbreviations: CMR-ECV, myocardial extracellular volume fraction ascertained by cardiovascular magnetic resonance imaging; HIV, human immunodeficiency virus; SD, standard deviation.

The analytic flow is depicted in Figure 1. Prevalence ratios (PRs) for elevated ECV were estimated per SD increment in previously identified HIV-associated proteome features (individual proteins or protein cluster) using modified Poisson regression, adjusting for age, sex, race, ethnicity, education, hazardous alcohol use, other substance use over the prior 5 years (pack-years of smoking, stimulant use, opioid use), HIV serostatus, hepatitis C virus infection (HCV), body mass index (BMI), systolic blood pressure (SBP), hypertension medication use, dyslipidemia, diabetes, and eGFR. We did not adjust for study site due to collinearity with sex. We then evaluated differences in protein–ECV associations by HIV serostatus using multiplicative interactions. Inference was made using a Benjamini–Hochberg false discovery rate (FDR) threshold of <0.05. Proteins of interest were defined as features significantly associated with positive HIV serostatus and elevated ECV with concordant directionality, potentially reflecting augmented pathobiology among PWH.

### Functional Annotation Analysis

Overrepresentation analysis was performed using Gene Ontology: Biological Process (GO:BP) annotations (R ‘clusterProfiler’ package) [17]. The applied background was measured proteins that passed quality control in SMASH.

### External Validation Among Older Persons Without HIV in MESA

External validation was performed in MESA, a prospective community-based cohort initiated in 2000 to study the characteristics and progression of subclinical cardiovascular disease (CVD) among a diverse population [18]. We used data from exam 5 (2010–2012) when participants were aged 53–94 years and when the same proteomics platform and CMR protocol [4] as those in SMASH were concurrently measured. See the Supplementary Methods for details on the MESA study population, data acquisition, and ascertainment/adjudication of clinical events.

We estimated cross-sectional associations between proteins of interest and elevated ECV using the same modeling approach as in SMASH, adjusting for site, age, sex, race, ethnicity, education, smoking, heavy alcohol use, BMI, SBP, hypertension therapy, dyslipidemia, diabetes, and eGFR. We then assessed associations with time to incident clinical HF, overall and by subtype with preserved (HFpEF) or reduced (HFrEF) LV ejection fraction (LVEF ≥50% vs <40%, respectively). Participants with prevalent HF at the start of follow-up were excluded. Time at risk was calculated as the time between MESA exam 5 date and an incident event, death, loss to follow-up, or end of follow-up, whichever occurred first. Hazard ratios (HRs) were estimated per SD increment in proteome feature using Cox regression, adjusting for the same covariates as cross-sectional analyses. Where appropriate for visualization, we secondarily estimated HRs across quartiles of plasma protein levels.

## RESULTS

### SMASH Participant Characteristics

The discovery analysis sample comprised 342 participants (Supplementary Figure 1), with mean age 55 (SD, 6) years, 25% female, 61% PWH, and with predominantly normal systolic function measured by LVEF (Table 1). The overall prevalence of elevated ECV was 52% and was higher among PWH compared to PWOH (57% vs 45%, respectively; *P* = .05 for difference, adjusting for demographics, substance use, and traditional risk factors).

### HIV-Associated Proteomic Signature of Myocardial Fibrosis in SMASH

**Figure 2.**
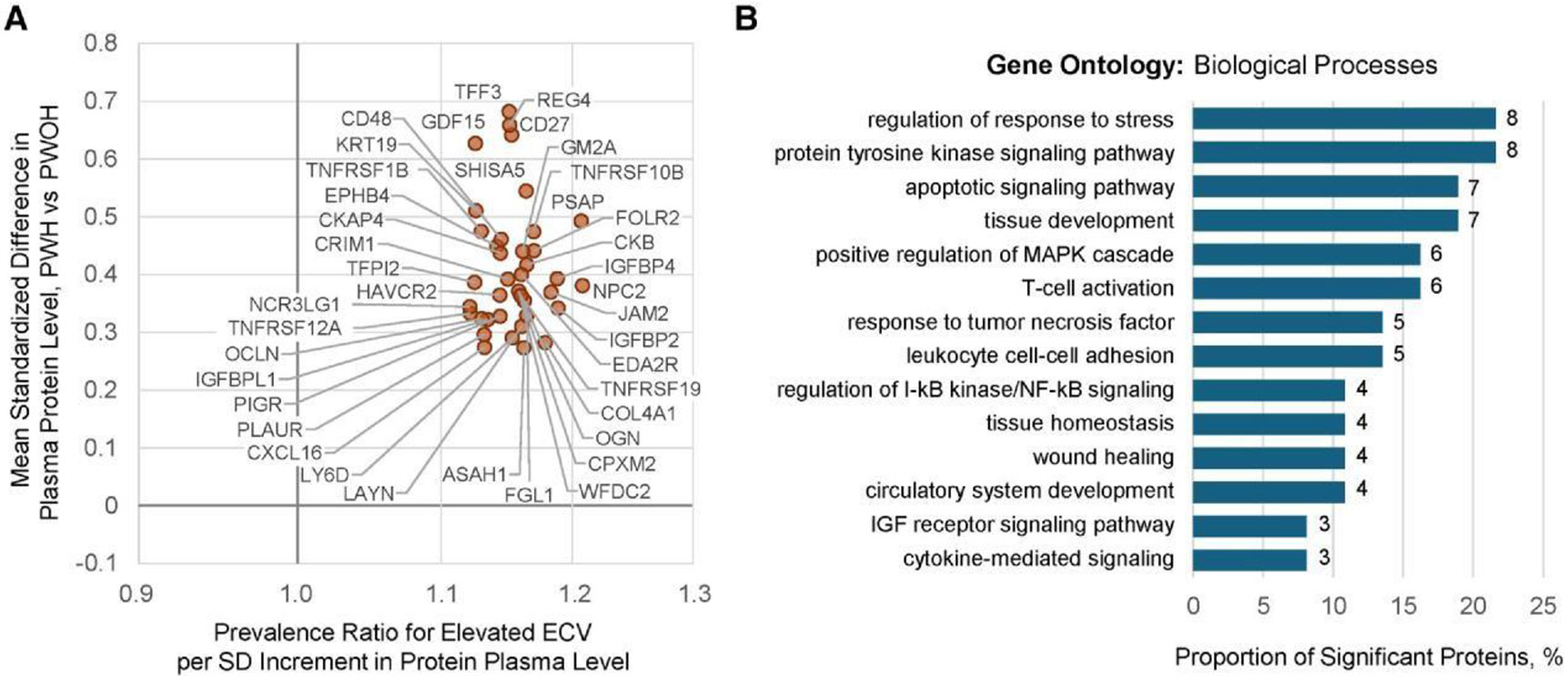
HIV-associated proteomic signature of myocardial fibrosis among SMASH study participants with and without HIV (n = 342). *A*, Beta-beta plot of (x-axis) prevalence ratio of elevated extracellular volume fraction (ECV ≥30% among women and ≥28% among men) per SD increment in individual plasma protein abundances, estimated using modified Poisson regression with robust variance vs (y-axis) mean differences in standardized protein abundances comparing PWH vs PWOH, estimated using linear regression with robust variance. Proteins depicted (n = 39) are restricted to those significantly associated with both parameters at an FDR <0.05. HIV point estimates (y-axis) were adjusted for age, sex, race, ethnicity, education, current hazardous alcohol use, pack-years of smoking in prior 5 years, stimulant use in prior 5 years, opioid use in prior 5 years, hepatitis C virus infection, and estimated glomerular filtration rate. ECV point estimates (x-axis) were adjusted for the same covariates, as well as body mass index, systolic blood pressure, anti-hypertensive medication, dyslipidemia, and diabetes. Complete results can be found in Supplementary Tables 1 and 5. *B*, Proportion of 37 proteins successfully mapped to select Gene Ontology: Biological Process annotations and associated with both positive HIV serostatus and elevated ECV at FDR <0.05 with concordant directionality. Number at each bar indicates signature protein count, noting some proteins may map to multiple depicted processes. No processes were significantly overrepresented by Fisher exact test following multiple testing correction. Depicted processes were manually curated, considering pathway redundancy, highest protein count, and lowest *P* value. Complete overrepresentation results, including proteins mapping to each process, can be found in Supplementary Table 7. Abbreviations: ECV, extracellular volume fraction; FDR, false discovery rate; IGF, insulin-like growth factor; MAPK, mitogen-activated protein kinase; PWH, people with human immunodeficiency virus; PWOH, people without human immunodeficiency virus; SD, standard deviation.

Of 2594 measured proteins, plasma levels of 439 individual proteins and 1 agnostically defined cluster of 42 proteins were each significantly associated with HIV serostatus (Supplementary Tables 1-4), reported previously [10]. Given that we are uniquely poised to evaluate HIV-related biology within SMASH, we herein evaluated which of these HIV-associated proteins were also associated with elevated ECV and whether those associations differed by HIV serostatus. Of 439 proteins, 39 were associated with elevated ECV with concordant directionality (all positively associated), independent of demographics, substance use, HIV serostatus, HCV, renal function, and traditional CVD risk factors (Figure 2A, Supplementary Table 5). In subgroup analyses, mean plasma levels of all 39 proteins remained higher among PWH with undetectable plasma HIV RNA (<50 copies/mL), compared to PWOH.

The top 5 strongest protein associations with elevated ECV included prosaposin (PSAP), NPC intracellular cholesterol transporter-2 (NPC2), insulin-like growth factor–binding protein-4 (IGFBP4), junctional adhesion molecule-2 (JAM2), and collagen type IV alpha-1 (COL4A1) (all FDR <0.02), with point estimates ranging from 1.18 to 1.21 times higher prevalence per SD increment. These 39 proteins exhibited pairwise correlations that ranged widely (Supplementary Figure 2, Supplementary Table 6).

Of these 39 proteins, 37 were mapped to GO:BP. While these 37 proteins were not statistically enriched for any processes following multiple testing correction, we observed patterns highlighting processes related to tissue maintenance and repair (apoptotic signaling pathway, regulation of the MAPK cascade, wound healing); regulation of immune processes (T-cell activation, leukocyte migration); and cytokine signaling (eg, tumor necrosis factor [TNF]) (Figure 2B, Supplementary Table 7).

Next, we determined what proportion of the multivariable association between HIV and ECV was accounted for by further adjustment for each of 39 proteins. Six proteins reduced the HIV–ECV association by >35%: CD27, folate receptor-2 (FOLR2), PSAP, regenerating family member-4 (REG4), shisa family member-5 (SHISA5), and trefoil factor-3 (TFF3) (Supplementary Table 8). We found no protein × HIV interactions (all FDR >0.90, Supplementary Table 9).

Given differences in reactive versus reparative fibrosis, we performed sensitivity analyses excluding 19 participants with prior myocardial infarction and/or ischemic scar pattern on CMR. All PR point estimates differed by <3% (Supplementary Table 10). We also evaluated associations between proteins of interest and T2 time in a small subset of participants with measured T2 (n = 81). We found none associated (Supplementary Table 11), suggesting there is not strong support that the identified proteomic signature reflects myocardial edema.

**Figure 3.**
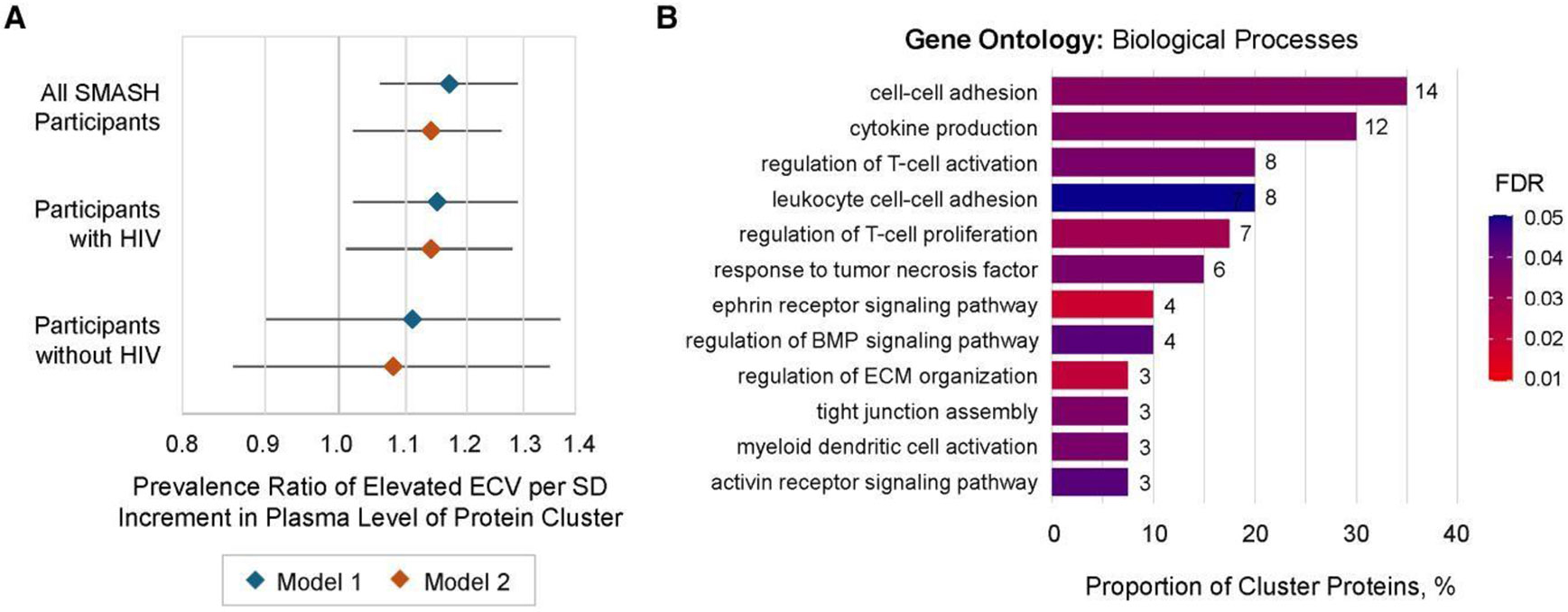
Relationship between an HIV-associated, agnostically defined cluster of plasma proteins and myocardial fibrosis among SMASH study participants with and without HIV (n = 342). *A*, Prevalence ratio of elevated extracellular volume fraction (ECV ≥30% among women and ≥28% among men) per SD increment in protein cluster plasma abundance among all participants and among strata of participants with and without HIV, estimated using modified Poisson regression with robust variance. Model 1 adjusts for age, sex, race, ethnicity, HIV, hepatitis C virus infection, and estimated glomerular filtration rate. Model 2 further adjusts for education, body mass index, systolic blood pressure, anti-hypertensive medication, dyslipidemia, diabetes, current hazardous alcohol use, pack-years of smoking in prior 5 years, stimulant use in prior 5 years, and opioid use in prior 5 years. *B*, Proportion of proteins comprising the protein cluster mapping to Gene Ontology: Biological Process annotations; 40 of 42 total proteins successfully mapped. Number at each bar indicates protein count, noting some proteins may map to multiple depicted processes. FDR for overrepresentation estimated using Fisher exact test. Proteins mapping to each annotation can be found in Supplementary Table 4. Abbreviations: BMP, bone morphogenetic protein; ECM, extracellular matrix; ECV, extracellular volume fraction; FDR, false discovery rate; HIV, human immunodeficiency virus; SD, standard deviation; SMASH, Subclinical Myocardial Abnormalities in HIV.

In addition to individual proteins, we also previously identified 1 agnostically defined cluster of 42 proteins that was associated with HIV serostatus (Supplementary Tables 3 and 4, Supplementary Figure 3) [10]. Here, we found that highe plasma level of this cluster was also associated with elevated ECV (adjusted PR [aPR], 1.13 per SD increment in plasma cluster [95% confidence interval {CI}, 1.02–1.25]; *P* = .02, Figure 3A). This association did not differ by HIV serostatus (interaction *P* = .91), and adjustment for this cluster reduced the multivariable HIV–ECV association by 27%. Of 42 proteins comprising this cluster, 40 were mapped to GO:BP and were statistically enriched for T-cell activation, TNF signaling, ephrin signaling, and extracellular matrix organization (Figure 3B). Moreover, 10 proteins within this cluster were also identified in individual protein analyses: cytoskeleton-associated protein-4 (CKAP4), ephrin-B4 (EPHB4), T-cell immunoglobulin and mucin domain-3 (TIM-3/HAVCR2), IGFBP4, JAM2, layilin (LAYN), SHISA5, TNFRSF1B, TNFRSF10B, and TNFRSF19.

### Associations Between Proteomic Signature and Clinical Variables in SMASH

Many proteins were associated with age (*P* < .05), including 24 of 39 proteins and the protein cluster of interest. Notably, few proteins were associated with traditional CVD risk factors—for example, obesity (6 of 39 proteins, *P* < .05), hypertension (14), diabetes (8), and dyslipidemia (6)—whereas many were associated with opioid use (32), current smoking (28), HCV (33), and lower CD4^+^ T-cell counts (36) (Supplementary Figure 4). Similarly, the protein cluster was only associated with substance use and HIV-related clinical factors—smoking, opioid use, HCV, detectable plasma HIV RNA, and lower current and nadir CD4^+^ T-cell counts.

### External Validation of Protein Signatures With Myocardial Fibrosis in MESA

A total of 522 MESA participants underwent gadolinium-enhanced CMR imaging with ECV fraction and plasma proteomics (Supplementary Figure 5), characteristics of whom are summarized in Supplementary Table 12. Participants had a mean age of 68 (SD, 9) years, 48% were female, and participants had predominantly normal LVEF.

We tested each feature of the proteomic signature we identified in SMASH for associations with elevated ECV in MESA. Among this external cohort of older PWOH, 2 of 39 individual plasma proteins were cross-sectionally associated with high ECV: polymeric immunoglobulin receptor (PIGR) and FOLR2 (Table 2, Supplementary Table 13). Plasma level of the protein cluster was not significantly associated with elevated ECV in MESA, though the estimate was imprecise (aPR, 1.21 [95% CI, .94–1.55]; *P* = .139).

### External Evaluation of Protein Signatures With Incident Clinical Heart Failure in MESA

Incident event analyses did not require CMR and could therefore be analyzed in a larger MESA subpopulation to maximize statistical power (Supplementary Figure 5). Distributions of participant characteristics were similar to cross-sectional analyses (Supplementary Table 12).

**Figure 4.**
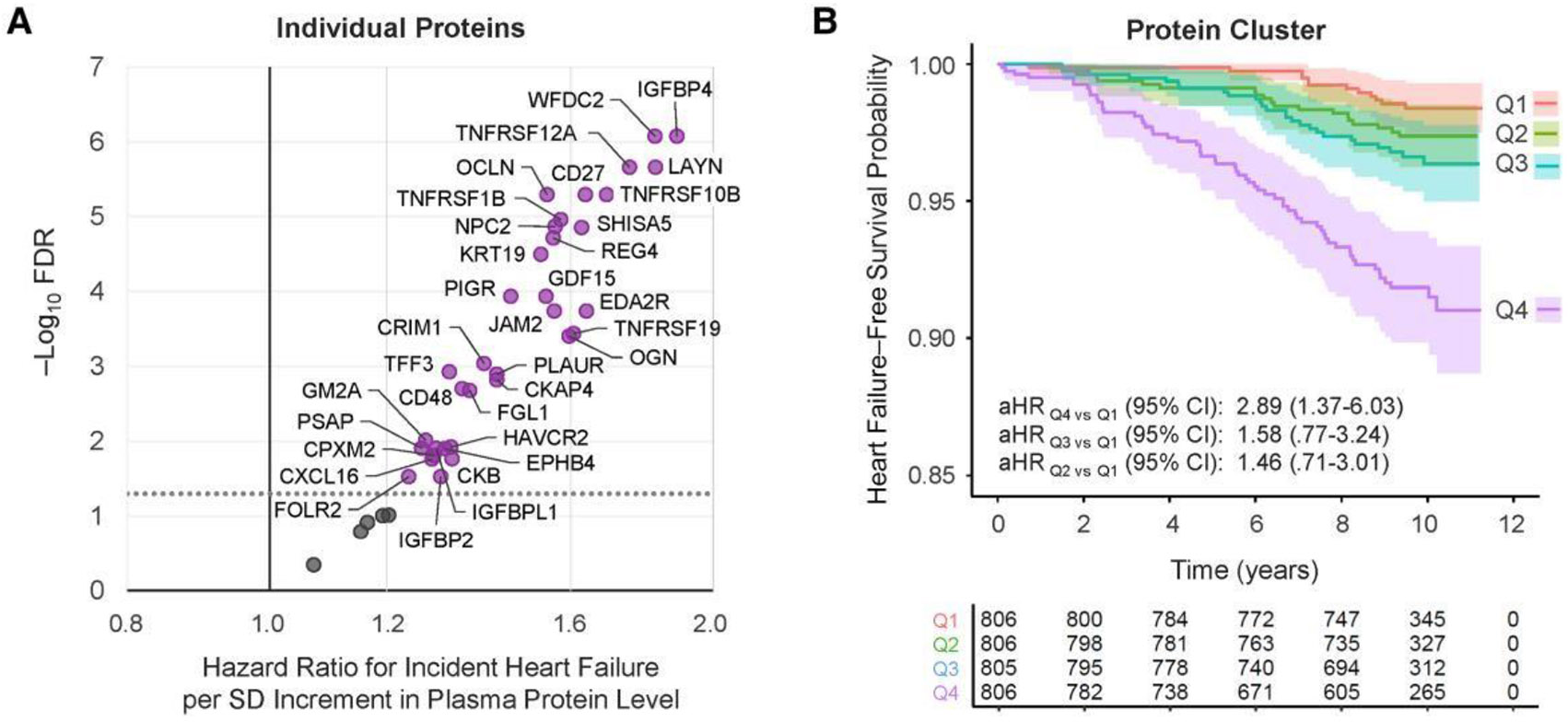
Association between identified HIV-associated proteomic signature of myocardial fibrosis and time to incident adjudicated clinical heart failure among people without HIV in MESA (n = 118 incident cases among 3223 participants). *A*, Hazard ratios for incident heart failure estimated per SD increment in plasma level of individual proteins, using Cox regression, adjusting for study site, age, sex, race, ethnicity, education, body mass index, systolic blood pressure, anti-hypertensive medication, dyslipidemia, diabetes, smoking, and estimated glomerular filtration rate. The dotted line indicates the threshold for statistical significance (FDR <0.05). Complete modeling results can be found in Supplementary Table 14. *B*, Kaplan-Meier curve displaying unadjusted survival by protein cluster quartile over time. The aHRs and 95% CIs for incident heart failure were estimated across quartiles (Q1 = ref) in plasma level of the protein cluster, using Cox regression adjusting for the same covariates described in (*A*). Abbreviations: aHR, adjusted hazard ratio; CI, confidence interval; FDR, false discovery rate; HIV, human immunodeficiency virus; MESA, Multi-Ethnic Study of Atherosclerosis; SD, standard deviation.

Among 3223 participants without prevalent HF at exam 5, there were 118 incident adjudicated HF events over a median follow-up of 9.8 (interquartile range, 1.0) years. Of 39 individual proteins, 34 were associated with time to incident HF following adjustment for covariates and multiple testing (Figure 4A, Supplementary Table 14). The strongest of these was IGFBP4 (adjusted HR [aHR], 1.89 per SD [95% CI, 1.51–2.38]; FDR = 8.43 × 10^−7^). Analyses by HF subtypes had lower statistical power but in many cases yielded higher HR point estimates for HFpEF versus HFrEF (Supplementary Table 15). Both proteins associated with high ECV in MESA were also associated with time to incident HF: PIGR (FDR = 1.04 × 10^−4^) and FOLR2 (FDR = 0.028), with similar point estimates by HF subtype for PIGR, and a higher point estimate comparing HFpEF to HFrEF for FOLR2 (Table 2). Plasma PIGR and FOLR2 were weakly correlated with each other in both SMASH (*r* = 0.34) and MESA (*r* = 0.27).

Hazard of HF was also greater with higher levels of the protein cluster (aHR, 1.71 per SD [95% CI, 1.40–2.10]; *P* = 2.29 × 10^−7^), with similar estimates for HFpEF (aHR, 1.80 [95% CI, 1.33–2.44]) and HFrEF (aHR, 1.56 [95% CI, 1.06–2.30]). In secondary analyses by cluster quartile, we observed 2.89 times higher hazard of HF (95% CI, 1.37–6.03; *P* = .005), comparing the highest to lowest quartile (Figure 4B). We previously reported the linear association with HF among a smaller MESA sample (n = 2273, 54 incident cases) with shorter follow-up duration (median 8.9 years), where inference was consistent [10]. Notably, all 10 proteins identified in both single-protein and cluster-based discovery analyses (listed above) were associated with incident HF.

### Associations Between Proteomic Signature and Clinical Variables in MESA

Every component of the protein signature predictive of clinical HF among MESA participants was positively associated with age (all *P* < 1 × 10^−6^, Supplementary Figure 6). Many were associated with hypertension (32 of 34 individual proteins and protein cluster), diabetes (32 proteins and cluster), and obesity (23 proteins and cluster), which remained associated when adjusted for eGFR. All aforementioned protein associations with ECV and incident HF were adjusted for these factors.

## DISCUSSION

In this discovery study building on our prior work, we identified an HIV-related plasma proteomic signature representing potential novel markers of diffuse interstitial myocardial fibrosis—a critical component of maladaptive tissue remodeling that can lead to clinical HF. Importantly, most proteins within this signature were strongly associated with older age and higher risk for incident HF among a large independent community-based cohort of PWOH. This proteomic signature comprised 39 individual proteins and 1 agnostically defined cluster of 42 proteins, related to T-cell activation, TNF signaling, ephrin signaling, extracellular matrix organization, and cell–cell adhesion. These findings within our SMASH discovery study support the premise that HIV-related immune perturbations are associated with MF regardless of HIV serostatus, and results from MESA suggest that these processes also predict incident HF in the general population.

### HIV, Myocardial Fibrosis, and Heart Failure Converge on Immune Activation

It is well-described that PWH, even when virally suppressed on ART, exhibit chronically greater systemic inflammation and immune activation compared with PWOH. This is characterized by T-cell exhaustion, macrophage activation, and elevated circulating proinflammatory cytokines, all of which have been implicated in MF and HF [8, 19]. For example, mechanical or metabolic cardiac insults stimulate the innate immune system to release proinflammatory and profibrotic cytokines, including TNF-α and transforming growth factor beta (TGF-β), and subsequently activated monocytes/macrophage and T cells can trigger a cascading, cardiopathogenic immune response [8, 19]. Ultimately, these immune processes may alter the cardiac fibroblast environment, resulting in replacement of cardiomyocytes with fibrotic tissue. These findings support and expand upon prior hypotheses asserting that perturbations in these immunologic processes may contribute to excess risk of myocardial disease among PWH.

### Proteins Associated With HIV, Myocardial Interstitial Fibrosis, and Incident Clinical HF

We identified 34 proteins associated with (1) positive HIV serostatus in SMASH; (2) elevated ECV, independent of HIV serostatus in SMASH; and (3) incident clinical HF among PWOH in MESA. We previously reported the associations of 8 of these 34 proteins—CD27, CD48, CRIM1, HAVCR2, JAM2, LAYN, OGN, and TNFRSF1B—with HIV-related left atrial remodeling and incident HF in the same study populations [10]. Notably, these 34 proteins also included 10 that were agnostically identified in both single-protein and cluster-based discovery analyses (including 4 proteins also associated with left atrial remodeling in SMASH—HAVCR2, JAM2, LAYN, TNFRSF1B), strengthening the likelihood of their role in myocardial disease. Current knowledge of these 10 proteins suggests they are involved in immune activation, migratory, and tissue repair processes.

Of particular interest is HAVCR2, a well-described marker of T-cell exhaustion, integral to immune regulation and tolerance [20]. T-cell exhaustion is a characteristic feature of HIV-related immune dysfunction, and elevated HAVCR2 expression has been shown to contribute to immune dysfunction and HIV disease progression, even among virally suppressed PWH on ART [21]. HAVCR2 has also been linked to aging-related T-cell exhaustion [22] and, in the present study, was associated with older age in both SMASH and MESA. Elevated T-cell expression of HAVCR2 has been linked to acute HF and poorer 12-month prognosis [23], and circulating HAVCR2 was recently associated with time to all-cause mortality in a combined analysis of 3 large cohort studies of PWOH [24].

Also of interest is CKAP4, a protein regulated by TGF-β1 shown to positively correlate with activated myofibroblast markers in mouse and human cardiac tissue and to potentially be involved in cardiac fibrosis and remodeling [25, 26]. Circulating CKAP4 was identified in proteomic analyses among a large population of HF patients to have strong positive associations with N-terminal pro-brain natriuretic peptide, HF-related rehospitalization, and all-cause mortality [27].

Three of these 10 proteins belong to the TNF receptor super-family (TNFRSF1B, TNFRSF10B, TNFRSF19), upregulation of which is a hallmark of HIV-associated inflammation and immune activation [28] and has been repeatedly observed in HF [9]. Other proteins among these 10 suggest that immune cell migration in tissue maintenance and repair may be of importance in the overlap between HIV, MF, and HF—specifically, ephrins/receptors, JAM2, and LAYN. Ephrin signaling mediates myriad cellular processes involved in response to tissue injury, including angiogenesis and activation and migration of T cells, B cells, and macrophages [29]. In the present study, the 1 protein cluster of interest included 4 ephrins/receptors, 1 of which was also identified in single-analyte analyses (EPHB4). Ephrin signaling has been linked to several viral life cycles, including HIV [30], as well as cardiomyocyte development, systolic and diastolic function, and TGF-β–mediated cardiac fibrosis [31]. JAM2 is involved in lymphocyte extravasation during inflammation and angiogenesis [32], and LAYN plays a role in focal adhesion, with recent data describing direct interactions with glycan-rich collagens [33] and especially high expression in regulatory T cells and exhausted infiltrating CD8^+^ T cells [34, 35].

### PIGR and FOLR2

We identified 2 proteins associated with all outcomes in both discovery and external populations—PIGR and FOLR2.

PIGR is an Fc receptor critical to mucosal immunity, impairment of which can result in microbial and endotoxin translocation, 1 hypothesized factor contributing to systemic inflammation among PWH [2]. PIGR has also been linked to intracellular epithelial neutralization of HIV and HIV immune evasion [36]. While PIGR has not, to our knowledge, been investigated in myocardial diseases, impaired mucosal immunity has been linked to myocardial fibrosis and dysfunction [9].

FOLR2 is a folate-binding protein expressed primarily by activated monocytes and macrophages [37, 38], elevation of which is a feature of aging-related and HIV-associated inflammation. Evidence suggests that FOLR2 expression is especially high on infiltrating M2-like macrophages [38, 39], overactivation of which has been implicated in tissue fibrosis, including within the myocardium [19] and in correlation studies of ECV among PWH [40, 41]. Notably, the highly specific nature of FOLR2 expression has led to its utilization for cell-specific delivery of therapeutics that could potentially be leveraged for novel applications (eg, HF) [39].

Given that PIGR and FOLR2 were associated in all analytic components of this study, they may represent especially strong candidates for future investigation clarifying their mechanistic role in MF and HF.

### Limitations

While 87% of discovered proteins were associated with incident HF in MESA, only 5% were validated for their cross-sectional association with elevated ECV. One interpretation of these results relates to temporal ambiguity of cross-sectional associations. Given the substantially higher prevalence of elevated ECV in SMASH compared to MESA (52% vs 20%), it is possible the time point in SMASH reflects, on average, a later disease stage. This would be consistent with nonvalidated proteins reflecting a consequence of MF and yet still being predictive of progression to clinical HF. Type II error should also be considered in interpretation, as the method used for multiple testing correction assumes test independence (ie, biological independence of proteins or GO:BP). This may be particularly relevant when interpreting overrepresentation analyses, which test multiple pathways with substantial overlap; for example, 6 proteins identified within this study all mapped to “cell activation,” “leukocyte activation,” “lymphocyte activation,” and “T-cell activation,” which were treated as independent pathway tests. Last, our results may not generalize to PWH living in lower-resource regions, where CVD risk profiles may substantially differ.

## CONCLUSIONS

We discovered an HIV-related plasma proteomic signature that may in part reflect or contribute to myocardial fibrosis and predicted incident HF in an independent community-based cohort. This signature comprised proteins related to T-cell activation, TNF signaling, and tissue maintenance and repair that warrant further investigation. The present findings also suggest that this profile, though elevated among PWH compared to PWOH, may represent risk pathways for HF that are not HIV-specific. If further validated, these proteins may improve clinical risk prediction and help generate hypotheses for novel HF therapeutic targets among both people with and without HIV.

## Supplementary Material

Suppl material 1

Sippl Material 2

Supplementary materials are available at *The Journal of Infectious Diseases* online (http://jid.oxfordjournals.org/). Supplementary materials consist of data provided by the author that are published to benefit the reader. The posted materials are not copyedited. The contents of all supplementary data are the sole responsibility of the authors. Questions or messages regarding errors should be addressed to the author.

## Figures and Tables

**Table 1.** SMASH Participant Characteristics by HIV Serostatus (n = 342)

	Median [IQR] or % (No.)	
Characteristic	PWH (n = 209)	PWOH (n = 133)
Demographics		
Age, y	55 [51, 58]	55 [52, 58]
Female sex at birth	25% (53)	24% (32)
Race and ethnicity		
Black, non-Hispanic	71% (149)	68% (91)
White, non-Hispanic	24% (50)	25% (33)
Hispanic	5% (10)	7% (9)
Education level ≥high school	72% (151)	78% (104)
Substance use		
Smoking status		
Current	54% (112)	42% (56)
Former	26% (55)	41% (55)
Never	20% (42)	17% (22)
Pack-years of smoking^a^	1.00 [0.00, 2.91]	0.38 [0.00, 2.29]
Hazardous alcohol use (AUDIT score >8)	11% (22)	15% (20)
Opioid use^a^	29% (60)	35% (46)
Stimulant use^a^	41% (85)	37% (49)
Clinical factors		
History of cardiovascular disease^b^	6% (12)	5% (7)
Body mass index, kg/m^2^	25.7 [22.9, 29.3]	26.8 [24, 30.8]
Hypertension^c^	51% (106)	53% (71)
Systolic blood pressure, mm Hg	124 [119, 134]	128 [121, 135]
Blood pressure-lowering medication use	34% (72)	35% (46)
Dyslipidemia^d^	61% (128)	56% (74)
Total cholesterol, mg/dL	172 [148, 195]	176 [149, 207]
HDL-c, mg/dL	51 [41, 63]	58 [46, 70]
Lipid-lowering medication use	25% (53)	21% (28)
Diabetes^e^	13% (27)	11% (15)
Diabetes medication use	10% (20)	8% (11)
eGFR, CKD-EPI, mL/min/1.73 m^2^	88 [74, 103]	93 [78, 106]
Hepatitis C diagnosis	24% (50)	21% (28)
Cardiovascular magnetic resonance		
LV ejection fraction, %	71.9 [67.1, 75.6]	72.5 [67.9, 75.7]
LV ejection fraction <50%	2% (4)	0% (0)
LV mass indexed by BSA, mg/m^2^	61.9 [56.8, 68.6]	62.2 [56.3, 67.3]
LV end-diastolic volume indexed by BSA, mL/m^2^	67.7 [58.0, 77.0]	64.6 [54.1, 76.5]
LA volume indexed by BSA, mL/m^2^	28.1 [21.9, 35.8]	26.8 [22.8, 32.9]
Late gadolinium enhancement	37% (78)	34% (45)
Extracellular volume fraction, %	28.9 [26.5, 31.2]	28.2 [26.1, 29.9]
Elevated extracellular volume fraction^f^	57% (119)	45% (60)
T1 time, ms	996 [969, 1026]	1002 [977, 1025]
HIV-related factors		
HIV viral load detectable (HIV RNA >50 copies/mL)	26% (55)	NA
CD4^+^ T-cell count, cells/μL	604 [393, 823]	NA
CD4^+^ nadir T-cell count, cells/μL	271 [148, 389]	NA
History of AIDS diagnosis^g^	20% (28)	NA
ART use	88% (183)	NA
Duration of ART, y	13.0 [5.2, 15.9]	NA
PI base	36% (74)	NA
NNRTI base	26% (55)	NA
INSTI base	25% (51)	NA
Other ART base	1% (3)	NA

Abbreviations: ART, antiretroviral therapy; AUDIT, Alcohol Use Disorders Identification Test; BSA, body surface area; CKD-EPI, Chronic Kidney Disease Epidemiology Collaboration; eGFR, estimated glomerular filtration rate; HDL-c, high-density lipoprotein cholesterol; HIV, human immunodeficiency virus; IQR, interquartile range, reported as (25th, 75th) percentiles; INSTI, integrase strand transfer inhibitor; LA, left atrial; LV, left ventricular; NA, not applicable; NNRTI, nonnucleoside reverse transcriptase inhibitor; PI, protease inhibitor; PWH, people with human immunodeficiency virus; PWOH, people without human immunodeficiency virus; SMASH, Subclinical Myocardial Abnormalities in HIV.

a Reported in the 5 years preceding cardiovascular magnetic resonance imaging (CMR).

b History of cardiovascular disease included prior myocardial infarction (n = 14), angioplasty (n = 2), stent (n = 9), coronary artery bypass graft (n = 3), other heart surgery (n = 4), or heart failure (n = 2) confirmed by medical records.

c Hypertension defined as use of anti-hypertensive medication or systolic blood pressure ≥140 mm Hg or diastolic blood pressure ≥90 mm Hg averaged over the preceding 5 years when available or at the time of CMR study visit.

d Dyslipidemia defined as use of lipid-lowering medication or fasting total cholesterol level ≥200 mg/dL or low-density lipoprotein cholesterol level ≥130 mg/dL or high-density lipoprotein cholesterol level <40 mg/dL or serum triglyceride level ≥150 mg/dL closest to the time of CMR study visit.

e Diabetes defined as use of hypoglycemic medication or fasting serum glucose levels ≥126 mg/dL closest to the time of CMR study visit. Hemoglobin A1C level <6.5% was used to exclude diabetes if fasting glucose levels were not available.

f Elevated extracellular volume fraction defined as ≥30% among women and ≥28% among men.

g History of AIDS-defining malignancy or opportunistic infection.

**Table 2.** Higher Plasma Levels of FOLR2 and PIGR Were Associated With Positive HIV Serostatus, Myocardial Fibrosis Regardless of HIV Serostatus, and Time to Incident Adjudicated Heart Failure in the Community

		Estimate (95% CI)	
Association	No.	FOLR2	PIGR
SMASH discovery study, people with and without HIV in the US			
Mean standardized difference in plasma protein level, PWH vs PWOH^a^	352	0.44 (.26–.62)	0.33 (.15–.51)
PR of elevated ECV per SD increment in plasma protein level, independent of HIV serostatus^b^	342	1.17 (1.06–1.30)	1.14 (1.02–1.28)
MESA external evaluation study, community-based cohort of people without HIV in the US			
PR of elevated ECV per SD increment in plasma protein level^c^	522	1.31 (1.11–1.55)	1.31 (1.10–1.55)
HR for incident HF per SD increment in plasma protein level^d^	3223 (118 cases)	1.25 (1.03–1.51)	1.46 (1.22–1.75)
HR for incident HFpEF per SD increment in plasma protein level^d^	3211 (53 cases)	1.49 (1.14–1.96)	1.46 (1.10–1.92)
HR for incident HFrEF per SD increment in plasma protein level^d^	3211 (40 cases)	0.98 (.70–1.38)	1.47 (1.09–2.00)

All estimates had a false discovery rate <0.05 (Benjamini-Hochberg). Complete modeling results can be found in Supplementary Tables 1, 5, and 11-13.

Abbreviations: CI, confidence interval; ECV, extracellular volume fraction; FOLR2, folate receptor-2; HF, heart failure; HFpEF, heart failure with preserved left ventricular ejection fraction; HFrEF, heart failure with reduced left ventricular ejection fraction; HIV, human immunodeficiency virus; HR, hazard ratio; MESA, Multi-Ethnic Study of Atherosclerosis; PIGR, polymeric immunoglobulin receptor; PR, prevalence ratio; PWH, people with human immunodeficiency virus; PWOH, people without human immunodeficiency virus; SD, standard deviation; SMASH, Subclinical Myocardial Abnormalities in HIV; US, United States.

a Mean difference estimated using linear regression with robust variance, adjusting for age, sex, race, ethnicity, high school diploma, pack-years of smoking, hazardous alcohol use, stimulant use, opioid use, hepatitis C virus infection (HCV), and estimated glomerular filtration rate (eGFR).

b PR estimated using modified Poisson regression, adjusting for age, sex, race, ethnicity, high school diploma, pack-years of smoking, hazardous alcohol use, stimulant use, opioid use, HCV, body mass index (BMI), systolic blood pressure (SBP), anti-hypertensive medication, dyslipidemia, diabetes, and eGFR. Elevated ECV defined as ≥30% among women and ≥28% among men.

c PR estimated using modified Poisson regression, adjusting for study site, age, sex, race, ethnicity, high school diploma, current smoking, heavy alcohol use, BMI, SBP, anti-hypertensive medication, dyslipidemia, diabetes, and eGFR. Elevated ECV defined as ≥30% among women and ≥28% among men (same criterion used in SMASH discovery).

d HR estimated using Cox regression, adjusting for study site, age, sex, race, ethnicity, high school diploma, current smoking, heavy alcohol use, BMI, SBP, anti-hypertensive medication, dyslipidemia, diabetes, and eGFR. There were 118 total incident cases of HF and following exclusion of those without available left ventricular ejection fraction (LVEF) data (n = 12), 53 cases were HFpEF (LVEF ≥50%), 40 were HFrEF (LVEF <40%), and 13 were HF with mid-range EF (LVEF 40%–9%).

## Data Availability

The processed data generated in this study are available in the supplementary files provided with this manuscript. Anonymized individual-level SMASH and MESA data access procedures are in accordance with participant informed consent and National Institutes of Health (NIH) data sharing policies. Given the sensitive nature of data collected in SMASH, requests for data sharing from researchers certified in human subject confidentiality can be submitted to and will be reviewed by the parent cohorts according to established study procedures: https://statepi.jhsph.edu/mwccs/ and https://www.jhsph.edu/research/affiliated-programs/aids-linked-to-the-intravenous-experience/. MESA data are available upon request through BioLINCC (https://biolincc.nhlbi.nih.gov/studies/mesa/) according to established study procedures, and MESA proteomics data, generated as part of the National Heart, Lung, and Blood Institute (NHLBI) Trans-Omics for Precision Medicine (TOPMed) program, are available via the NHLBI database of Genotypes and Phenotypes (dbGaP, accession number: phs001416.v3.p1 NHLBI TOPMed: MESA and MESA Family AA-CAC).
