# Supplementary material for: Human Immunodeficiency Virus–Associated Proteomic Signature of Myocardial Fibrosis and Incident Heart Failure
Source: J Infect Dis. 2026 Jan 12;233(4):e937–47. doi: 10.1093/infdis/jiag013 (PMC12985786; doi:10.1093/infdis/jiag013)
Supplement: jiag013_Supplementary_Data [file jiag013_supplementary_data.zip › Peterson et al HIV ECV HF Supplement CLEAN.pdf]

HIV-Associated Proteomic Signature of Myocardial Fibrosis and Incident Heart Failure

Supplementary Materials

Tess E Peterson, Virginia S Hahn, Ruin Moaddel, Min Zhu, Jinshui Fan, Supriyo De, Sabina A Haberlen, Frank J Palella, Michael Plankey, Joel S Bader, Joao AC Lima, Robert E Gerszten, Jerome I Rotter, Gregory D Kirk, Damani A Piggott, Luigi Ferrucci, Joseph B Margolick, Todd T Brown, Wendy S Post, Katherine C Wu

Table of Contents

Table/Figure	*Page*
Detailed Methods.	3
**TABLE S1.** Cross-sectional associations between plasma protein abundances and HIV serostatus among (A) PWH and PWOH and (B) PWH with undetectable plasma HIV RNA and PWOH in SMASH.	6
**TABLE S2.** Proteins cross-sectionally associated with HIV serostatus corresponding to statistically over-represented biological processes.	7
**TABLE S3.** Individual proteins comprising the HIV-associated protein cluster agnostically defined using weighted gene co-expression network analysis.	9
**TABLE S4.** Individual proteins in HIV-associated protein cluster defined using weighted gene co-expression network analysis corresponding to statistically over-represented biological processes.	10
**TABLE S5.** Cross-sectional associations between HIV-associated plasma protein abundances and elevated myocardial extracellular volume fraction among PWH and PWOH in SMASH.	11
**TABLE S6.** Pairwise Spearman's correlation coefficients between 39 individual proteins associated with positive HIV serostatus and elevated ECV with concordant directionality.	11
**TABLE S7.** Over-representation analysis of proteins cross-sectionally associated with HIV serostatus and elevated myocardial extracellular volume fraction, mapping to Gene Ontology: Biological Process annotations.	11
**TABLE S8.** Percent difference in association between HIV serostatus and elevated myocardial extracellular volume fraction with adjustment for plasma abundance of 39 individual candidate protein contributors in SMASH.	12
**TABLE S9.** Difference in association between plasma abundance of 39 individual proteins of interest and elevated myocardial extracellular volume fraction by HIV serostatus in SMASH.	14
**TABLE S10.** Sensitivity analysis: cross-sectional associations between plasma proteins of interest and elevated myocardial extracellular volume fraction in SMASH, excluding participants with prior myocardial infarction and/or evidence of ischemic scarring on CMR.	16
**TABLE S11.** Sensitivity analysis: cross-sectional associations between plasma proteins of interest and T2 time among a subset of SMASH participants with measured T2.	18
**TABLE S12.** MESA participant characteristics (2010-2012) by analysis.	20
**TABLE S13.** Cross-sectional associations between proteins of interest and elevated myocardial extracellular volume fraction in the Multi-Ethnic Study of Atherosclerosis.	22

**TABLE S14.** Association between proteins of interest and incident clinical heart failure in the Multi-Ethnic Study of Atherosclerosis.	24
**TABLE S15.** Association between proteins of interest and time to incident clinical heart failure with preserved or ejection fraction (HFpEF) and reduced or ejection fraction (HFrEF) in the Multi-Ethnic Study of Atherosclerosis.	26
**FIGURE S1.** Flow diagram of SMASH study participants included in analysis sample.	28
**FIGURE S2.** Spearman's correlation between plasma abundances of 39 proteins independently associated with both HIV seropositivity and elevated myocardial extracellular volume fraction, concordant directionality, among PWH and PWOH in SMASH.	29
**FIGURE S3.** Distribution of plasma protein cluster level by HIV serostatus and plasma HIV RNA in SMASH.	30
**FIGURE S4.** Associations between identified HIV-associated proteomic signature of myocardial fibrosis and clinical characteristics in SMASH.	31
**FIGURE S5.** Flow diagram of MESA study participants included in cross-sectional and longitudinal analysis samples.	32
**FIGURE S6.** Associations between clinical characteristics and validated HIV-associated proteomic signature of myocardial fibrosis and incident heart failure in MESA	33

SUPPLEMENTAL METHODS

Of note, these methods are taken from work previously published by this research group (Peterson et al. *Nat Commun* (2025) **16**, 610) with minimal modification to ensure scientific rigor and reproducibility. This statement provides scientific attribution to this resource. We have not placed quotes (“ ”) around areas that have been reproduced.

SMASH (Discovery) Parent Cohort Details

The Multicenter AIDS Cohort Study (MACS) enrollment began in 1984 and occurred at 4 U.S. sites (Baltimore, MD/Washington, DC; Chicago, IL; Pittsburgh, PA/Columbus, OH; and Los Angeles, CA) over 4 enrollment waves: 1984-1985, 1987-1991, 2001-2003 and 2010-2018.

The Women’s Interagency HIV Study (WIHS) enrollment began in 1994 and recruited women with and without HIV in 4 waves: 1994-1995, 2001-2002, and 2011-2012 from 10 cities (6 original cohort sites were in Brooklyn, NY; the Bronx/Manhattan, NY; Washington, DC; Chicago, IL; San Francisco, CA; and Los Angeles, CA; 4 southern sites were added in 2013: Chapel Hill, NC; Atlanta, GA; Birmingham, AL/Jackson, MS; and Miami, FL. The Los Angeles site discontinued active follow-up in 2013).

MACS and WIHS became the MACS/WIHS Combined Cohort Study (MWCCS) in 2019. (https://statepi.jhsph.edu/mwccs/).

AIDS Linked to the Intravenous Experience (ALIVE) enrollment began in 1988 in Baltimore, MD. Additional cohort recruitment occurred during 1994–1995, 1998, 2000, and 2005–2008.

SMASH (Discovery) Covariate Data Ascertainment and Definitions

Participants completed an interviewer-administered structured questionnaire and biological measures concurrent with cardiovascular magnetic resonance (CMR). Prescribed medications, history of cardiovascular disease (CVD), and substance use during the preceding 5 years were queried. These data were supplemented with data collected through the MACS, WIHS, and ALIVE cohorts, including demographics and CVD risk factors. Among people with HIV (PWH), measures of HIV disease activity included current plasma HIV RNA concentrations (Roche ultrasensitive assay), current and nadir CD4+ T cell counts/µL, history of AIDS-defining malignancy or opportunistic infection, and use of ART, including regimen.

Body mass index: calculated as weight in kilograms divided by the square of height in meters.

Chronic active hepatitis C virus infection: positive antibody and detectable hepatitis C virus ribonucleic acid (RNA).

Diabetes: use of hypoglycemic medications or fasting serum glucose levels ≥126 mg/dL closest to the time of CMR study visit. Hemoglobin A1C level <6.5% was used to exclude diabetes if fasting glucose levels were not available.

Dyslipidemia: use of lipid-lowering medications or fasting total cholesterol level ≥200 mg/dL or low-density lipoprotein (LDL) cholesterol level ≥130 mg/dL or high-density lipoprotein (HDL) cholesterol level <40 mg/dL or serum triglyceride level ≥150 mg/dL closest to the time of CMR study visit.

Educational level: did or did not attain a high school diploma.

Estimated glomerular filtration rate (eGFR): calculated using the CKD Epi (2021) equation.

Hazardous alcohol use: a score of >8 on the Alcohol Use Disorders Identification Test (AUDIT) assessed at the time of CMR.

History of cardiovascular disease: prior myocardial infarction, angioplasty, stent, coronary artery bypass surgery, other heart surgery, or heart failure confirmed by medical records.

Hypertension: use of antihypertensive medications or systolic blood pressure≥140 mmHg or diastolic blood pressure ≥90 mmHg averaged over the preceding 5 years when available or at time of cardiac magnetic resonance imaging (CMR) study visit.

Opioid use: included any of the following by any administration route over the 5 years preceding CMR: methadone (including both prescribed and non-prescribed), heroin, speedball, opioid-based pain medications, prescribed and non-prescribed.

Pack-years of smoking: assessed over the 5 years preceding CMR.

Stimulant use: included any of the following by any administration route over the 5 years preceding CMR: cocaine including crack, speedball, crystal methamphetamine, Phenmetrazine (Preludin), benzedrine, methadrine, uppers, speed, methylphenidate (Ritalin), (dextroamphetamine) Dexedrine, amphetamine/dextroamphetamine (Adderall).

MESA (External) Cohort Details and Methods

Elements of the primary analysis were externally validated in the Multi-Ethnic Study of Atherosclerosis (MESA) (https://www.mesa-nhlbi.org/). The MESA is a prospective population-based cohort initiated in 2000 to study the characteristics and progression of subclinical cardiovascular disease (CVD) among a diverse population in the United States. MESA recruited a total of 6814 participants from six geographic areas across the U.S.—Baltimore City and Baltimore County, Maryland; Chicago, Illinois; Forsyth County, North Carolina; Los Angeles County, California; Northern Manhattan and the Bronx, New York; and St. Paul, Minnesota. At the time of enrollment, participants were 45-84 years of age and had no history of clinical CVD—including coronary artery disease, peripheral vascular disease, cerebrovascular disease, and heart failure.[1] The study protocol was approved by Institutional Review Boards of Columbia University, Johns Hopkins University, Northwestern University, University of California Los Angeles, University of Minnesota, and Wake Forest University; and all participants signed informed consent. The present study utilized data on a subset of MESA participants from cohort exam 5 (2010-2012) with follow-up observed through December 31, 2020.

CMR imaging was performed at exam 5 using 1.5-T scanners (Magnetom Avanto and Magnetom Espree, Siemens Medical Systems, Erlangen, Germany) with six-channel anterior and posterior phased-array torso coil elements. The protocol has been described in detail previously (doi:10.1016/j.jcmg.2021.02.014). In addition to the described protocol for ECV measurement, the protocol included standard acquisition and quantification of cine images for structure and function assessment. One cine horizontal long-axis section (four-chamber view), at least 12 cine short-axis sections from the atria to the cardiac apex, and one cine vertical long-axis section (two-chamber view) using a steady-state free precession pulse sequence. Late gadolinium-enhanced (LGE) short axis images were assessed for presence of LGE, pattern, and amount of LGE.

Proteomics was performed using the Olink Explore 3072 assay on EDTA plasma stored at -80°C using the same standardized laboratory protocol and data quality control, normalization, and calibration methods described in the primary methods and in more detail below. This data was generated through the Trans-Omics for Precision Medicine (TOPMed) program in the laboratory of Dr. Robert Gerzsten.

Information on hospital admissions, outpatient diagnoses, and deaths were ascertained at follow-up telephone interviews conducted every 9-12 months with the participant or a proxy. Medical records and International Classification of Disease (ICD) diagnosis codes were obtained for inpatient and outpatient events, and death certificates were obtained for all deaths.

Medical records for reported heart failure (HF) events were independently reviewed by two physicians. The probable diagnosis of HF required a physician diagnosis, signs or symptoms of HF, and medical treatment for HF. The definite diagnosis of HF also required ≥1 criterion such as pulmonary congestion or edema by chest X-ray; reduced left ventricular (LV) function or dilated LV by echocardiography or ventriculography; or evidence of LV diastolic dysfunction. We included both probable and definite diagnosis of HF. End of follow-up for HF event ascertainment was December 31, 2019.

Olink Proximity Extension Assay and Data Quality Control

The Olink Explore 3072 assay was used in both SMASH and MESA. Olink technology has been described in detail previously (doi:10.1371/journal.pone.0095192). Briefly, DNA oligonucleotide-labeled antibody pairs bind target antigen in solution and, when bound pairwise, hybridize and are extended by a DNA polymerase. This forms a new DNA barcode, which is then amplified and quantified by microfluidic quantitative PCR. This technology differs from standard immunoassays in that it does not rely on spectrometry for quantification, and only matched DNA reporter pairs will be amplified, leading to high specificity by mitigating cross-reactive binding.

Data quality control, normalization, and calibration were carried out using the Normalized Protein eXpression (NPX) software at probe, sample, and plate levels. Specifically, data was normalized using multiple internal assay controls used to monitor immunoreaction, extension, and readout steps, and inter-plate sample controls were used to further normalize for inter-plate variation.

The Olink Explore 3072 platform assays 2924 total proteins. Proteins were excluded from analysis if they did not meet Olink batch release standards (n=54), did not pass internal or external protein quality control standards (n=37), or were detected in <50% of samples (n=239). Following quality control, no SMASH samples were excluded, and 2594 proteins were analyzed with final mean intra- and inter-assay coefficients of variation of 9% and 15%, respectively.

Weighted Gene Co-expression Network Analysis (WGCNA)

We derived unique clusters of highly correlated proteins using the WGCNA package in R (version 4.2) (doi:10.1002/0471142727.mb2010s109, doi:10.1186/1471-2105-9-559). Briefly, unsigned weighted networks of proteins were constructed using pairwise bi-weight midcorrelations of plasma abundance values and a soft threshold transformation that produced an approximately scale-free topology. Next, the topological overlap matrix was calculated and used to hierarchically cluster proteins. A summary plasma abundance measure for each cluster, the eigenprotein, was then derived from the within-network expression correlation matrix using principal component analysis and was used in downstream modeling.

SUPPLEMENTAL TABLE S1. Cross-sectional associations between plasma protein abundances and HIV serostatus among (A) PWH and PWOH and (B) PWH with undetectable plasma HIV RNA and PWOH in the United States.

See Supplemental Excel file 'Supplemental Tables.xlsx'

Previously published in Peterson TE, Hahn VS, Moaddel R, *et al.* Proteomic signature of HIV-associated subclinical left atrial remodeling and incident heart failure. *Nat Commun* **16**, 610 (2025). https://doi.org/10.1038/s41467-025-55911-0

SUPPLEMENTAL TABLE S2. Proteins cross-sectionally associated with HIV serostatus corresponding to statistically over-represented biological processes.

Gene Ontology: Biological Process	Enrichment FDR [a]	Protein Count	Proteins
Cytokine production	0.005	67	ACE2, ADGRG1, AXL, B2M, BST2, BTN2A1, BTN3A2, CCN4, CD14, CD160, CD244, CD274, CD28, CD4, CD40, CD46, CD6, CD74, CD80, CD83, CLEC4A, COL3A1, CRTAM, CX3CL1, CXCL6, DLL1, EPHA2, F11R, F2R, FCGR2B, FLT4, HAVCR2, HGF, IFNL1, IL12RB1, IL18, IL1R1, IL1RL2, IL6ST, IL7, INHBB, KLRK1, LAG3, LGALS9, LILRA2, LILRB4, LY9, MDK, NOS2, PDCD1LG2, PGLYRP2, PLA2G10, PLA2G1B, PTPRC, SEMA7A, SLAMF1, SLAMF6, SPON2, TIGIT, TNFRSF14, TNFRSF1B, TNFRSF21, TNFRSF8, TRIM21, VSIG4, VSIR, XCL1
T cell activation, differentiation, and migration	6.23E-05	58	ADA, B2M, CCL2, CD160, CD27, CD274, CD28, CD300A, CD4, CD46, CD48, CD6, CD7, CD74, CD80, CD83, CD8A, CLEC4A, CRTAM, CTSL, FCGR2B, FGL1, HAVCR2, HLA-DRA, ICAM1, IFNL1, IGFBP2, IL12RB1, IL18, IL1RL2, IL2RA, IL6ST, IL7, ITGAL, KLRK1, LAG3, LGALS9, LILRB4, LY9, MDK, PDCD1LG2, PTPRC, SIRPB1, SLAMF1, SLAMF6, SLAMF7, TGFBR2, TIGIT, TNFRSF14, TNFRSF1B, TNFRSF21, TNFRSF4, TNFRSF9, TNFSF13B, VCAM1, VSIG4, XCL1, VSIR
Regulation of MAPK cascade	0.029	56	ACE2, ADAM9, AIDA, ARHGEF5, BMPER, CCL11, CCL14, CCL15, CCL17, CCL2, CCL20, CCL23, CCL25, CCL4, CCL8, CD27, CD300A, CD4, CD40, CD74, CDH2, CX3CL1, EDA2R, EPHA2, F2R, FAS, FCGR2B, FCRL3, FLT4, GDF15, GH1, GPR37, HAVCR2, HGF, ICAM1, IGFBP3, IGFBP4, LGALS9, LILRB4, LTBR, MARCO, MYDGF, NOTCH1, PDGFRA, PLA2G1B, PTPRC, REN, RNF149, ROBO1, SEMA7A, SLAMF1, SMPD1, TNFRSF11A, TNFRSF19, VEGFA, XCL1
Angiogenesis	0.029	52	ACVRL1, ADGRG1, AMOT, ANGPT2, B4GALT1, BMPER, BSG, CCL11, CCL2, CCN3, CD160, CD40, CDH5, CLEC14A, COL18A1, COL4A1, CX3CL1, CXCL10, CXCL13, CXCL8, DLL1, EPHA2, EPHB4, FLT4, GRN, HGF, HS6ST1, HSPG2, HYAL1, IL18, MDK, MYDGF, NOS3, NOTCH1, NOTCH3, NRCAM, NRP2, PDGFRA, PGF, PTPRM, ROBO1, RSPO3, SCG2, SMOC2, TGFBR2, THBS2, THBS4, TIE1, TNFRSF12A, TYMP, VEGFA, VEGFC
Mononuclear cell migration	1.69E-05	37	ARHGEF5, CCL11, CCL14, CCL15, CCL17, CCL2, CCL20, CCL23, CCL25, CCL27, CCL4, CCL8, CCN3, CRTAM, CSF1, CX3CL1, CXCL10, CXCL11, CXCL12, CXCL13, CXCL16, F11R, ICAM1, ITGAL, ITGB7, JAM2, KLRK1, LGALS9, LGMN, MDK, MSTN, NBL1, SLAMF1, SLAMF8, TNFRSF11A, TNFRSF14, XCL1
Response to tumor necrosis factor	4.34E-04	34	ADAM9, ASAH1, CCL11, CCL14, CCL15, CCL17, CCL2, CCL20, CCL23, CCL25, CCL4, CCL8, CD14, CD40, CX3CL1, CXCL16, CXCL8, EDA2R, FAS, HYAL1, IL18BP, KRT18, SMPD1, TNFRSF11A, TNFRSF14, TNFRSF17, TNFRSF19, TNFRSF1A, TNFRSF1B, TNFRSF21, TNFRSF4, TNFSF13B, VCAM1, XCL1

Granulocyte migration	1.16E-04	32	ADGRE2, BSG, CCL11, CCL14, CCL15, CCL17, CCL2, CCL20, CCL23, CCL25, CCL4, CCL8, CD300A, CD74, CSF1, CX3CL1, CXCL10, CXCL11, CXCL13, CXCL6, CXCL8, CXCL9, IL1R1, ITGB2, MDK, MSTN, PLA2G1B, SCG2, SLAMF1, SLAMF8, THBS4, XCL1
Viral life cycle	0.035	30	ACE2, ATG16L1, AXL, BSG, BST2, CCL2, CCL8, CD28, CD4, CD46, CD74, CD80, CTSL, CXCL8, EPHA2, F11R, HAVCR1, ICAM1, ITGB7, LGALS9, NECTIN2, NOTCH1, P4HB, SCARB2, SIGLEC1, SLAMF1, SMPD1, TNFRSF14, TNFRSF4, TRIM21
Regulation of ERK1/ERK2 cascade	0.037	27	BMPER, CCL11, CCL14, CCL15, CCL17, CCL2, CCL20, CCL23, CCL25, CCL4, CCL8, CD4, CD74, CX3CL1, F2R, FLT4, HAVCR2, ICAM1, LGALS9, MARCO, NOTCH1, PDGFRA, PTPRC, SEMA7A, SLAMF1, TNFRSF11A, XCL1
Response to IL-1	4.03E-04	23	CCL11, CCL14, CCL15, CCL17, CCL2, CCL20, CCL23, CCL25, CCL4, CCL8, CD38, CD40, CX3CL1, CXCL8, HYAL1, IL1R1, IL1RL2, INHBB, LGALS9, SMPD1, TNFRSF11A, XCL1, ZBP1
αβ T cell activation and migration	0.001	23	ADA, CD160, CD274, CD28, CD300A, CD80, CD83, CLEC4A, CRTAM, CTSL, HLA-DRA, IL12RB1, IL18, IL2RA, LGALS9, LILRB4, LY9, PTPRC, SLAMF6, TGFBR2, TNFRSF14, VSIR, XCL1
Interferon-γ production	0.001	21	AXL, BTN3A2, CD14, CD160, CD244, CD274, CRTAM, HAVCR2, IFNL1, IL12RB1, IL18, IL1R1, KLRK1, LGALS9, LILRB4, PDCD1LG2, PGLYRP2, SLAMF1, SLAMF6, VSIR, XCL1
B cell activation	0.007	18	ADA, CD27, CD28, CD300A, CD38, CD40, CD74, FCGR2B, FCRL3, IGLC2, IL7, MZB1, PTPRC, SLAMF8, TNFRSF13B, TNFRSF21, TNFRSF4, TNFSF13B
IL-10 production	0.007	14	CD274, CD28, CD46, CD83, DLL1, FCGR2B, HGF, LGALS9, LILRB4, PDCD1LG2, TIGIT, TNFRSF21, XCL1, VSIR
Natural killer cell mediated cytotoxicity	0.030	13	CD160, CRTAM, HAVCR2, IL18, KLRD1, KLRK1, LAG3, LGALS9, NCR1, NECTIN2, SH2D1A, SLAMF6, SLAMF7
T_{reg} cell differentiation	0.020	8	CD28, CD46, HLA-DRA, LAG3, LGALS9, LILRB4, MDK, VSIR

[a] Estimated using Fisher's exact test, Gene Ontology Biological Processes reference database, and a threshold for significance of Benjamini-Hochberg false discovery rate (FDR)<0.05.

MAPK=mitogen-activated protein kinases; ERK=extracellular signal-regulated kinases.

Previously published in Peterson TE, Hahn VS, Moaddel R, *et al.* Proteomic signature of HIV-associated subclinical left atrial remodeling and incident heart failure. *Nat Commun* **16**, 610 (2025). https://doi.org/10.1038/s41467-025-55911-0

SUPPLEMENTAL TABLE S3. Individual proteins comprising the HIV-associated protein cluster (n=42), agnostically defined using weighted gene co-expression network analysis.

Protein	UniProt
ACVRL1	P37023
B2M	P61769
BSG	P35613
BTN2A1	Q7KYR7
CD46	P15529
CD93	Q9NPY3
CKAP4	Q07065
CLEC14A	Q86T13
CST3	P01034
DLL1	O00548
DSC2	Q02487
EFNA4	P52798
EPHA2	P29317
EPHB4	P54760
EPHB6	O15197
ESAM	Q96AP7
FSTL3	O95633
HAVCR2	Q8TDQ0
IFNGR1	P15260
IGFBP4	P22692
IL10RB	Q08334
JAM2	P57087
LAYN	Q6UX15
LTBR	P36941
NBL1	P41271
NECTIN2	Q92692
NECTIN4	Q96NY8
NPDC1	Q9NQX5
PIK3IP1	Q96FE7
RELT	Q969Z4
SCARB2	Q14108
SHISA5	Q8N114
TGFBR2	P37173
THBD	P07204
TNFRSF10B	O14763
TNFRSF11A	Q9Y6Q6
TNFRSF14	Q92956
TNFRSF19	Q9NS68
TNFRSF1A	P19438
TNFRSF1B	P20333
TNFRSF21	O75509
VSIG4	Q9Y279

Previously published in Peterson TE, *et al.* Proteomic signature of HIV-associated subclinical left atrial remodeling and incident heart failure. *Nat Commun* **16**, 610 (2025).

SUPPLEMENTAL TABLE S4. Individual proteins in HIV-associated protein cluster defined using weighted gene co-expression network analysis corresponding to statistically over-represented biological processes.

Gene Ontology: Biological Process	Cluster Protein Count [a]	Measured Protein Count [b]	Enrichment *p*-value [c]	Enrichment FDR [c]	Proteins
cell-cell adhesion	14	371	4.1E-04	0.035	B2M, CD46, CD93, DSC2, ESAM, FSTL3, HAVCR2, JAM2, NECTIN2, NECTIN4, TGFBR2, TNFRSF14, TNFRSF21, VSIG4
cytokine production	12	293	5.8E-04	0.036	B2M, BSG, BTN2A1, CD46, DLL1, EPHA2, HAVCR2, IFNGR1, TNFRSF14, TNFRSF1B, TNFRSF21, VSIG4
regulation of T cell activation	8	152	0.001	0.038	B2M, CD46, HAVCR2, TGFBR2, TNFRSF14, TNFRSF1B, TNFRSF21, VSIG4
leukocyte cell-cell adhesion	8	178	0.003	0.054	B2M, CD46, HAVCR2, JAM2, TGFBR2, TNFRSF14, TNFRSF21, VSIG4
regulation of T cell proliferation	7	92	2.7E-04	0.029	CD46, HAVCR2, TGFBR2, TNFRSF14, TNFRSF1B, TNFRSF21, VSIG4
response to tumor necrosis factor	6	85	0.001	0.038	TNFRSF11A, TNFRSF14, TNFRSF19, TNFRSF1A, TNFRSF1B, TNFRSF21
regulation of hemopoiesis	6	129	0.010	0.095	B2M, CD46, DLL1, FSTL3, TGFBR2, TNFRSF11A
ephrin receptor signaling pathway	4	15	4.6E-05	0.019	EFNA4, EPHA2, EPHB4, EPHB6
regulation of BMP signaling pathway	4	36	0.002	0.043	ACVRL1, FSTL3, NBL1, MICOS10-NBL1
macrophage activation	4	51	0.006	0.073	CD93, HAVCR2, IFNGR1, VSIG4
heart morphogenesis	4	54	0.007	0.080	ACVRL1, DLL1, EPHB4, TGFBR2
regulation of ECM organization	3	8	1.5E-04	0.022	CST3, TNFRSF1A, TNFRSF1B
tight junction assembly	3	13	7.3E-04	0.037	ACVRL1, EPHA2, ESAM
myeloid dendritic cell activation	3	14	9.2E-04	0.038	HAVCR2, LTBR, TGFBR2
activin receptor signaling pathway	3	18	0.002	0.043	ACVRL1, FSTL3, TGFBR2
interleukin-10 production	3	30	0.009	0.090	CD46, DLL1, TNFRSF21

Previously published in Peterson TE, Hahn VS, Moaddel R, *et al.* Proteomic signature of HIV-associated subclinical left atrial remodeling and incident heart failure. *Nat Commun* **16**, 610 (2025). https://doi.org/10.1038/s41467-025-55911-0

[a] Count of proteins within protein cluster of interest mapping to biological process. *N*=40 of 42 total cluster proteins were successfully mapped.

[b] Count of measured Olink proteins mapping to biological process. *N*=2463 of 2594 total measured proteins were successfully mapped.

[c] Estimated using Fisher's exact test and GO: Biological Processes reference database; false discovery rate (FDR) applied Benjamini-Hochberg procedure

SUPPLEMENTAL TABLE S5. Cross-sectional associations between HIV-associated plasma protein abundances and elevated myocardial extracellular volume fraction among PWH and PWOH in the United States (*n*=342).

See Supplemental Excel file ‘Supplemental Tables.xlsx’

SUPPLEMENTAL TABLE S6. Pairwise Spearman's correlation coefficients between 39 individual proteins associated with positive HIV serostatus and elevated ECV with concordant directionality in SMASH (multivariable models, FDR<0.05).

See Supplemental Excel file ‘Supplemental Tables.xlsx’

SUPPLEMENTAL TABLE S7. Over-representation analysis of proteins cross-sectionally associated with HIV serostatus and elevated myocardial extracellular volume fraction, mapping to Gene Ontology: Biological Process annotations.

See Supplemental Excel file ‘Supplemental Tables.xlsx’

SUPPLEMENTAL TABLE S8. Percent difference in association between HIV serostatus and elevated myocardial extracellular volume fraction with adjustment for plasma abundance of 39 individual candidate protein contributors (*n*=342).

Protein	PR for High ECV, PWH vs. PWOH (95% CI)	*p*-value	Reduction in HIV Association with Protein Adjustment, %
TFF3	1.12 (0.89 to 1.42)	0.336	45.9
CD27	1.12 (0.89 to 1.42)	0.329	45.5
PSAP	1.13 (0.90 to 1.41)	0.287	42.8
REG4	1.13 (0.90 to 1.43)	0.295	42.1
SHISA5	1.15 (0.91 to 1.44)	0.239	36.4
FOLR2	1.15 (0.92 to 1.43)	0.233	36.0
GDF15	1.15 (0.92 to 1.45)	0.226	34.1
IGFBP4	1.16 (0.93 to 1.45)	0.190	30.8
TNFRSF10B	1.16 (0.93 to 1.45)	0.193	30.3
NPC2	1.16 (0.93 to 1.45)	0.187	30.3
CD48	1.16 (0.93 to 1.46)	0.197	30.0
GM2A	1.16 (0.93 to 1.46)	0.193	29.8
CKAP4	1.16 (0.93 to 1.46)	0.191	29.7
EDA2R	1.17 (0.93 to 1.46)	0.171	27.1
EPHB4	1.17 (0.94 to 1.46)	0.164	26.2
TNFRSF1B	1.17 (0.93 to 1.47)	0.169	26.0
OGN	1.17 (0.94 to 1.47)	0.164	25.8
CPXM2	1.17 (0.94 to 1.46)	0.156	25.5
JAM2	1.17 (0.94 to 1.46)	0.156	25.4
HAVCR2	1.17 (0.94 to 1.47)	0.157	24.7
CRIM1	1.17 (0.94 to 1.47)	0.155	24.7
FGL1	1.17 (0.94 to 1.47)	0.157	24.6
CKB	1.18 (0.94 to 1.47)	0.150	23.9
KRT19	1.18 (0.94 to 1.47)	0.153	23.4
WFDC2	1.18 (0.95 to 1.47)	0.143	22.9
TFPI2	1.18 (0.94 to 1.47)	0.146	22.7
IGFBP2	1.18 (0.95 to 1.47)	0.142	22.2
TNFRSF19	1.18 (0.95 to 1.47)	0.139	22.0
LAYN	1.19 (0.95 to 1.48)	0.132	20.3
IGFBPL1	1.19 (0.95 to 1.49)	0.126	18.7
CXCL16	1.19 (0.95 to 1.49)	0.127	18.6
ASAH1	1.19 (0.95 to 1.48)	0.123	18.6
LY6D	1.19 (0.96 to 1.48)	0.118	18.0
TNFRSF12A	1.19 (0.95 to 1.49)	0.121	17.5
PIGR	1.19 (0.96 to 1.49)	0.119	17.4
NCR3LG1	1.19 (0.96 to 1.49)	0.116	16.8
OCLN	1.20 (0.96 to 1.49)	0.116	16.5
PLAUR	1.20 (0.96 to 1.49)	0.108	15.2
COL4A1	1.20 (0.97 to 1.50)	0.100	14.1

Prevalence ratio (PR) for high ECV (≥30% among women and ≥28% among men) estimated using modified Poisson regression, adjusting for age, sex, race, ethnicity, education, body mass index, systolic blood pressure, anti-hypertensive medication, dyslipidemia, diabetes, current hazardous alcohol use, cumulative pack-years of smoking in prior 5 years, stimulant use in prior 5 years, opioid use in prior 5 years, hepatitis C, estimated glomerular filtration rate, and indicated protein. ECV=myocardial extracellular volume fraction; SD=standard deviation; CI=confidence interval; FDR=false discovery rate (Benjamini-Hochberg).

SUPPLEMENTAL TABLE S9. Difference in association between plasma abundance of 39 individual proteins of interest and elevated myocardial extracellular volume fraction by HIV serostatus in SMASH (*n*=342).

Protein	PR for High ECV per SD Increment in Plasma Protein, PWH (95% CI)	PR for High ECV per SD Increment in Plasma Protein, PWOH (95% CI)	Interaction *p*-value	Interaction FDR
ASAH1	1.23 (1.08 to 1.39)	1.03 (0.82 to 1.29)	0.730	0.933
CD27	1.16 (1.03 to 1.32)	1.14 (0.91 to 1.42)	0.548	0.933
CD48	1.16 (1.02 to 1.31)	1.10 (0.84 to 1.42)	0.895	0.953
CKAP4	1.15 (1.02 to 1.29)	1.15 (0.91 to 1.44)	0.664	0.933
CKB	1.10 (0.95 to 1.28)	1.28 (1.05 to 1.57)	0.194	0.933
COL4A1	1.21 (1.07 to 1.36)	1.09 (0.91 to 1.31)	0.735	0.933
CPXM2	1.17 (1.04 to 1.31)	1.14 (0.92 to 1.42)	0.928	0.953
CRIM1	1.12 (0.99 to 1.27)	1.19 (0.93 to 1.54)	0.496	0.933
CXCL16	1.15 (1.01 to 1.31)	1.09 (0.86 to 1.38)	0.575	0.933
EDA2R	1.17 (1.03 to 1.33)	1.12 (0.86 to 1.46)	0.741	0.933
EPHB4	1.12 (1.00 to 1.26)	1.14 (0.93 to 1.41)	0.461	0.933
FGL1	1.10 (0.96 to 1.26)	1.34 (1.08 to 1.68)	0.033	0.933
FOLR2	1.15 (1.02 to 1.30)	1.26 (1.01 to 1.56)	0.384	0.933
GDF15	1.15 (1.03 to 1.29)	1.05 (0.84 to 1.31)	0.886	0.953
GM2A	1.18 (1.03 to 1.35)	1.12 (0.89 to 1.40)	0.813	0.933
HAVCR2	1.15 (1.02 to 1.30)	1.13 (0.93 to 1.37)	0.754	0.933
IGFBP2	1.16 (1.00 to 1.35)	1.26 (1.01 to 1.56)	0.489	0.933
IGFBP4	1.18 (1.06 to 1.32)	1.18 (0.94 to 1.50)	0.413	0.933
IGFBPL1	1.12 (1.00 to 1.26)	1.13 (0.94 to 1.36)	0.552	0.933
JAM2	1.27 (1.12 to 1.44)	1.04 (0.83 to 1.30)	0.164	0.933
KRT19	1.08 (0.96 to 1.21)	1.35 (1.05 to 1.74)	0.089	0.933
LAYN	1.20 (1.07 to 1.34)	1.12 (0.89 to 1.42)	0.586	0.933
LY6D	1.18 (1.07 to 1.30)	1.08 (0.88 to 1.33)	0.595	0.933
NCR3LG1	1.07 (0.95 to 1.21)	1.20 (1.00 to 1.44)	0.293	0.933
NPC2	1.21 (1.06 to 1.39)	1.17 (0.95 to 1.43)	0.801	0.933
OCLN	1.12 (1.00 to 1.26)	1.14 (0.91 to 1.42)	0.676	0.933
OGN	1.17 (1.03 to 1.33)	1.13 (0.90 to 1.42)	0.981	0.981
PIGR	1.22 (1.05 to 1.41)	1.01 (0.83 to 1.23)	0.653	0.933
PLAUR	1.18 (1.03 to 1.34)	1.05 (0.87 to 1.27)	0.909	0.953
PSAP	1.25 (1.09 to 1.43)	1.12 (0.91 to 1.38)	0.720	0.933
REG4	1.12 (0.98 to 1.28)	1.22 (0.97 to 1.52)	0.326	0.933
SHISA5	1.15 (1.02 to 1.29)	1.19 (0.93 to 1.51)	0.606	0.933
TFF3	1.16 (1.04 to 1.28)	1.15 (0.89 to 1.47)	0.690	0.933
TFPI2	1.20 (1.08 to 1.34)	0.97 (0.81 to 1.16)	0.325	0.933
TNFRSF10B	1.17 (1.04 to 1.33)	1.13 (0.92 to 1.38)	0.769	0.933
TNFRSF12A	1.14 (1.03 to 1.27)	1.04 (0.83 to 1.29)	0.754	0.933
TNFRSF19	1.16 (1.04 to 1.31)	1.15 (0.91 to 1.44)	0.700	0.933
TNFRSF1B	1.16 (1.04 to 1.30)	1.01 (0.80 to 1.27)	0.752	0.933
WFDC2	1.13 (0.99 to 1.28)	1.19 (0.99 to 1.43)	0.164	0.933

Prevalence ratio (PR) for high ECV (≥30% among women and ≥28% among men) estimated using modified Poisson regression, adjusting for age, sex, race, ethnicity, education, body mass index, systolic blood pressure, anti-hypertensive medication, dyslipidemia, diabetes, current hazardous alcohol use, cumulative pack-years of smoking in prior 5 years, stimulant use in prior 5 years, opioid use in prior 5 years, hepatitis C, estimated glomerular filtration rate, and indicated protein. Interaction *p*-values and false discovery rates were estimated using an HIV×protein multiplicative interaction in models with the same covariates.

PR=prevalence ratio; ECV=myocardial extracellular volume fraction; SD=standard deviation; CI=confidence interval; FDR=false discovery rate (Benjamini-Hochberg).

SUPPLEMENTAL TABLE S10. Sensitivity analysis: cross-sectional associations between plasma proteins of interest and elevated myocardial extracellular volume fraction in SMASH, excluding participants with prior myocardial infarction and/or evidence of ischemic scarring on CMR (*n*=323)

Protein	Original (no exclusions, n=342)		Excluding Participants with prior MI and/or Ischemic Scar Pattern (n=323)	
	PR (95% CI)	*p*-value	PR (95% CI)	*p*-value
ASAH1	1.16 (1.04 to 1.30)	0.0080	1.16 (1.03 to 1.30)	0.0163
CD27	1.15 (1.03 to 1.28)	0.0100	1.14 (1.02 to 1.28)	0.0267
CD48	1.14 (1.03 to 1.28)	0.0157	1.13 (1.01 to 1.27)	0.0355
CKAP4	1.14 (1.03 to 1.27)	0.0123	1.13 (1.02 to 1.27)	0.0239
CKB	1.16 (1.03 to 1.31)	0.0117	1.16 (1.03 to 1.31)	0.0177
COL4A1	1.18 (1.07 to 1.30)	0.0013	1.19 (1.07 to 1.32)	0.0015
CPXM2	1.16 (1.05 to 1.28)	0.0040	1.14 (1.03 to 1.27)	0.0116
CRIM1	1.15 (1.03 to 1.28)	0.0120	1.15 (1.02 to 1.29)	0.0182
CXCL16	1.13 (1.01 to 1.27)	0.0285	1.12 (1.00 to 1.26)	0.0484
EDA2R	1.16 (1.03 to 1.30)	0.0119	1.16 (1.03 to 1.31)	0.0170
EPHB4	1.14 (1.03 to 1.26)	0.0084	1.14 (1.02 to 1.26)	0.0176
FGL1	1.16 (1.03 to 1.31)	0.0124	1.14 (1.01 to 1.29)	0.0379
FOLR2	1.17 (1.06 to 1.30)	0.0028	1.17 (1.04 to 1.30)	0.0080
GDF15	1.13 (1.02 to 1.24)	0.0177	1.10 (1.00 to 1.23)	0.0610
GM2A	1.16 (1.03 to 1.30)	0.0113	1.16 (1.02 to 1.31)	0.0198
HAVCR2	1.14 (1.03 to 1.27)	0.0092	1.14 (1.03 to 1.27)	0.0134
IGFBP2	1.19 (1.05 to 1.35)	0.0061	1.18 (1.04 to 1.34)	0.0117
IGFBP4	1.19 (1.07 to 1.31)	0.0008	1.19 (1.07 to 1.32)	0.0018
IGFBPL1	1.13 (1.03 to 1.25)	0.0089	1.11 (1.00 to 1.23)	0.0402
JAM2	1.18 (1.07 to 1.31)	0.0011	1.17 (1.06 to 1.30)	0.0025
KRT19	1.13 (1.01 to 1.25)	0.0295	1.15 (1.03 to 1.28)	0.0144
LAYN	1.16 (1.05 to 1.28)	0.0034	1.16 (1.05 to 1.29)	0.0048
LY6D	1.15 (1.05 to 1.27)	0.0028	1.17 (1.06 to 1.30)	0.0020
NCR3LG1	1.12 (1.02 to 1.23)	0.0198	1.12 (1.01 to 1.24)	0.0293
NPC2	1.21 (1.08 to 1.35)	0.0009	1.19 (1.06 to 1.34)	0.0027
OCLN	1.13 (1.02 to 1.25)	0.0167	1.13 (1.02 to 1.26)	0.0223
OGN	1.16 (1.04 to 1.30)	0.0096	1.14 (1.02 to 1.28)	0.0265
PIGR	1.14 (1.02 to 1.28)	0.0227	1.12 (0.99 to 1.27)	0.0700
PLAUR	1.13 (1.01 to 1.27)	0.0322	1.13 (1.00 to 1.27)	0.0444
PSAP	1.21 (1.08 to 1.35)	0.0010	1.21 (1.08 to 1.37)	0.0011
REG4	1.15 (1.03 to 1.29)	0.0172	1.13 (1.00 to 1.27)	0.0478
SHISA5	1.16 (1.05 to 1.30)	0.0054	1.15 (1.03 to 1.29)	0.0131
TFF3	1.15 (1.04 to 1.27)	0.0054	1.14 (1.02 to 1.27)	0.0228
TFPI2	1.12 (1.03 to 1.23)	0.0122	1.12 (1.01 to 1.24)	0.0360
TNFRSF10B	1.17 (1.05 to 1.30)	0.0035	1.18 (1.05 to 1.32)	0.0055
TNFRSF12A	1.12 (1.02 to 1.23)	0.0129	1.11 (1.01 to 1.22)	0.0268
TNFRSF19	1.16 (1.05 to 1.29)	0.0053	1.17 (1.04 to 1.30)	0.0068
TNFRSF1B	1.13 (1.02 to 1.25)	0.0168	1.11 (1.00 to 1.23)	0.0585
WFDC2	1.16 (1.05 to 1.28)	0.0047	1.14 (1.02 to 1.27)	0.0166

Prevalence ratio (PR) for high ECV (≥30% among women and ≥28% among men) estimated per SD increment in indicated protein plasma level using modified Poisson regression, adjusting for age, sex, race, ethnicity, education, body mass index, systolic blood pressure, anti-hypertensive medication, dyslipidemia, diabetes, HIV, hepatitis C, current hazardous alcohol use, cumulative pack-years of smoking in prior 5 years, stimulant use in prior 5 years, opioid use in prior 5 years, and estimated glomerular filtration rate.

PR=prevalence ratio; ECV=myocardial extracellular volume fraction; SD=standard deviation; CI=confidence interval; FDR=false discovery rate (Benjamini-Hochberg).

SUPPLEMENTAL TABLE S11. Sensitivity analysis: cross-sectional associations between plasma proteins of interest and T2 time among a subset of SMASH participants with measured T2 (*n*=81).

Protein	Mean Difference in T2 time (ms) per SD Increment in Plasma Protein (95% CI)	*p*-value	FDR
ASAH1	-0.23 (-1.55 to 1.10)	0.737	0.959
CD27	0.06 (-0.96 to 1.08)	0.909	0.992
CD48	-0.59 (-1.67 to 0.49)	0.287	0.959
CKAP4	-0.47 (-1.52 to 0.58)	0.383	0.959
CKB	0.06 (-1.01 to 1.12)	0.916	0.992
COL4A1	0.31 (-0.81 to 1.42)	0.590	0.959
CPXM2	-0.07 (-0.88 to 0.73)	0.858	0.992
CRIM1	-0.20 (-1.10 to 0.70)	0.666	0.959
CXCL16	-0.76 (-1.70 to 0.18)	0.113	0.917
EDA2R	-0.17 (-1.02 to 0.69)	0.704	0.959
EPHB4	-0.19 (-1.25 to 0.88)	0.734	0.959
FGL1	-0.26 (-1.31 to 0.78)	0.621	0.959
FOLR2	0.16 (-0.71 to 1.02)	0.721	0.959
GDF15	-1.32 (-2.40 to -0.24)	0.017	0.331
GM2A	0.24 (-0.57 to 1.06)	0.559	0.959
HAVCR2	-0.53 (-1.64 to 0.58)	0.350	0.959
IGFBP2	-0.03 (-1.07 to 1.00)	0.952	0.995
IGFBP4	0.00 (-0.94 to 0.94)	1.000	1.000
IGFBPL1	0.57 (-0.34 to 1.48)	0.221	0.959
JAM2	1.03 (0.21 to 1.85)	0.014	0.331
KRT19	-0.62 (-1.68 to 0.43)	0.248	0.959
LAYN	-0.12 (-0.91 to 0.67)	0.768	0.966
LY6D	-0.69 (-1.62 to 0.23)	0.141	0.917
NCR3LG1	-0.51 (-1.74 to 0.72)	0.419	0.959
NPC2	0.30 (-0.66 to 1.27)	0.537	0.959
OCLN	-0.34 (-1.42 to 0.74)	0.536	0.959
OGN	0.49 (-0.35 to 1.32)	0.255	0.959
PIGR	-1.17 (-2.36 to 0.02)	0.054	0.706
PLAUR	-0.44 (-1.65 to 0.78)	0.483	0.959
PSAP	0.10 (-0.79 to 1.00)	0.820	0.992
REG4	0.17 (-0.61 to 0.94)	0.676	0.959
SHISA5	-0.18 (-1.13 to 0.76)	0.704	0.959
TFF3	-0.02 (-0.87 to 0.84)	0.970	0.995
TFPI2	-0.06 (-0.87 to 0.74)	0.875	0.992
TNFRSF10B	-0.80 (-1.82 to 0.22)	0.124	0.917
TNFRSF12A	-0.44 (-1.49 to 0.60)	0.407	0.959
TNFRSF19	0.17 (-0.62 to 0.96)	0.674	0.959
TNFRSF1B	-0.42 (-1.35 to 0.51)	0.373	0.959
WFDC2	-0.24 (-1.06 to 0.57)	0.559	0.959

Mean difference in T2 time (ms) estimated per SD increment in indicated protein plasma level using linear regression with robust variance, adjusting for age, sex, race, ethnicity, education, body mass index, systolic blood pressure, anti-hypertensive medication, dyslipidemia, diabetes, HIV, hepatitis C, current hazardous alcohol use, cumulative pack-years of smoking in prior 5 years, stimulant use in prior 5 years, opioid use in prior 5 years, and estimated glomerular filtration rate.

PR=prevalence ratio; SD=standard deviation; CI=confidence interval; FDR=false discovery rate (Benjamini-Hochberg).

SUPPLEMENTAL TABLE S12. MESA participant characteristics (2010-2012) by analysis.

CHARACTERISTIC	Median [IQR] or % (n)	
	Cross-Sectional ECV Analysis (*n*=522)	Incident Event Analysis (*n*=3223)
Demographics		
Age, years	66 [60, 74]	68 [61, 76]
Female sex at birth	251 (48%)	1693 (52%)
Race and ethnicity	..	..
Black, non-Hispanic	120 (23%)	733 (23%)
White, non-Hispanic	326 (62%)	1355 (42%)
Hispanic	76 (15%)	737 (23%)
Chinese	0 (0%)	398 (12%)
Education level ≥ high school	484 (93%)	2796 (87%)
Clinical Factors		
Smoking status	..	..
Current	48 (9%)	236 (7%)
Former	254 (49%)	1504 (47%)
Never	220 (42%)	1483 (46%)
Pack-years of smoking	0.1 [0.0, 15.5]	0.0 [0.0, 13.0]
Current alcohol use	295 (57%)	1465 (46%)
Heavy alcohol use [a]	56 (11%)	271 (8%)
Body mass index, kg/m^2	28.5 [25.4, 32.2]	27.8 [24.6, 31.5]
Hypertension [b]	305 (58%)	1808 (56%)
Systolic blood pressure, mmHg	119 [109, 133]	119 [109, 136]
Diastolic blood pressure, mmHg	69 [62, 75]	68 [62, 75]
Blood pressure-lowering medication use	277 (53%)	1666 (52%)
Dyslipidemia [c]	392 (75%)	2392 (74%)
Total cholesterol, mg/dL	176 [154, 203]	182 [158, 208]
HDL-cholesterol, mg/dL	51 [42, 62]	53 [44, 64]
Lipid-lowering medication use	223 (43%)	1235 (38%)
Diabetes [d]	92 (18%)	589 (18%)
eGFR, CKD-EPI, mL/min/1.73m^2	71 [62, 81]	70 [59, 80]
Cardiovascular Magnetic Resonance		
LV ejection fraction, %	62.2 [57.0, 66.6]	..
LV ejection fraction <50%	24 (5%)	..
LV mass indexed by BSA, mg/m^2	64.6 [57.4, 74.0]	..
LV end-diastolic volume indexed by BSA, mL/m^2	63.2 [55.6, 72.7]	..
LA volume indexed by BSA, mL/m^2	35.0 [28.4, 42.6]	..
Late gadolinium enhancement	52 (10%)	..
Extracellular volume fraction, %	26.7 [24.6, 28.8]	..
Elevated extracellular volume fraction [e]	105 (20%)	..
T1 time, ms	975 [945, 1002]	..

[a] Heavy alcohol use defined as ≥15 drinks per week among men and ≥8 drinks per week among women.

[b] Hypertension is defined as use of antihypertensive medications or systolic blood pressure≥140 mmHg or diastolic blood pressure ≥90 mmHg.

[c] Dyslipidemia is defined as use of lipid-lowering medications or fasting total cholesterol level ≥200 mg/dL or low-density lipoprotein cholesterol level ≥130 mg/dL or high-density lipoprotein cholesterol level <40 mg/dL or serum triglyceride level ≥150 mg/dL.

[d] Diabetes is defined as use of hypoglycemic medication or fasting serum glucose levels ≥126 mg/dL.

[e] Elevated extracellular volume fraction defined as ≥30% among women and ≥28% among men.

IQR=interquartile range, reported as (25th, 75th) percentiles; LV=left ventricular; LA=left atrial; BSA=body surface area; HDL=high-density lipoprotein; eGFR=estimated glomerular filtration rate.

SUPPLEMENTAL TABLE S13. Cross-sectional associations between proteins of interest and elevated myocardial extracellular volume fraction in the Multi-Ethnic Study of Atherosclerosis (*n*=522)

Protein	Model 1			Model 2		
	Prevalence Ratio of High ECV per SD Increment in Plasma Protein (95% CI)	*p*-value	FDR	Prevalence Ratio of High ECV per SD Increment in Plasma Protein (95% CI)	*p*-value	FDR
ASAH1	0.85 (0.70 to 1.02)	0.072	0.187	0.84 (0.71 to 1.01)	0.060	0.181
CD27	1.23 (1.03 to 1.46)	0.022	0.084	1.21 (1.02 to 1.45)	0.032	0.143
CD48	1.18 (1.00 to 1.39)	0.044	0.156	1.19 (1.02 to 1.40)	0.030	0.143
CKAP4	1.07 (0.90 to 1.29)	0.441	0.567	1.03 (0.85 to 1.24)	0.783	0.925
CKB	1.26 (1.04 to 1.52)	0.016	0.076	1.23 (1.00 to 1.51)	0.054	0.175
COL4A1	1.09 (0.93 to 1.29)	0.276	0.399	1.07 (0.90 to 1.28)	0.438	0.760
CPXM2	0.84 (0.70 to 1.02)	0.081	0.187	0.83 (0.69 to 1.00)	0.049	0.175
CRIM1	1.22 (1.04 to 1.43)	0.014	0.076	1.24 (1.05 to 1.45)	0.010	0.122
CXCL16	1.07 (0.90 to 1.27)	0.465	0.567	1.02 (0.85 to 1.21)	0.863	0.960
EDA2R	1.05 (0.85 to 1.30)	0.648	0.737	0.99 (0.81 to 1.22)	0.960	0.960
EPHB4	1.12 (0.95 to 1.32)	0.192	0.324	1.08 (0.92 to 1.27)	0.345	0.673
FGL1	1.13 (0.91 to 1.39)	0.269	0.399	1.07 (0.88 to 1.31)	0.499	0.760
**FOLR2**	**1.27 (1.08 to 1.49)**	**0.004**	**0.048**	**1.31 (1.11 to 1.55)**	**0.002**	**0.036**
GDF15	1.18 (1.00 to 1.41)	0.057	0.185	1.07 (0.87 to 1.32)	0.506	0.760
GM2A	1.13 (0.94 to 1.37)	0.199	0.324	1.07 (0.88 to 1.31)	0.468	0.760
HAVCR2	1.14 (0.96 to 1.35)	0.142	0.276	1.12 (0.93 to 1.34)	0.223	0.512
IGFBP2	1.27 (1.04 to 1.55)	0.018	0.078	1.25 (1.01 to 1.56)	0.044	0.173
IGFBP4	1.19 (0.98 to 1.46)	0.082	0.187	1.16 (0.94 to 1.43)	0.172	0.420
IGFBPL1	1.03 (0.86 to 1.24)	0.721	0.781	1.01 (0.85 to 1.20)	0.920	0.960
JAM2	1.07 (0.90 to 1.27)	0.452	0.567	1.04 (0.88 to 1.24)	0.629	0.846
KRT19	1.23 (1.05 to 1.44)	0.011	0.074	1.18 (0.99 to 1.40)	0.068	0.189
LAYN	1.10 (0.89 to 1.35)	0.384	0.517	1.03 (0.84 to 1.27)	0.782	0.925
LY6D	1.11 (0.92 to 1.34)	0.272	0.399	1.12 (0.92 to 1.36)	0.256	0.530
NCR3LG1	1.21 (0.98 to 1.50)	0.074	0.187	1.18 (0.94 to 1.48)	0.145	0.377

NPC2	1.11 (0.90 to 1.36)	0.325	0.453	1.04 (0.85 to 1.29)	0.682	0.887
OCLN	1.04 (0.87 to 1.24)	0.661	0.737	0.99 (0.84 to 1.18)	0.947	0.960
OGN	1.03 (0.83 to 1.29)	0.770	0.790	1.02 (0.81 to 1.27)	0.895	0.960
**PIGR**	**1.33 (1.16 to 1.54)**	**5.97E-05**	**0.002**	**1.31 (1.10 to 1.55)**	**0.002**	**0.036**
PLAUR	1.19 (0.98 to 1.44)	0.081	0.187	1.13 (0.91 to 1.40)	0.258	0.530
PSAP	0.85 (0.70 to 1.03)	0.105	0.227	0.78 (0.64 to 0.95)	0.013	0.122
REG4	1.15 (0.97 to 1.38)	0.115	0.236	1.06 (0.88 to 1.28)	0.536	0.767
SHISA5	1.15 (0.93 to 1.42)	0.193	0.324	1.10 (0.88 to 1.37)	0.406	0.754
TFF3	1.21 (1.05 to 1.39)	0.009	0.067	1.18 (1.03 to 1.36)	0.019	0.122
TFPI2	1.06 (0.89 to 1.27)	0.512	0.605	1.03 (0.86 to 1.24)	0.726	0.914
TNFRSF10B	1.14 (0.94 to 1.38)	0.172	0.319	1.06 (0.87 to 1.31)	0.551	0.767
TNFRSF12A	0.98 (0.81 to 1.19)	0.844	0.844	0.93 (0.76 to 1.13)	0.452	0.760
TNFRSF19	1.03 (0.84 to 1.27)	0.753	0.790	0.98 (0.80 to 1.22)	0.886	0.960
TNFRSF1B	1.22 (1.06 to 1.40)	0.007	0.067	1.21 (1.03 to 1.41)	0.018	0.122
WFDC2	1.34 (1.12 to 1.60)	0.001	0.028	1.24 (1.02 to 1.52)	0.033	0.143

Prevalence ratio for high ECV (≥30% among women and ≥28% among men) estimated using modified Poisson regression. Model 1 adjusts for study site, age, sex, race, ethnicity, and estimated glomerular filtration rate. Model 2 further adjusts for education, body mass index, systolic blood pressure, anti-hypertensive medication, hyperlipidemia, diabetes, smoking, and heavy alcohol use.

ECV=myocardial extracellular volume fraction; SD=standard deviation; CI=confidence interval; FDR=false discovery rate (Benjamini-Hochberg).

Bold indicates statistically significant following multiple testing correction (FDR<0.05).

SUPPLEMENTAL TABLE S14. Association between proteins of interest and time to incident clinical heart failure in the Multi-Ethnic Study of Atherosclerosis (*n*=118 incident cases among 3223 participants)

Protein	Model 1			Model 2		
	HR per SD Increment in Plasma Protein (95% CI)	*p*-value	FDR	HR per SD Increment in Plasma Protein (95% CI)	*p*-value	FDR
ASAH1	1.12 (0.94 to 1.34)	0.196	0.207	1.08 (0.90 to 1.29)	0.425	0.425
CD27	**1.68 (1.39 to 2.03)**	**6.35E-08**	**2.48E-07**	**1.66 (1.36 to 2.02)**	**5.33E-07**	**4.16E-06**
CD48	**1.38 (1.16 to 1.65)**	**3.77E-04**	**0.001**	**1.35 (1.13 to 1.62)**	**0.001**	**0.002**
CKAP4	**1.52 (1.25 to 1.84)**	**3.27E-05**	**6.45E-05**	**1.42 (1.16 to 1.75)**	**8.30E-04**	**0.001**
CKB	1.18 (0.95 to 1.47)	0.124	0.139	**1.33 (1.06 to 1.66)**	**0.014**	**0.018**
COL4A1	1.11 (0.92 to 1.34)	0.273	0.273	1.16 (0.96 to 1.41)	0.117	0.124
CPXM2	**1.37 (1.13 to 1.66)**	**0.001**	**0.002**	**1.30 (1.06 to 1.59)**	**0.010**	**0.013**
CRIM1	**1.45 (1.20 to 1.74)**	**1.00E-04**	**1.86E-04**	**1.40 (1.16 to 1.68)**	**4.03E-04**	**7.85E-04**
CXCL16	**1.36 (1.12 to 1.67)**	**0.002**	**0.003**	**1.28 (1.05 to 1.57)**	**0.016**	**0.019**
EDA2R	**1.78 (1.41 to 2.26)**	**1.46E-06**	**4.07E-06**	**1.68 (1.31 to 2.15)**	**4.26E-05**	**1.11E-04**
EPHB4	**1.38 (1.12 to 1.69)**	**0.002**	**0.003**	**1.32 (1.07 to 1.63)**	**0.008**	**0.012**
FGL1	**1.37 (1.14 to 1.65)**	**0.001**	**0.001**	**1.36 (1.12 to 1.65)**	**0.002**	**0.003**
FOLR2	**1.30 (1.07 to 1.57)**	**0.007**	**0.008**	**1.25 (1.03 to 1.51)**	**0.024**	**0.028**
GDF15	**1.68 (1.40 to 2.01)**	**2.33E-08**	**1.30E-07**	**1.57 (1.28 to 1.93)**	**1.93E-05**	**5.80E-05**
GM2A	**1.38 (1.16 to 1.63)**	**1.94E-04**	**3.45E-04**	**1.28 (1.08 to 1.53)**	**0.005**	**0.008**
HAVCR2	**1.44 (1.18 to 1.77)**	**4.44E-04**	**0.001**	**1.34 (1.09 to 1.65)**	**0.006**	**0.009**
IGFBP2	1.14 (0.92 to 1.43)	0.235	0.241	**1.30 (1.03 to 1.65)**	**0.027**	**0.030**
IGFBP4	2.07 (1.67 to 2.57)	**3.03E-11**	**1.18E-09**	**1.89 (1.51 to 2.38)**	**4.33E-08**	**8.43E-07**
IGFBPL1	1.39 (1.15 to 1.68)	**0.001**	**0.001**	**1.29 (1.06 to 1.57)**	**0.011**	**0.014**
JAM2	1.60 (1.29 to 1.97)	**1.22E-05**	**2.81E-05**	**1.58 (1.27 to 1.96)**	**4.76E-05**	**1.16E-04**
KRT19	1.60 (1.34 to 1.93)	**4E-07**	**1.2E-06**	**1.55 (1.28 to 1.87)**	**5.32E-06**	**1.73E-05**
LAYN	1.85 (1.48 to 2.31)	**5.67E-08**	**2.45E-07**	**1.85 (1.48 to 2.33)**	**1.18E-07**	**1.53E-06**
LY6D	**1.24 (1.01 to 1.53)**	**0.041**	**0.049**	1.19 (0.96 to 1.48)	0.104	0.112
NCR3LG1	1.21 (0.98 to 1.48)	0.071	0.082	1.20 (0.97 to 1.47)	0.086	0.096
NPC2	**1.70 (1.42 to 2.02)**	**3.71E-09**	**2.90E-08**	**1.58 (1.31 to 1.91)**	**1.81E-06**	**7.82E-06**

OCLN	**1.62 (1.37 to 1.91)**	**8.40E-09**	**5.46E-08**	**1.54 (1.30 to 1.83)**	**8.34E-07**	**4.65E-06**
OGN	**1.75 (1.38 to 2.21)**	**3.71E-06**	**9.04E-06**	**1.61 (1.26 to 2.07)**	**1.36E-04**	**3.02E-04**
PIGR	**1.51 (1.27 to 1.78)**	**2.27E-06**	**5.91E-06**	**1.46 (1.22 to 1.75)**	**3.72E-05**	**1.04E-04**
PLAUR	**1.52 (1.25 to 1.84)**	**2.50E-05**	**5.41E-05**	**1.43 (1.16 to 1.75)**	**6.64E-04**	**0.001**
PSAP	**1.35 (1.13 to 1.60)**	**0.001**	**0.001**	**1.28 (1.07 to 1.54)**	**0.006**	**0.009**
REG4	**1.63 (1.35 to 1.96)**	**3.41E-07**	**1.11E-06**	**1.57 (1.30 to 1.90)**	**3.35E-06**	**1.19E-05**
SHISA5	**1.73 (1.43 to 2.10)**	**2.89E-08**	**1.41E-07**	**1.63 (1.33 to 2.00)**	**3.34E-06**	**1.19E-05**
TFF3	**1.32 (1.14 to 1.54)**	**3.25E-04**	**5.51E-04**	**1.34 (1.14 to 1.57)**	**3.67E-04**	**7.53E-04**
TFPI2	1.15 (0.95 to 1.40)	0.154	0.167	1.15 (0.95 to 1.41)	0.155	0.159
TNFRSF10B	**1.84 (1.51 to 2.25)**	**1.59E-09**	**2.06E-08**	**1.70 (1.38 to 2.10)**	**6.98E-07**	**4.54E-06**
TNFRSF12A	**1.86 (1.52 to 2.28)**	**2.74E-09**	**2.67E-08**	**1.77 (1.43 to 2.19)**	**1.73E-07**	**1.68E-06**
TNFRSF19	**1.67 (1.31 to 2.12)**	**3.31E-05**	**6.45E-05**	**1.62 (1.26 to 2.07)**	**1.40E-04**	**3.02E-04**
TNFRSF1B	**1.61 (1.35 to 1.92)**	**1.03E-07**	**3.66E-07**	**1.59 (1.32 to 1.92)**	**1.51E-06**	**7.37E-06**
WFDC2	**1.90 (1.55 to 2.33)**	**7.44E-10**	**1.45E-08**	**1.84 (1.48 to 2.28)**	**2.98E-08**	**8.43E-07**

Estimated using Cox proportional hazards. Model 1 adjusts for study site, age, sex, race, ethnicity, and estimated glomerular filtration rate. Model 2 further adjusts for education, body mass index, systolic blood pressure, anti-hypertensive medication, dyslipidemia, diabetes, smoking, and heavy alcohol use. N=118 incident adjudicated clinical heart failure events occurred over a median [interquartile range] follow-up period of 9.8 [1.0] years.

HR=hazard ratio; SD=standard deviation; CI=confidence interval; FDR=false discovery rate (Benjamini-Hochberg).

Bold indicates statistically significant following multiple testing correction (FDR<0.05).

SUPPLEMENTAL TABLE S15. Association between proteins of interest and time to incident clinical heart failure with preserved ejection fraction (HFpEF) and reduced ejection fraction (HFrEF) in the Multi-Ethnic Study of Atherosclerosis (*n*=3211)

Protein	HFpEF (*n*=53 incident cases)			HFrEF (*n*=40 incident cases)		
	HR per SD Increment in Plasma Protein (95% CI)	*p*-value	FDR	HR per SD Increment in Plasma Protein (95% CI)	*p*-value	FDR
ASAH1	1.14 (0.88 to 1.48)	0.326	0.343	1.04 (0.75 to 1.43)	0.822	0.891
CD27	**1.73 (1.29 to 2.34)**	**2.95E-04**	**0.001**	1.34 (0.95 to 1.89)	0.100	0.162
CD48	**1.40 (1.06 to 1.84)**	**0.017**	**0.026**	1.37 (1.02 to 1.86)	0.039	0.129
CKAP4	1.37 (1.00 to 1.89)	0.050	0.059	1.37 (0.97 to 1.93)	0.070	0.140
CKB	**1.48 (1.06 to 2.06)**	**0.021**	**0.030**	1.14 (0.77 to 1.69)	0.505	0.608
COL4A1	1.15 (0.86 to 1.53)	0.352	0.362	1.30 (0.95 to 1.78)	0.107	0.166
CPXM2	**1.42 (1.07 to 1.89)**	**0.014**	**0.026**	1.13 (0.79 to 1.62)	0.506	0.608
CRIM1	**1.38 (1.04 to 1.83)**	**0.024**	**0.033**	1.41 (1.02 to 1.94)	0.036	0.129
CXCL16	1.21 (0.90 to 1.64)	0.212	0.230	1.37 (0.96 to 1.96)	0.079	0.140
EDA2R	**1.56 (1.08 to 2.24)**	**0.017**	**0.026**	**1.75 (1.16 to 2.63)**	**0.007**	**0.049**
EPHB4	**1.38 (1.01 to 1.89)**	**0.041**	**0.049**	1.35 (0.95 to 1.91)	0.094	0.159
FGL1	**1.82 (1.37 to 2.42)**	**4.23E-05**	**2.75E-04**	1.13 (0.83 to 1.55)	0.441	0.573
FOLR2	**1.49 (1.14 to 1.96)**	**0.004**	**0.010**	0.98 (0.70 to 1.38)	0.926	0.926
GDF15	**1.83 (1.34 to 2.48)**	**1.25E-04**	**5.44E-04**	**1.62 (1.16 to 2.25)**	**0.004**	**0.041**
GM2A	**1.36 (1.06 to 1.74)**	**0.017**	**0.026**	1.21 (0.87 to 1.68)	0.267	0.385
HAVCR2	**1.79 (1.31 to 2.44)**	**2.28E-04**	**8.88E-04**	0.98 (0.69 to 1.40)	0.917	0.926
IGFBP2	**1.43 (1.01 to 2.02)**	**0.041**	**0.049**	1.19 (0.79 to 1.77)	0.406	0.545
IGFBP4	**2.06 (1.48 to 2.87)**	**1.91E-05**	**2.39E-04**	1.41 (0.92 to 2.16)	0.114	0.170
IGFBPL1	**1.41 (1.07 to 1.86)**	**0.016**	**0.026**	1.37 (0.98 to 1.93)	0.064	0.140
JAM2	**1.50 (1.07 to 2.09)**	**0.017**	**0.026**	1.61 (1.10 to 2.37)	0.014	0.062
KRT19	**1.71 (1.31 to 2.25)**	**9.62E-05**	**4.69E-04**	1.36 (0.98 to 1.91)	0.069	0.140
LAYN	**1.99 (1.42 to 2.80)**	**7.62E-05**	**4.25E-04**	**1.81 (1.22 to 2.68)**	**0.003**	**0.041**
LY6D	1.27 (0.93 to 1.73)	0.129	0.144	1.11 (0.76 to 1.61)	0.604	0.693
NCR3LG1	1.31 (0.96 to 1.79)	0.084	0.097	1.03 (0.73 to 1.47)	0.865	0.912
NPC2	**1.60 (1.20 to 2.14)**	**0.002**	**0.005**	1.39 (0.99 to 1.96)	0.058	0.140

OCLN	**1.76 (1.38 to 2.24)**	**4.94E-06**	**9.64E-05**	**1.47 (1.11 to 1.96)**	**0.008**	**0.049**
OGN	**1.61 (1.12 to 2.31)**	**0.009**	**0.020**	1.54 (1.00 to 2.37)	0.051	0.140
PIGR	**1.46 (1.10 to 1.92)**	**0.008**	**0.017**	1.47 (1.09 to 2.00)	0.013	0.062
PLAUR	**1.47 (1.07 to 2.00)**	**0.016**	**0.026**	1.43 (1.02 to 2.01)	0.040	0.129
PSAP	**1.32 (1.01 to 1.72)**	**0.040**	**0.049**	1.20 (0.86 to 1.66)	0.285	0.397
REG4	**1.53 (1.16 to 2.01)**	**0.002**	**0.006**	**1.77 (1.26 to 2.47)**	**9.04E-04**	**0.018**
SHISA5	**1.67 (1.23 to 2.28)**	**0.001**	**0.004**	1.44 (0.98 to 2.11)	0.064	0.140
TFF3	**1.42 (1.13 to 1.79)**	**0.003**	**0.007**	1.12 (0.79 to 1.59)	0.515	0.608
TFPI2	1.03 (0.76 to 1.40)	0.836	0.836	1.32 (0.97 to 1.79)	0.073	0.140
TNFRSF10B	**1.96 (1.43 to 2.69)**	**3.06E-05**	**2.39E-04**	1.56 (1.10 to 2.22)	0.013	0.062
TNFRSF12A	**1.49 (1.07 to 2.08)**	**0.017**	**0.026**	**2.30 (1.67 to 3.17)**	**3.14E-07**	**1.23E-05**
TNFRSF19	**1.50 (1.05 to 2.16)**	**0.027**	**0.037**	1.57 (1.01 to 2.43)	0.043	0.129
TNFRSF1B	**1.95 (1.50 to 2.55)**	**8.34E-07**	**3.25E-05**	1.08 (0.75 to 1.57)	0.674	0.751
WFDC2	**1.95 (1.43 to 2.66)**	**2.72E-05**	**2.39E-04**	1.43 (0.96 to 2.11)	0.077	0.140

Estimated using Cox proportional hazards, adjusting for study site, age, sex, race, ethnicity, estimated glomerular filtration rate, education, body mass index, systolic blood pressure, anti-hypertensive medication, dyslipidemia, diabetes, smoking, and heavy alcohol use. N=53 incident adjudicated clinical HFpEF events and N=40 incident HFrEF events occurred over a median [interquartile range] follow-up period of 9.8 [1.0] years. N=13 HFmEF cases observed but not analyzed due to inadequate statistical power, and N=12 incident heart failure cases excluded due to lack of ejection fraction data availability for subtyping. HFpEF=HF with left ventricular ejection fraction (LVEF) ≥50%; HFrEF=LVEF <40%; HFmEF=LVEF 40-49%.

HR=hazard ratio; SD=standard deviation; CI=confidence interval; FDR=false discovery rate (Benjamini-Hochberg).

Bold indicates statistically significant following multiple testing correction (FDR<0.05).

SUPPLEMENTAL FIGURE S1. Flow diagram of SMASH study participants included in analysis sample.

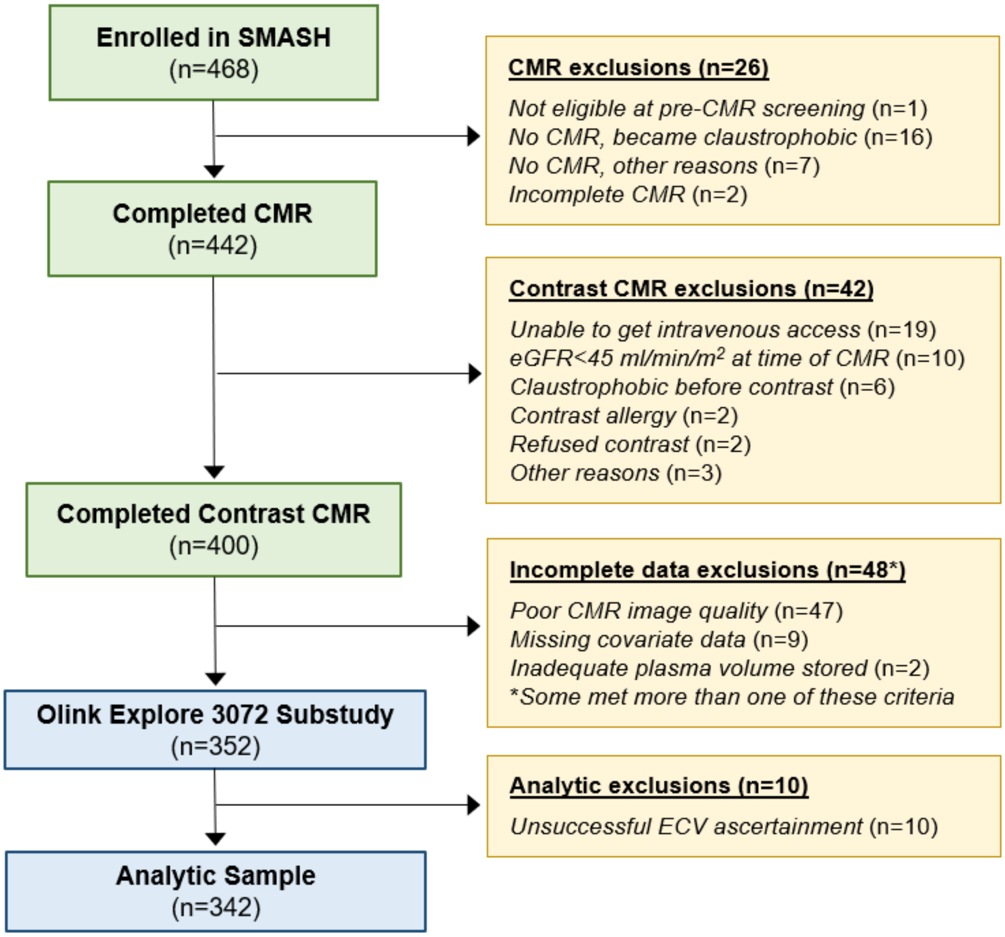

Selection SMASH Olink proteomics substudy included complete cardiovascular magnetic resonance (CMR) with late gadolinium enhancement for the purpose of additional analyses and cost effectiveness.

SUPPLEMENTAL FIGURE S2. Spearman's correlation between plasma abundances of 39 proteins independently associated with both HIV seropositivity and elevated myocardial extracellular volume fraction, concordant directionality, among PWH and PWOH in the United States (*n*=342).

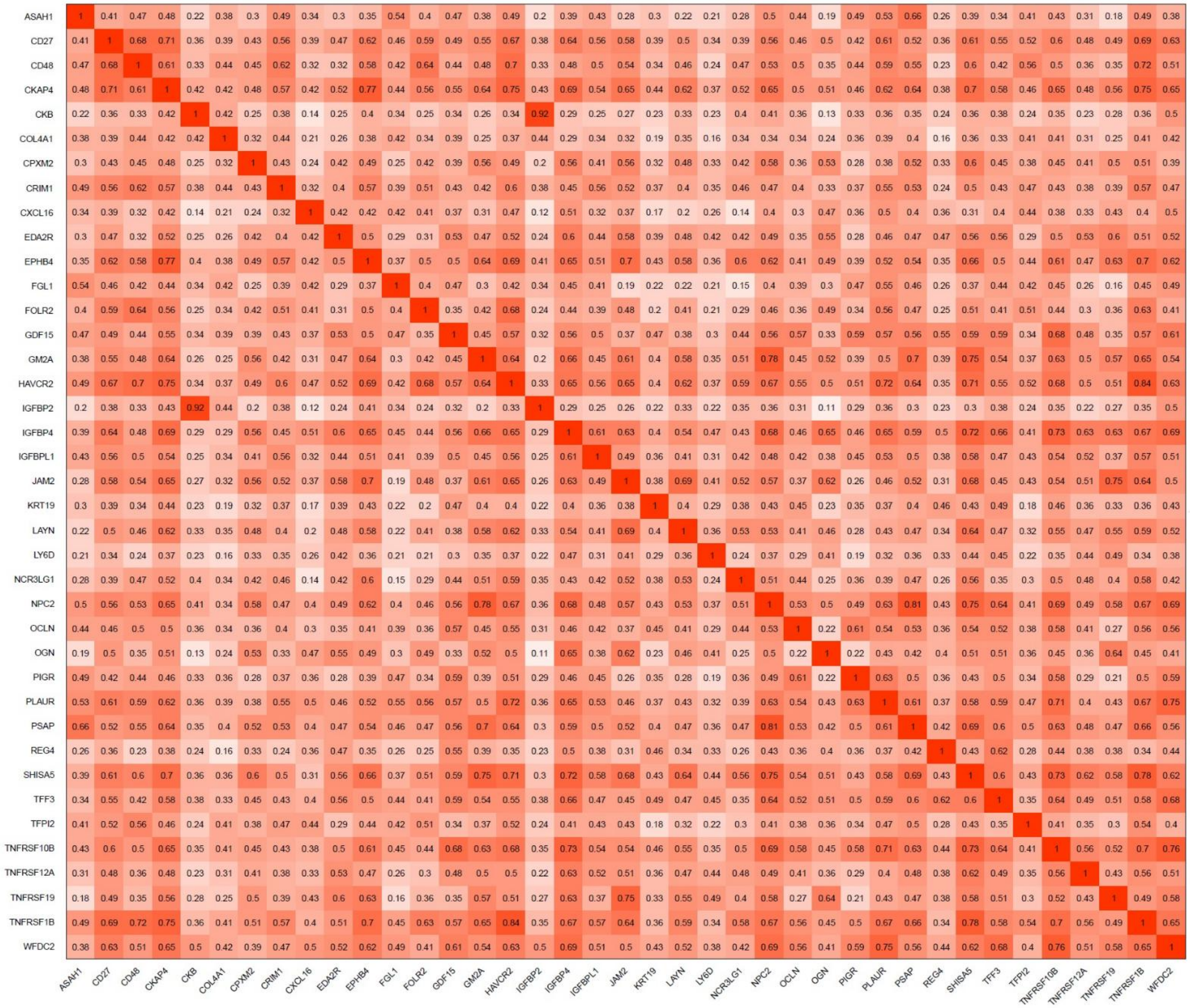

Also viewable as table (Supplemental Table S6) in 'Supplemental Tables.xlsx'

SUPPLEMENTAL FIGURE S3. Unadjusted distribution of plasma protein cluster level by HIV serostatus and plasma HIV RNA in SMASH.

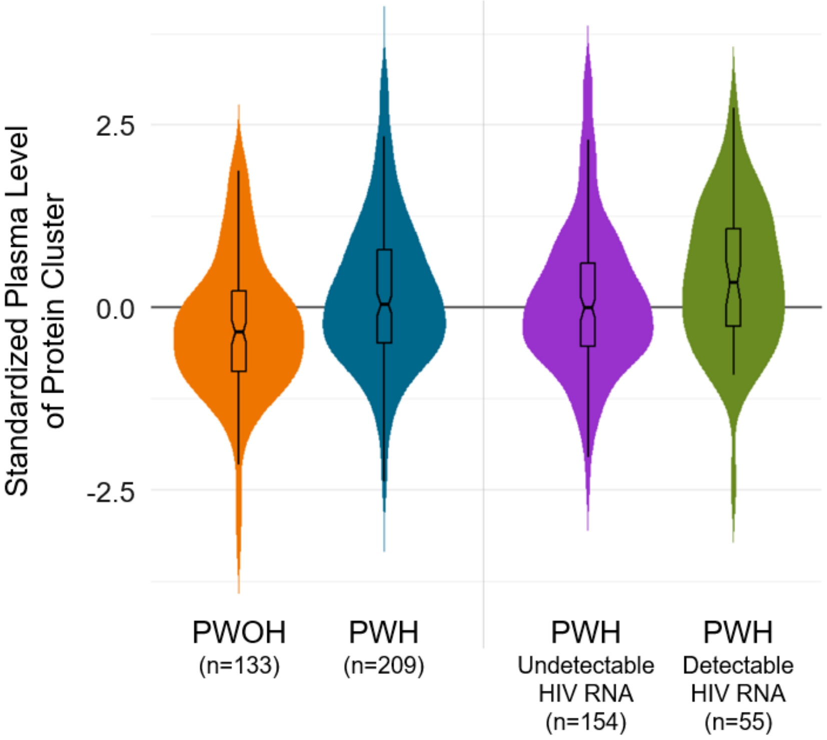

PWOH=people without HIV; PWH=people with HIV; undetectable HIV RNA=plasma RNA ≤50 copies/mL; detectable HIV RNA=plasma RNA >50 copies/mL.

Multivariable adjusted mean difference in plasma cluster level by HIV serostatus was previously published in Peterson TE, Hahn VS, Moaddel R, *et al.* Proteomic signature of HIV-associated subclinical left atrial remodeling and incident heart failure. *Nat Commun* **16**, 610 (2025). https://doi.org/10.1038/s41467-025-55911-0

Estimated using linear regression with robust variance, adjusting for age, sex, race, ethnicity, education level, estimated glomerular filtration rate, hepatitis C virus infection, current hazardous alcohol use, pack-years of smoking in prior 5 years, stimulant use in prior 5 years, and opioid use in prior 5 years.

Mean difference in plasma level comparing PWH to PWOH: 0.48 SD (95% CI: 0.28–0.67; p=3.99E-06).
Comparing PWH with undetectable viral load (HIV plasma RNA ≤50 copies/mL) to PWOH: 0.42 SD (95% CI: 0.21–0.64; p=1.02E-04).

SUPPLEMENTAL FIGURE S4. Associations between identified HIV-associated proteomic signature of myocardial fibrosis and clinical characteristics in SMASH (*n*=342)

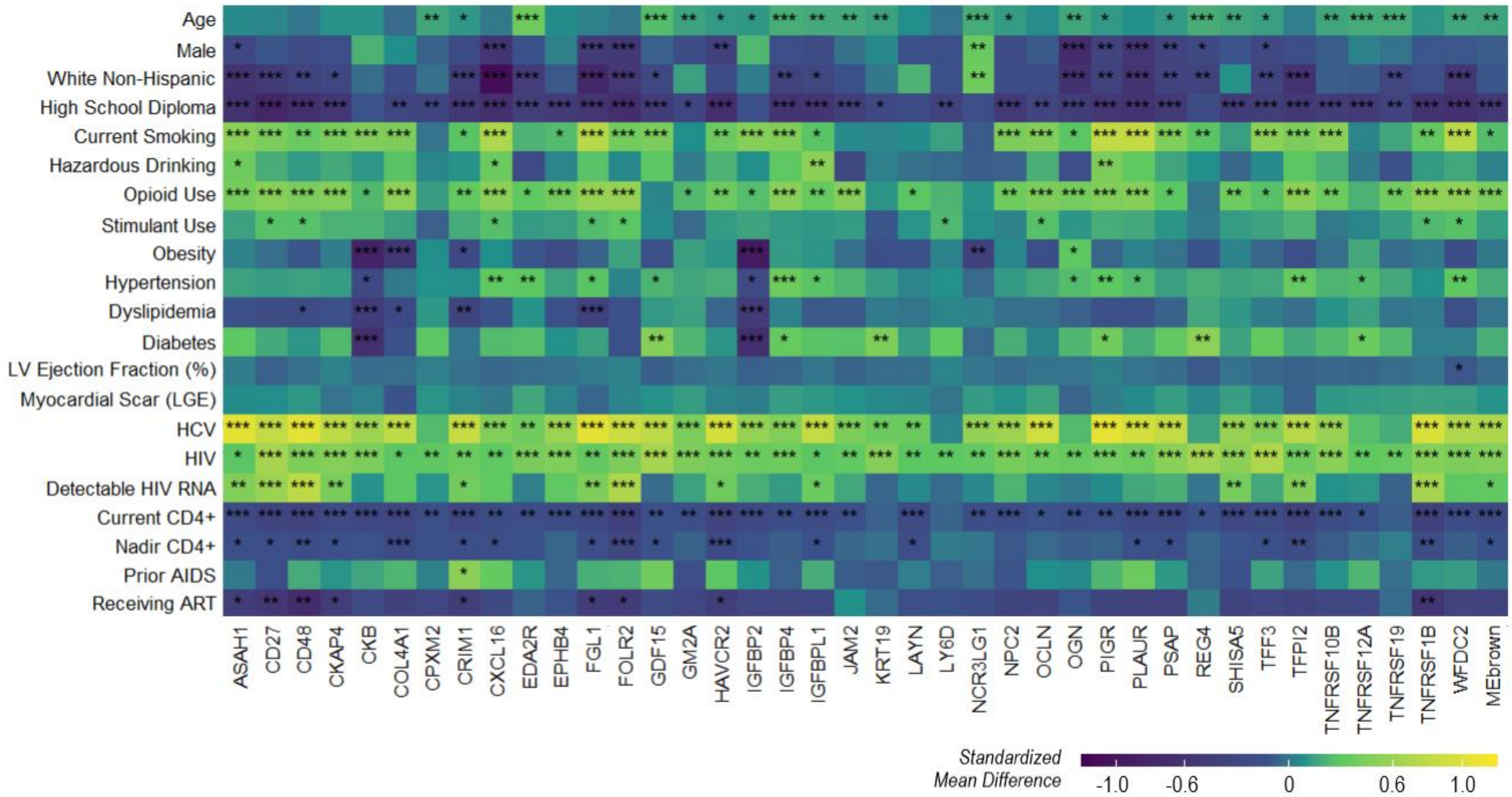

Standardized mean difference in plasma protein abundance among participants with vs. without dichotomous characteristic or per standard deviation increment in continuous characteristic, estimated using linear regression. **p*<0.05, ***p*<0.01, and ****p*<0.001.

LV=left ventricular; LGE=presence of late gadolinium enhancement; HCV=hepatitis C infection; ART=antiretroviral therapy; MEbrown=weighted average of protein cluster plasma abundance.

SUPPLEMENTAL FIGURE S5. Flow diagram of MESA study participants included in cross-sectional and longitudinal analysis samples.

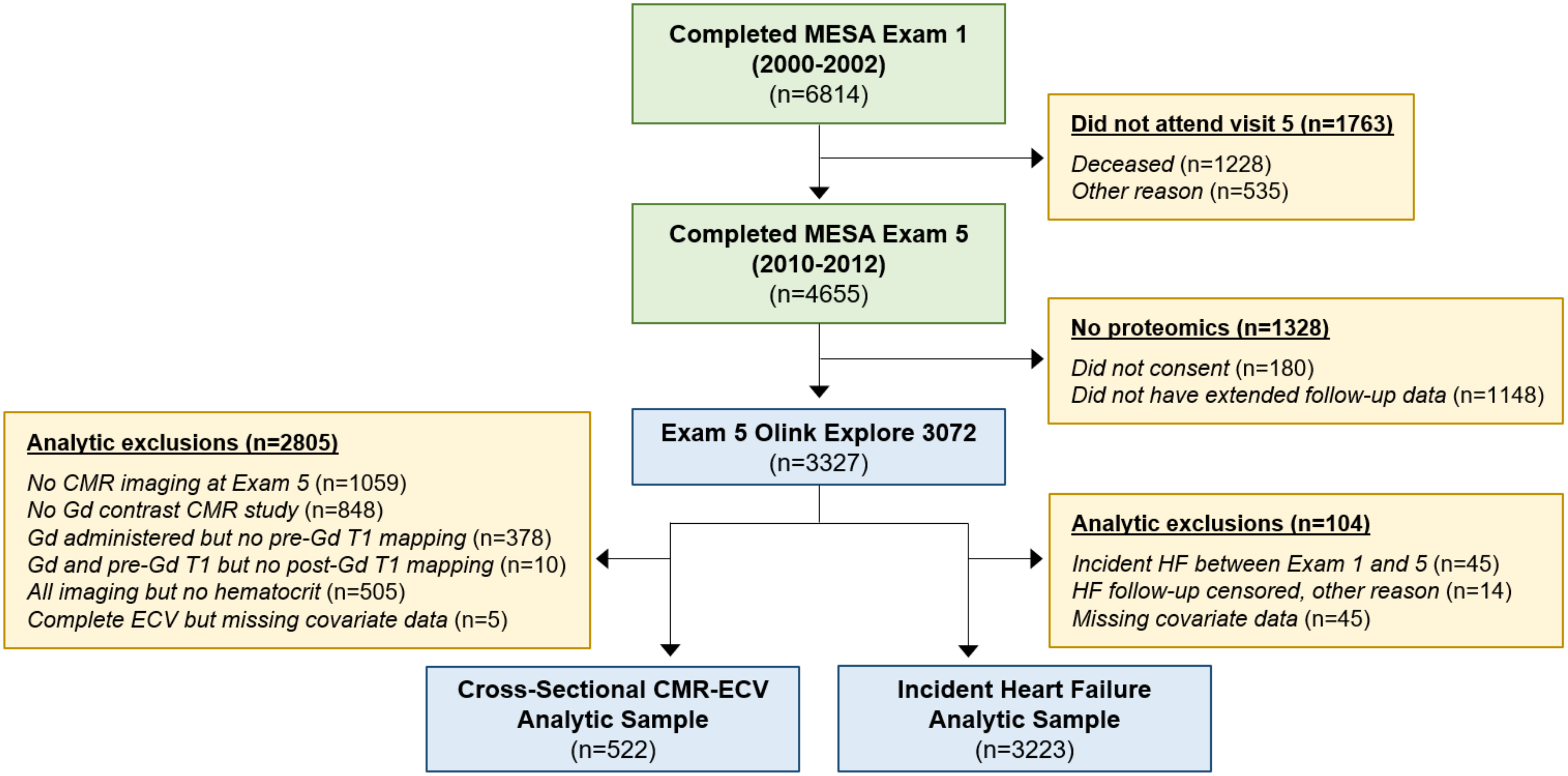

Reflects MESA data availability as of January 2025. CMR=cardiovascular magnetic resonance imaging; Gd=gadolinium-enhanced; ECV=myocardial extracellular volume fraction; HF=heart failure.

SUPPLEMENTAL FIGURE S6. Associations between clinical characteristics and validated HIV-associated proteomic signature of myocardial fibrosis and incident heart failure in MESA (n=3223)

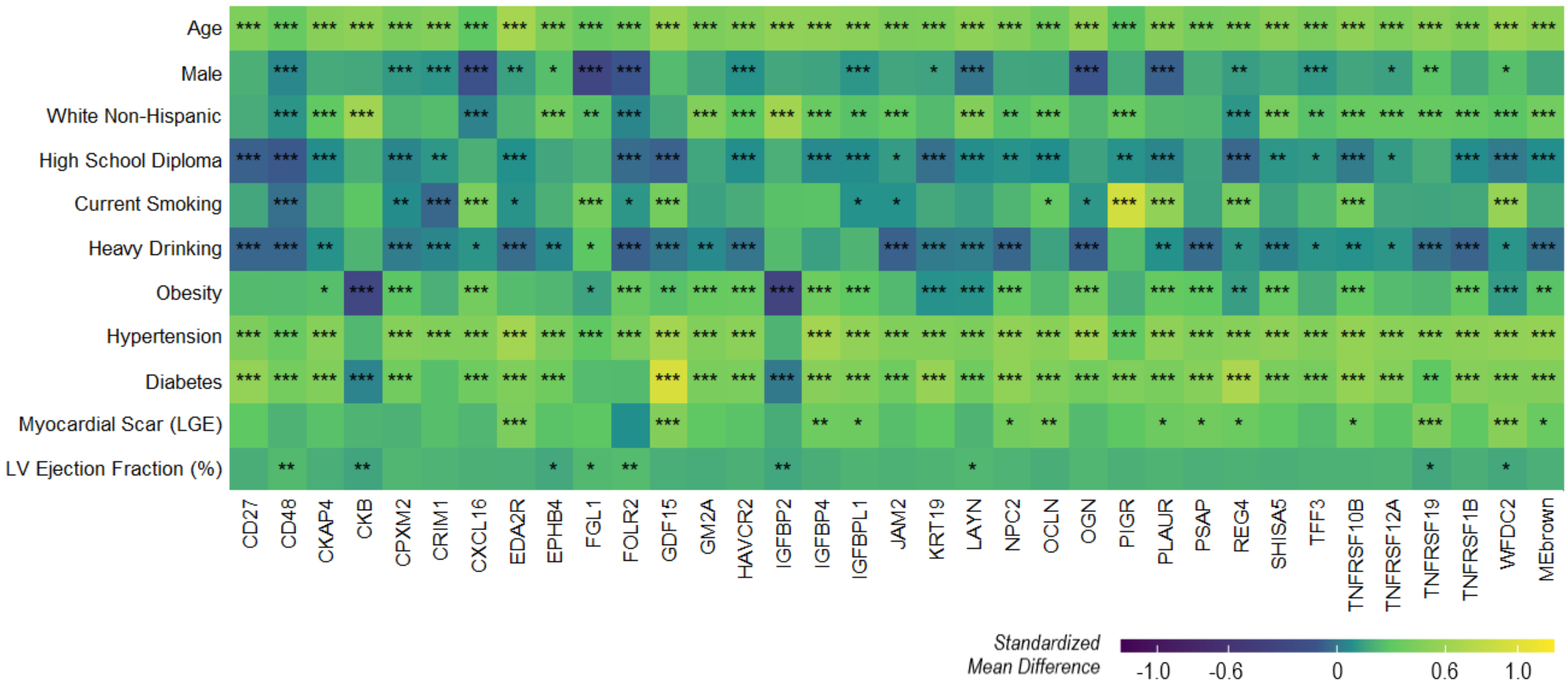

Standardized mean difference in plasma protein abundance among participants with vs. without dichotomous characteristic or per standard deviation increment in continuous characteristic, estimated using linear regression. *p<0.05, **p<0.01, and ***p<0.001.

LV=left ventricular; LGE=presence of late gadolinium enhancement; MEbrown=weighted average of protein cluster plasma abundance.
