# Supplementary material for: Human Immunodeficiency Virus–Associated Proteomic Signature of Myocardial Fibrosis and Incident Heart Failure
Source: J Infect Dis. Author manuscript; Available in PMC 2026 Mar 14. (PMC12985786; doi:10.1093/infdis/jiag013)
Supplement: Supplementary file 1 [file NIHMS2149792-supplement-Sippl_Material_2.docx]

HIV-Associated Proteomic Signature of Myocardial Fibrosis and 

Incident Heart Failure 

**Supplementary Materials**

Tess E Peterson, Virginia S Hahn, Ruin Moaddel, Min Zhu, Jinshui Fan, Supriyo De, Sabina A Haberlen, Frank J Palella, Michael Plankey, Joel S Bader, Joao AC Lima, Robert E Gerszten, Jerome I Rotter, Gregory D Kirk, Damani A Piggott, Luigi Ferrucci, Joseph B Margolick, 

Todd T Brown, Wendy S Post, Katherine C Wu

**Table of Contents**

| *Table/Figure* | *Page* |
| --- | --- |
| Detailed Methods. | 3 |
| **TABLE S1.** Cross-sectional associations between plasma protein abundances and HIV serostatus among (A) PWH and PWOH and (B) PWH with undetectable plasma HIV RNA and PWOH in SMASH. | 6 |
| **TABLE S2.** Proteins cross-sectionally associated with HIV serostatus corresponding to statistically over-represented biological processes. | 7 |
| **TABLE S3.** Individual proteins comprising the HIV-associated protein cluster agnostically defined using weighted gene co-expression network analysis. | 9 |
| **TABLE S4.** Individual proteins in HIV-associated protein cluster defined using weighted gene co-expression network analysis corresponding to statistically over-represented biological processes. | 10 |
| **TABLE S5.** Cross-sectional associations between HIV-associated plasma protein abundances and elevated myocardial extracellular volume fraction among PWH and PWOH in SMASH. | 11 |
| **TABLE S6.** Pairwise Spearman's correlation coefficients between 39 individual proteins associated with positive HIV serostatus and elevated ECV with concordant directionality. | 11 |
| **TABLE S7.** Over-representation analysis of proteins cross-sectionally associated with HIV serostatus and elevated myocardial extracellular volume fraction, mapping to Gene Ontology: Biological Process annotations. | 11 |
| **TABLE S8.** Percent difference in association between HIV serostatus and elevated myocardial extracellular volume fraction with adjustment for plasma abundance of 39 individual candidate protein contributors in SMASH. | 12 |
| **TABLE S9.** Difference in association between plasma abundance of 39 individual proteins of interest and elevated myocardial extracellular volume fraction by HIV serostatus in SMASH. | 14 |
| **TABLE S10.** Sensitivity analysis: cross-sectional associations between plasma proteins of interest and elevated myocardial extracellular volume fraction in SMASH, excluding participants with prior myocardial infarction and/or evidence of ischemic scarring on CMR. | 16 |
| **TABLE S11.** Sensitivity analysis: cross-sectional associations between plasma proteins of interest and T2 time among a subset of SMASH participants with measured T2. | 18 |
| **TABLE S12.** MESA participant characteristics (2010-2012) by analysis. | 20 |
| **TABLE S13.** Cross-sectional associations between proteins of interest and elevated myocardial extracellular volume fraction in the Multi-Ethnic Study of Atherosclerosis. | 22 |
| **TABLE S14.** Association between proteins of interest and incident clinical heart failure in the Multi-Ethnic Study of Atherosclerosis. | 24 |
| **TABLE S15.** Association between proteins of interest and time to incident clinical heart failure with preserved or ejection fraction (HFpEF) and reduced or ejection fraction (HFrEF) in the Multi-Ethnic Study of Atherosclerosis. | 26 |
| **FIGURE S1.** Flow diagram of SMASH study participants included in analysis sample. | 28 |
| **FIGURE S2.** Spearman’s correlation between plasma abundances of 39 proteins independently associated with both HIV seropositivity and elevated myocardial extracellular volume fraction, concordant directionality, among PWH and PWOH in SMASH. | 29 |
| **FIGURE S3.** Distribution of plasma protein cluster level by HIV serostatus and plasma HIV RNA in SMASH. | 30 |
| **FIGURE S4.** Associations between identified HIV-associated proteomic signature of myocardial fibrosis and clinical characteristics in SMASH. | 31 |
| **FIGURE S5.** Flow diagram of MESA study participants included in cross-sectional and longitudinal analysis samples. | 32 |
| **FIGURE S6.** Associations between clinical characteristics and validated HIV-associated proteomic signature of myocardial fibrosis and incident heart failure in MESA | 33 |

**SUPPLEMENTAL METHODS**

Of note, these methods are taken from work previously published by this research group (Peterson et al. *Nat Commun* (2025) **16**, 610) with minimal modification to ensure scientific rigor and reproducibility. This statement provides scientific attribution to this resource. We have not placed quotes (“ ”) around areas that have been reproduced.

SMASH (Discovery) Parent Cohort Details

The Multicenter AIDS Cohort Study (MACS) enrollment began in 1984 and occurred at 4 U.S. sites (Baltimore, MD/Washington, DC; Chicago, IL; Pittsburgh, PA/Columbus, OH; and Los Angeles, CA) over 4 enrollment waves: 1984-1985, 1987-1991, 2001-2003 and 2010-2018. 

The Women’s Interagency HIV Study (WIHS) enrollment began in 1994 and recruited women with and without HIV in 4 waves: 1994-1995, 2001-2002, and 2011-2012 from 10 cities (6 original cohort sites were in Brooklyn, NY; the Bronx/Manhattan, NY; Washington, DC; Chicago, IL; San Francisco, CA; and Los Angeles, CA; 4 southern sites were added in 2013: Chapel Hill, NC; Atlanta, GA; Birmingham, AL/Jackson, MS; and Miami, FL. The Los Angeles site discontinued active follow-up in 2013).

MACS and WIHS became the MACS/WIHS Combined Cohort Study (MWCCS) in 2019. ([https://statepi.jhsph.edu/mwccs/](https://statepi.jhsph.edu/mwccs/)).

AIDS Linked to the Intravenous Experience (ALIVE) enrollment began in 1988 in Baltimore, MD.  Additional cohort recruitment occurred during 1994–1995, 1998, 2000, and 2005–2008.  

SMASH (Discovery) Covariate Data Ascertainment and Definitions

Participants completed an interviewer-administered structured questionnaire and biological measures concurrent with cardiovascular magnetic resonance (CMR). Prescribed medications, history of cardiovascular disease (CVD), and substance use during the preceding 5 years were queried. These data were supplemented with data collected through the MACS, WIHS, and ALIVE cohorts, including demographics and CVD risk factors. Among people with HIV (PWH), measures of HIV disease activity included current plasma HIV RNA concentrations (Roche ultrasensitive assay), current and nadir CD4+ T cell counts/µL, history of AIDS-defining malignancy or opportunistic infection, and use of ART, including regimen.

*Body mass index*: calculated as weight in kilograms divided by the square of height in meters.

*Chronic active hepatitis C virus infection*: positive antibody and detectable hepatitis C virus ribonucleic acid (RNA).  

*Diabetes*: use of hypoglycemic medications or fasting serum glucose levels ≥126 mg/dL closest to the time of CMR study visit. Hemoglobin A1C level <6.5% was used to exclude diabetes if fasting glucose levels were not available. 

*Dyslipidemia*: use of lipid-lowering medications or fasting total cholesterol level ≥200 mg/dL or low-density lipoprotein (LDL) cholesterol level ≥130 mg/dL or high-density lipoprotein (HDL) cholesterol level <40 mg/dL or serum triglyceride level ≥150 mg/dL closest to the time of CMR study visit. 

*Educational level*: did or did not attain a high school diploma.

*Estimated glomerular filtration rate (eGFR)*: calculated using the CKD Epi (2021) equation. 

*Hazardous alcohol use*: a score of >8 on the Alcohol Use Disorders Identification Test (AUDIT) assessed at the time of CMR. 

*History of cardiovascular disease*: prior myocardial infarction, angioplasty, stent, coronary artery bypass surgery, other heart surgery, or heart failure confirmed by medical records.

*Hypertension*: use of antihypertensive medications or systolic blood pressure≥140 mmHg or diastolic blood pressure ≥90 mmHg averaged over the preceding 5 years when available or at time of cardiac magnetic resonance imaging (CMR) study visit. 

*Opioid use*: included any of the following by any administration route over the 5 years preceding CMR: methadone (including both prescribed and non-prescribed), heroin, speedball, opioid-based pain medications, prescribed and non-prescribed.

*Pack-years of smoking*: assessed over the 5 years preceding CMR. 

*Stimulant use*: included any of the following by any administration route over the 5 years preceding CMR: cocaine including crack, speedball, crystal methamphetamine, Phenmetrazine (Preludin), benzedrine, methadrine, uppers, speed, methylphenidate (Ritalin), (dextroamphetamine) Dexedrine, amphetamine/dextroamphetamine (Adderall).

MESA (External) Cohort Details and Methods

Elements of the primary analysis were externally validated in the Multi-Ethnic Study of Atherosclerosis (MESA) ([https://www.mesa-nhlbi.org/](https://www.mesa-nhlbi.org/)). The MESA is a prospective population-based cohort initiated in 2000 to study the characteristics and progression of subclinical cardiovascular disease (CVD) among a diverse population in the United States. MESA recruited a total of 6814 participants from six geographic areas across the U.S.—Baltimore City and Baltimore County, Maryland; Chicago, Illinois; Forsyth County, North Carolina; Los Angeles County, California; Northern Manhattan and the Bronx, New York; and St. Paul, Minnesota. At the time of enrollment, participants were 45-84 years of age and had no history of clinical CVD—including coronary artery disease, peripheral vascular disease, cerebrovascular disease, and heart failure.^1^ The study protocol was approved by Institutional Review Boards of Columbia University, Johns Hopkins University, Northwestern University, University of California Los Angeles, University of Minnesota, and Wake Forest University; and all participants signed informed consent. The present study utilized data on a subset of MESA participants from cohort exam 5 (2010-2012) with follow-up observed through December 31, 2020.

CMR imaging was performed at exam 5 using 1.5-T scanners (Magnetom Avanto and Magnetom Espree, Siemens Medical Systems, Erlangen, Germany) with six-channel anterior and posterior phased-array torso coil elements. The protocol has been described in detail previously (doi:10.1016/j.jcmg.2021.02.014). In addition to the described protocol for ECV measurement, the protocol included standard acquisition and quantification of cine images for structure and function assessment. One cine horizontal long-axis section (four-chamber view), at least 12 cine short-axis sections from the atria to the cardiac apex, and one cine vertical long-axis section (two-chamber view) using a steady-state free precession pulse sequence. Late gadolinium-enhanced (LGE) short axis images were assessed for presence of LGE, pattern, and amount of LGE.   

Proteomics was performed using the Olink Explore 3072 assay on EDTA plasma stored at -80°C using the same standardized laboratory protocol and data quality control, normalization, and calibration methods described in the primary methods and in more detail below. This data was generated through the Trans-Omics for Precision Medicine (TOPMed) program in the laboratory of Dr. Robert Gerzsten.

Information on hospital admissions, outpatient diagnoses, and deaths were ascertained at follow-up telephone interviews conducted every 9-12 months with the participant or a proxy. Medical records and International Classification of Disease (ICD) diagnosis codes were obtained for inpatient and outpatient events, and death certificates were obtained for all deaths. 

Medical records for reported heart failure (HF) events were independently reviewed by two physicians. The probable diagnosis of HF required a physician diagnosis, signs or symptoms of HF, and medical treatment for HF. The definite diagnosis of HF also required ≥1 criterion such as pulmonary congestion or edema by chest X-ray; reduced left ventricular (LV) function or dilated LV by echocardiography or ventriculography; or evidence of LV diastolic dysfunction. We included both probable and definite diagnosis of HF. End of follow-up for HF event ascertainment was December 31, 2019.

Olink Proximity Extension Assay and Data Quality Control

The Olink Explore 3072 assay was used in both SMASH and MESA. Olink technology has been described in detail previously (doi:10.1371/journal.pone.0095192). Briefly, DNA oligonucleotide-labeled antibody pairs bind target antigen in solution and, when bound pairwise, hybridize and are extended by a DNA polymerase. This forms a new DNA barcode, which is then amplified and quantified by microfluidic quantitative PCR. This technology differs from standard immunoassays in that it does not rely on spectrometry for quantification, and only matched DNA reporter pairs will be amplified, leading to high specificity by mitigating cross-reactive binding. 

Data quality control, normalization, and calibration were carried out using the Normalized Protein eXpression (NPX) software at probe, sample, and plate levels. Specifically, data was normalized using multiple internal assay controls used to monitor immunoreaction, extension, and readout steps, and inter-plate sample controls were used to further normalize for inter-plate variation.

The Olink Explore 3072 platform assays 2924 total proteins. Proteins were excluded from analysis if they did not meet Olink batch release standards (*n*=54), did not pass internal or external protein quality control standards (*n*=37), or were detected in <50% of samples (*n*=239). Following quality control, no SMASH samples were excluded, and 2594 proteins were analyzed with final mean intra- and inter-assay coefficients of variation of 9% and 15%, respectively.

Weighted Gene Co-expression Network Analysis (WGCNA)

We derived unique clusters of highly correlated proteins using the WGCNA package in R (version 4.2) (doi:10.1002/0471142727.mb2010s109, doi:10.1186/1471-2105-9-559). Briefly, unsigned weighted networks of proteins were constructed using pairwise bi-weight midcorrelations of plasma abundance values and a soft threshold transformation that produced an approximately scale-free topology. Next, the topological overlap matrix was calculated and used to hierarchically cluster proteins. A summary plasma abundance measure for each cluster, the eigenprotein, was then derived from the within-network expression correlation matrix using principal component analysis and was used in downstream modeling.

**SUPPLEMENTAL TABLE S1.** Cross-sectional associations between plasma protein abundances and HIV serostatus among (A) PWH and PWOH and (B) PWH with undetectable plasma HIV RNA and PWOH in the United States.

See Supplemental Excel file ‘Supplemental Tables.xlsx’

Previously published in Peterson TE, Hahn VS, Moaddel R, *et al.* Proteomic signature of HIV-associated subclinical left atrial remodeling and incident heart failure. *Nat Commun* **16**, 610 (2025). [https://doi.org/10.1038/s41467-025-55911-0](https://doi.org/10.1038/s41467-025-55911-0)

**SUPPLEMENTAL TABLE S2.** Proteins cross-sectionally associated with HIV serostatus corresponding to statistically over-represented biological processes. 

| Gene Ontology:    Biological Process | Enrichment FDR ^a^ | Protein Count | Proteins |
| --- | --- | --- | --- |
| Cytokine production | 0.005 | 67 | ACE2, ADGRG1, AXL, B2M, BST2, BTN2A1, BTN3A2, CCN4, CD14, CD160, CD244, CD274, CD28, CD4, CD40, CD46, CD6, CD74, CD80, CD83, CLEC4A, COL3A1, CRTAM, CX3CL1, CXCL6, DLL1, EPHA2, F11R, F2R, FCGR2B, FLT4, HAVCR2, HGF, IFNL1, IL12RB1, IL18, IL1R1, IL1RL2, IL6ST, IL7, INHBB, KLRK1, LAG3, LGALS9, LILRA2, LILRB4, LY9, MDK, NOS2, PDCD1LG2, PGLYRP2, PLA2G10, PLA2G1B, PTPRC, SEMA7A, SLAMF1, SLAMF6, SPON2, TIGIT, TNFRSF14, TNFRSF1B, TNFRSF21, TNFRSF8, TRIM21, VSIG4, VSIR, XCL1 |
| T cell activation, differentiation, and migration | 6.23E-05 | 58 | ADA, B2M, CCL2, CD160, CD27, CD274, CD28, CD300A, CD4, CD46, CD48, CD6, CD7, CD74, CD80, CD83, CD8A, CLEC4A, CRTAM, CTSL, FCGR2B, FGL1, HAVCR2, HLA-DRA, ICAM1, IFNL1, IGFBP2, IL12RB1, IL18, IL1RL2, IL2RA, IL6ST, IL7, ITGAL, KLRK1, LAG3, LGALS9, LILRB4, LY9, MDK, PDCD1LG2, PTPRC, SIRPB1, SLAMF1, SLAMF6, SLAMF7, TGFBR2, TIGIT, TNFRSF14, TNFRSF1B, TNFRSF21, TNFRSF4, TNFRSF9, TNFSF13B, VCAM1, VSIG4, XCL1, VSIR |
| Regulation of MAPK cascade | 0.029 | 56 | ACE2, ADAM9, AIDA, ARHGEF5, BMPER, CCL11, CCL14, CCL15, CCL17, CCL2, CCL20, CCL23, CCL25, CCL4, CCL8, CD27, CD300A, CD4, CD40, CD74, CDH2, CX3CL1, EDA2R, EPHA2, F2R, FAS, FCGR2B, FCRL3, FLT4, GDF15, GH1, GPR37, HAVCR2, HGF, ICAM1, IGFBP3, IGFBP4, LGALS9, LILRB4, LTBR, MARCO, MYDGF, NOTCH1, PDGFRA, PLA2G1B, PTPRC, REN, RNF149, ROBO1, SEMA7A, SLAMF1, SMPD1, TNFRSF11A, TNFRSF19, VEGFA, XCL1 |
| Angiogenesis | 0.029 | 52 | ACVRL1, ADGRG1, AMOT, ANGPT2, B4GALT1, BMPER, BSG, CCL11, CCL2, CCN3, CD160, CD40, CDH5, CLEC14A, COL18A1, COL4A1, CX3CL1, CXCL10, CXCL13, CXCL8, DLL1, EPHA2, EPHB4, FLT4, GRN, HGF, HS6ST1, HSPG2, HYAL1, IL18, MDK, MYDGF, NOS3, NOTCH1, NOTCH3, NRCAM, NRP2, PDGFRA, PGF, PTPRM, ROBO1, RSPO3, SCG2, SMOC2, TGFBR2, THBS2, THBS4, TIE1, TNFRSF12A, TYMP, VEGFA, VEGFC |
| Mononuclear cell migration | 1.69E-05 | 37 | ARHGEF5, CCL11, CCL14, CCL15, CCL17, CCL2, CCL20, CCL23, CCL25, CCL27, CCL4, CCL8, CCN3, CRTAM, CSF1, CX3CL1, CXCL10, CXCL11, CXCL12, CXCL13, CXCL16, F11R, ICAM1, ITGAL, ITGB7, JAM2, KLRK1, LGALS9, LGMN, MDK, MSTN, NBL1, SLAMF1, SLAMF8, TNFRSF11A, TNFRSF14, XCL1 |
| Response to tumor necrosis factor | 4.34E-04 | 34 | ADAM9, ASAH1, CCL11, CCL14, CCL15, CCL17, CCL2, CCL20, CCL23, CCL25, CCL4, CCL8, CD14, CD40, CX3CL1, CXCL16, CXCL8, EDA2R, FAS, HYAL1, IL18BP, KRT18, SMPD1, TNFRSF11A, TNFRSF14, TNFRSF17, TNFRSF19, TNFRSF1A, TNFRSF1B, TNFRSF21, TNFRSF4, TNFSF13B, VCAM1, XCL1 |
| Granulocyte migration | 1.16E-04 | 32 | ADGRE2, BSG, CCL11, CCL14, CCL15, CCL17, CCL2, CCL20, CCL23, CCL25, CCL4, CCL8, CD300A, CD74, CSF1, CX3CL1, CXCL10, CXCL11, CXCL13, CXCL6, CXCL8, CXCL9, IL1R1, ITGB2, MDK, MSTN, PLA2G1B, SCG2, SLAMF1, SLAMF8, THBS4, XCL1 |
| Viral life cycle | 0.035 | 30 | ACE2, ATG16L1, AXL, BSG, BST2, CCL2, CCL8, CD28, CD4, CD46, CD74, CD80, CTSL, CXCL8, EPHA2, F11R, HAVCR1, ICAM1, ITGB7, LGALS9, NECTIN2, NOTCH1, P4HB, SCARB2, SIGLEC1, SLAMF1, SMPD1, TNFRSF14, TNFRSF4, TRIM21 |
| Regulation of ERK1/ERK2 cascade | 0.037 | 27 | BMPER, CCL11, CCL14, CCL15, CCL17, CCL2, CCL20, CCL23, CCL25, CCL4, CCL8, CD4, CD74, CX3CL1, F2R, FLT4, HAVCR2, ICAM1, LGALS9, MARCO, NOTCH1, PDGFRA, PTPRC, SEMA7A, SLAMF1, TNFRSF11A, XCL1 |
| Response to IL-1 | 4.03E-04 | 23 | CCL11, CCL14, CCL15, CCL17, CCL2, CCL20, CCL23, CCL25, CCL4, CCL8, CD38, CD40, CX3CL1, CXCL8, HYAL1, IL1R1, IL1RL2, INHBB, LGALS9, SMPD1, TNFRSF11A, XCL1, ZBP1 |
| αβ T cell activation and migration | 0.001 | 23 | ADA, CD160, CD274, CD28, CD300A, CD80, CD83, CLEC4A, CRTAM, CTSL, HLA-DRA, IL12RB1, IL18, IL2RA, LGALS9, LILRB4, LY9, PTPRC, SLAMF6, TGFBR2, TNFRSF14, VSIR, XCL1 |
| Interferon-γ production | 0.001 | 21 | AXL, BTN3A2, CD14, CD160, CD244, CD274, CRTAM, HAVCR2, IFNL1, IL12RB1, IL18, IL1R1, KLRK1, LGALS9, LILRB4, PDCD1LG2, PGLYRP2, SLAMF1, SLAMF6, VSIR, XCL1 |
| B cell activation | 0.007 | 18 | ADA, CD27, CD28, CD300A, CD38, CD40, CD74, FCGR2B, FCRL3, IGLC2, IL7, MZB1, PTPRC, SLAMF8, TNFRSF13B, TNFRSF21, TNFRSF4, TNFSF13B |
| IL-10 production | 0.007 | 14 | CD274, CD28, CD46, CD83, DLL1, FCGR2B, HGF, LGALS9, LILRB4, PDCD1LG2, TIGIT, TNFRSF21, XCL1, VSIR |
| Natural killer cell mediated cytotoxicity | 0.030 | 13 | CD160, CRTAM, HAVCR2, IL18, KLRD1, KLRK1, LAG3, LGALS9, NCR1, NECTIN2, SH2D1A, SLAMF6, SLAMF7 |
| T_reg_ cell differentiation | 0.020 | 8 | CD28, CD46, HLA-DRA, LAG3, LGALS9, LILRB4, MDK, VSIR |

^a ^Estimated using Fisher’s exact test, Gene Ontology Biological Processes reference database, and a threshold for significance of Benjamini-Hochberg false discovery rate (FDR)<0.05. 

MAPK=mitogen-activated protein kinases; ERK=extracellular signal-regulated kinases.

Previously published in Peterson TE, Hahn VS, Moaddel R, *et al.* Proteomic signature of HIV-associated subclinical left atrial remodeling and incident heart failure. *Nat Commun* **16**, 610 (2025). [https://doi.org/10.1038/s41467-025-55911-0](https://doi.org/10.1038/s41467-025-55911-0)

**SUPPLEMENTAL TABLE S3.** Individual proteins comprising the HIV-associated protein cluster (n=42), agnostically defined using weighted gene co-expression network analysis. 

| Protein | UniProt |
| --- | --- |
| ACVRL1 | P37023 |
| B2M | P61769 |
| BSG | P35613 |
| BTN2A1 | Q7KYR7 |
| CD46 | P15529 |
| CD93 | Q9NPY3 |
| CKAP4 | Q07065 |
| CLEC14A | Q86T13 |
| CST3 | P01034 |
| DLL1 | O00548 |
| DSC2 | Q02487 |
| EFNA4 | P52798 |
| EPHA2 | P29317 |
| EPHB4 | P54760 |
| EPHB6 | O15197 |
| ESAM | Q96AP7 |
| FSTL3 | O95633 |
| HAVCR2 | Q8TDQ0 |
| IFNGR1 | P15260 |
| IGFBP4 | P22692 |
| IL10RB | Q08334 |
| JAM2 | P57087 |
| LAYN | Q6UX15 |
| LTBR | P36941 |
| NBL1 | P41271 |
| NECTIN2 | Q92692 |
| NECTIN4 | Q96NY8 |
| NPDC1 | Q9NQX5 |
| PIK3IP1 | Q96FE7 |
| RELT | Q969Z4 |
| SCARB2 | Q14108 |
| SHISA5 | Q8N114 |
| TGFBR2 | P37173 |
| THBD | P07204 |
| TNFRSF10B | O14763 |
| TNFRSF11A | Q9Y6Q6 |
| TNFRSF14 | Q92956 |
| TNFRSF19 | Q9NS68 |
| TNFRSF1A | P19438 |
| TNFRSF1B | P20333 |
| TNFRSF21 | O75509 |
| VSIG4 | Q9Y279 |

Previously published in Peterson TE, *et al.* Proteomic signature of HIV-associated subclinical left atrial remodeling and incident heart failure. *Nat Commun* **16**, 610 (2025). 

**SUPPLEMENTAL TABLE S4.** Individual proteins in HIV-associated protein cluster defined using weighted gene co-expression network analysis corresponding to statistically over-represented biological processes. 

| Gene Ontology: Biological Process | Cluster Protein Count ^a^ | Measured Protein Count ^b^ | Enrichment *p*-value ^c^ | Enrichment FDR ^c^ | Proteins |
| --- | --- | --- | --- | --- | --- |
| cell-cell adhesion | 14 | 371 | 4.1E-04 | 0.035 | B2M, CD46, CD93, DSC2, ESAM, FSTL3, HAVCR2, JAM2, NECTIN2, NECTIN4, TGFBR2, TNFRSF14, TNFRSF21, VSIG4 |
| cytokine production | 12 | 293 | 5.8E-04 | 0.036 | B2M, BSG, BTN2A1, CD46, DLL1, EPHA2, HAVCR2, IFNGR1, TNFRSF14, TNFRSF1B, TNFRSF21, VSIG4 |
| regulation of T cell activation | 8 | 152 | 0.001 | 0.038 | B2M, CD46, HAVCR2, TGFBR2, TNFRSF14,              TNFRSF1B, TNFRSF21, VSIG4 |
| leukocyte cell-cell adhesion | 8 | 178 | 0.003 | 0.054 | B2M, CD46, HAVCR2, JAM2, TGFBR2,                              TNFRSF14, TNFRSF21, VSIG4 |
| regulation of T cell proliferation | 7 | 92 | 2.7E-04 | 0.029 | CD46, HAVCR2, TGFBR2, TNFRSF14,                             TNFRSF1B, TNFRSF21, VSIG4 |
| response to tumor necrosis factor | 6 | 85 | 0.001 | 0.038 | TNFRSF11A, TNFRSF14, TNFRSF19,                               TNFRSF1A, TNFRSF1B, TNFRSF21 |
| regulation of hemopoiesis | 6 | 129 | 0.010 | 0.095 | B2M, CD46, DLL1, FSTL3, TGFBR2, TNFRSF11A |
| ephrin receptor signaling pathway | 4 | 15 | 4.6E-05 | 0.019 | EFNA4, EPHA2, EPHB4, EPHB6 |
| regulation of BMP signaling pathway | 4 | 36 | 0.002 | 0.043 | ACVRL1, FSTL3, NBL1, MICOS10-NBL1 |
| macrophage activation | 4 | 51 | 0.006 | 0.073 | CD93, HAVCR2, IFNGR1, VSIG4 |
| heart morphogenesis | 4 | 54 | 0.007 | 0.080 | ACVRL1, DLL1, EPHB4, TGFBR2 |
| regulation of ECM organization | 3 | 8 | 1.5E-04 | 0.022 | CST3, TNFRSF1A, TNFRSF1B |
| tight junction assembly | 3 | 13 | 7.3E-04 | 0.037 | ACVRL1, EPHA2, ESAM |
| myeloid dendritic cell activation | 3 | 14 | 9.2E-04 | 0.038 | HAVCR2, LTBR, TGFBR2 |
| activin receptor signaling pathway | 3 | 18 | 0.002 | 0.043 | ACVRL1, FSTL3, TGFBR2 |
| interleukin-10 production | 3 | 30 | 0.009 | 0.090 | CD46, DLL1, TNFRSF21 |

Previously published in Peterson TE, Hahn VS, Moaddel R, *et al.* Proteomic signature of HIV-associated subclinical left atrial remodeling and incident heart failure. *Nat Commun* **16**, 610 (2025). [https://doi.org/10.1038/s41467-025-55911-0](https://doi.org/10.1038/s41467-025-55911-0)

^a ^Count of proteins within protein cluster of interest mapping to biological process. *N*=40 of 42 total cluster proteins were successfully mapped.

^b ^Count of measured Olink proteins mapping to biological process. *N*=2463 of 2594 total measured proteins were successfully mapped.

^c ^Estimated using Fisher’s exact test and GO: Biological Processes reference database; false discovery rate (FDR) applied Benjamini-Hochberg procedure

**SUPPLEMENTAL TABLE S5.** Cross-sectional associations between HIV-associated plasma protein abundances and elevated myocardial extracellular volume fraction among PWH and PWOH in the United States (*n*=342).

See Supplemental Excel file ‘Supplemental Tables.xlsx’

**SUPPLEMENTAL TABLE S6.** Pairwise Spearman's correlation coefficients between 39 individual proteins associated with positive HIV serostatus and elevated ECV with concordant directionality in SMASH (multivariable models, FDR<0.05).

See Supplemental Excel file ‘Supplemental Tables.xlsx’

**SUPPLEMENTAL TABLE S7.** Over-representation analysis of proteins cross-sectionally associated with HIV serostatus and elevated myocardial extracellular volume fraction, mapping to Gene Ontology: Biological Process annotations.

See Supplemental Excel file ‘Supplemental Tables.xlsx’

**SUPPLEMENTAL TABLE S8.** Percent difference in association between HIV serostatus and elevated myocardial extracellular volume fraction with adjustment for plasma abundance of 39 individual candidate protein contributors (*n*=342).

| Protein | PR for High ECV, PWH vs. PWOH (95% CI) | *p*-value | Reduction in HIV Association with Protein Adjustment, % |
| --- | --- | --- | --- |
| TFF3 | 1.12 (0.89 to 1.42) | 0.336 | 45.9 |
| CD27 | 1.12 (0.89 to 1.42) | 0.329 | 45.5 |
| PSAP | 1.13 (0.90 to 1.41) | 0.287 | 42.8 |
| REG4 | 1.13 (0.90 to 1.43) | 0.295 | 42.1 |
| SHISA5 | 1.15 (0.91 to 1.44) | 0.239 | 36.4 |
| FOLR2 | 1.15 (0.92 to 1.43) | 0.233 | 36.0 |
| GDF15 | 1.15 (0.92 to 1.45) | 0.226 | 34.1 |
| IGFBP4 | 1.16 (0.93 to 1.45) | 0.190 | 30.8 |
| TNFRSF10B | 1.16 (0.93 to 1.45) | 0.193 | 30.3 |
| NPC2 | 1.16 (0.93 to 1.45) | 0.187 | 30.3 |
| CD48 | 1.16 (0.93 to 1.46) | 0.197 | 30.0 |
| GM2A | 1.16 (0.93 to 1.46) | 0.193 | 29.8 |
| CKAP4 | 1.16 (0.93 to 1.46) | 0.191 | 29.7 |
| EDA2R | 1.17 (0.93 to 1.46) | 0.171 | 27.1 |
| EPHB4 | 1.17 (0.94 to 1.46) | 0.164 | 26.2 |
| TNFRSF1B | 1.17 (0.93 to 1.47) | 0.169 | 26.0 |
| OGN | 1.17 (0.94 to 1.47) | 0.164 | 25.8 |
| CPXM2 | 1.17 (0.94 to 1.46) | 0.156 | 25.5 |
| JAM2 | 1.17 (0.94 to 1.46) | 0.156 | 25.4 |
| HAVCR2 | 1.17 (0.94 to 1.47) | 0.157 | 24.7 |
| CRIM1 | 1.17 (0.94 to 1.47) | 0.155 | 24.7 |
| FGL1 | 1.17 (0.94 to 1.47) | 0.157 | 24.6 |
| CKB | 1.18 (0.94 to 1.47) | 0.150 | 23.9 |
| KRT19 | 1.18 (0.94 to 1.47) | 0.153 | 23.4 |
| WFDC2 | 1.18 (0.95 to 1.47) | 0.143 | 22.9 |
| TFPI2 | 1.18 (0.94 to 1.47) | 0.146 | 22.7 |
| IGFBP2 | 1.18 (0.95 to 1.47) | 0.142 | 22.2 |
| TNFRSF19 | 1.18 (0.95 to 1.47) | 0.139 | 22.0 |
| LAYN | 1.19 (0.95 to 1.48) | 0.132 | 20.3 |
| IGFBPL1 | 1.19 (0.95 to 1.49) | 0.126 | 18.7 |
| CXCL16 | 1.19 (0.95 to 1.49) | 0.127 | 18.6 |
| ASAH1 | 1.19 (0.95 to 1.48) | 0.123 | 18.6 |
| LY6D | 1.19 (0.96 to 1.48) | 0.118 | 18.0 |
| TNFRSF12A | 1.19 (0.95 to 1.49) | 0.121 | 17.5 |
| PIGR | 1.19 (0.96 to 1.49) | 0.119 | 17.4 |
| NCR3LG1 | 1.19 (0.96 to 1.49) | 0.116 | 16.8 |
| OCLN | 1.20 (0.96 to 1.49) | 0.116 | 16.5 |
| PLAUR | 1.20 (0.96 to 1.49) | 0.108 | 15.2 |
| COL4A1 | 1.20 (0.97 to 1.50) | 0.100 | 14.1 |

Prevalence ratio (PR) for high ECV (≥30% among women and ≥28% among men) estimated using modified Poisson regression, adjusting for age, sex, race, ethnicity, education, body mass index, systolic blood pressure, anti-hypertensive medication, dyslipidemia, diabetes, current hazardous alcohol use, cumulative pack-years of smoking in prior 5 years, stimulant use in prior 5 years, opioid use in prior 5 years, hepatitis C, estimated glomerular filtration rate, and indicated protein. ECV=myocardial extracellular volume fraction; SD=standard deviation; CI=confidence interval; FDR=false discovery rate (Benjamini-Hochberg).

**SUPPLEMENTAL TABLE S9.** Difference in association between plasma abundance of 39 individual proteins of interest and elevated myocardial extracellular volume fraction by HIV serostatus in SMASH (*n*=342).

| Protein | PR for High ECV per SD Increment in Plasma Protein, PWH (95% CI) | PR for High ECV per SD Increment in Plasma Protein, PWOH (95% CI) | Interaction *p*-value | Interaction FDR |
| --- | --- | --- | --- | --- |
| ASAH1 | 1.23 (1.08 to 1.39) | 1.03 (0.82 to 1.29) | 0.730 | 0.933 |
| CD27 | 1.16 (1.03 to 1.32) | 1.14 (0.91 to 1.42) | 0.548 | 0.933 |
| CD48 | 1.16 (1.02 to 1.31) | 1.10 (0.84 to 1.42) | 0.895 | 0.953 |
| CKAP4 | 1.15 (1.02 to 1.29) | 1.15 (0.91 to 1.44) | 0.664 | 0.933 |
| CKB | 1.10 (0.95 to 1.28) | 1.28 (1.05 to 1.57) | 0.194 | 0.933 |
| COL4A1 | 1.21 (1.07 to 1.36) | 1.09 (0.91 to 1.31) | 0.735 | 0.933 |
| CPXM2 | 1.17 (1.04 to 1.31) | 1.14 (0.92 to 1.42) | 0.928 | 0.953 |
| CRIM1 | 1.12 (0.99 to 1.27) | 1.19 (0.93 to 1.54) | 0.496 | 0.933 |
| CXCL16 | 1.15 (1.01 to 1.31) | 1.09 (0.86 to 1.38) | 0.575 | 0.933 |
| EDA2R | 1.17 (1.03 to 1.33) | 1.12 (0.86 to 1.46) | 0.741 | 0.933 |
| EPHB4 | 1.12 (1.00 to 1.26) | 1.14 (0.93 to 1.41) | 0.461 | 0.933 |
| FGL1 | 1.10 (0.96 to 1.26) | 1.34 (1.08 to 1.68) | 0.033 | 0.933 |
| FOLR2 | 1.15 (1.02 to 1.30) | 1.26 (1.01 to 1.56) | 0.384 | 0.933 |
| GDF15 | 1.15 (1.03 to 1.29) | 1.05 (0.84 to 1.31) | 0.886 | 0.953 |
| GM2A | 1.18 (1.03 to 1.35) | 1.12 (0.89 to 1.40) | 0.813 | 0.933 |
| HAVCR2 | 1.15 (1.02 to 1.30) | 1.13 (0.93 to 1.37) | 0.754 | 0.933 |
| IGFBP2 | 1.16 (1.00 to 1.35) | 1.26 (1.01 to 1.56) | 0.489 | 0.933 |
| IGFBP4 | 1.18 (1.06 to 1.32) | 1.18 (0.94 to 1.50) | 0.413 | 0.933 |
| IGFBPL1 | 1.12 (1.00 to 1.26) | 1.13 (0.94 to 1.36) | 0.552 | 0.933 |
| JAM2 | 1.27 (1.12 to 1.44) | 1.04 (0.83 to 1.30) | 0.164 | 0.933 |
| KRT19 | 1.08 (0.96 to 1.21) | 1.35 (1.05 to 1.74) | 0.089 | 0.933 |
| LAYN | 1.20 (1.07 to 1.34) | 1.12 (0.89 to 1.42) | 0.586 | 0.933 |
| LY6D | 1.18 (1.07 to 1.30) | 1.08 (0.88 to 1.33) | 0.595 | 0.933 |
| NCR3LG1 | 1.07 (0.95 to 1.21) | 1.20 (1.00 to 1.44) | 0.293 | 0.933 |
| NPC2 | 1.21 (1.06 to 1.39) | 1.17 (0.95 to 1.43) | 0.801 | 0.933 |
| OCLN | 1.12 (1.00 to 1.26) | 1.14 (0.91 to 1.42) | 0.676 | 0.933 |
| OGN | 1.17 (1.03 to 1.33) | 1.13 (0.90 to 1.42) | 0.981 | 0.981 |
| PIGR | 1.22 (1.05 to 1.41) | 1.01 (0.83 to 1.23) | 0.653 | 0.933 |
| PLAUR | 1.18 (1.03 to 1.34) | 1.05 (0.87 to 1.27) | 0.909 | 0.953 |
| PSAP | 1.25 (1.09 to 1.43) | 1.12 (0.91 to 1.38) | 0.720 | 0.933 |
| REG4 | 1.12 (0.98 to 1.28) | 1.22 (0.97 to 1.52) | 0.326 | 0.933 |
| SHISA5 | 1.15 (1.02 to 1.29) | 1.19 (0.93 to 1.51) | 0.606 | 0.933 |
| TFF3 | 1.16 (1.04 to 1.28) | 1.15 (0.89 to 1.47) | 0.690 | 0.933 |
| TFPI2 | 1.20 (1.08 to 1.34) | 0.97 (0.81 to 1.16) | 0.325 | 0.933 |
| TNFRSF10B | 1.17 (1.04 to 1.33) | 1.13 (0.92 to 1.38) | 0.769 | 0.933 |
| TNFRSF12A | 1.14 (1.03 to 1.27) | 1.04 (0.83 to 1.29) | 0.754 | 0.933 |
| TNFRSF19 | 1.16 (1.04 to 1.31) | 1.15 (0.91 to 1.44) | 0.700 | 0.933 |
| TNFRSF1B | 1.16 (1.04 to 1.30) | 1.01 (0.80 to 1.27) | 0.752 | 0.933 |
| WFDC2 | 1.13 (0.99 to 1.28) | 1.19 (0.99 to 1.43) | 0.164 | 0.933 |

Prevalence ratio (PR) for high ECV (≥30% among women and ≥28% among men) estimated using modified Poisson regression, adjusting for age, sex, race, ethnicity, education, body mass index, systolic blood pressure, anti-hypertensive medication, dyslipidemia, diabetes, current hazardous alcohol use, cumulative pack-years of smoking in prior 5 years, stimulant use in prior 5 years, opioid use in prior 5 years, hepatitis C, estimated glomerular filtration rate, and indicated protein. Interaction *p*-values and false discovery rates were estimated using an HIV×protein multiplicative interaction in models with the same covariates. 

PR=prevalence ratio; ECV=myocardial extracellular volume fraction; SD=standard deviation; CI=confidence interval; FDR=false discovery rate (Benjamini-Hochberg).

**SUPPLEMENTAL TABLE S10.** Sensitivity analysis: cross-sectional associations between plasma proteins of interest and elevated myocardial extracellular volume fraction in SMASH, excluding participants with prior myocardial infarction and/or evidence of ischemic scarring on CMR (*n*=323)

| Protein | Original (no exclusions, n=342) |  | Excluding Participants with prior MI and/or Ischemic Scar Pattern (n=323) |  |
| --- | --- | --- | --- | --- |
|  | PR (95% CI) | *p*-value | PR (95% CI) | *p*-value |
| ASAH1 | 1.16 (1.04 to 1.30) | 0.0080 | 1.16 (1.03 to 1.30) | 0.0163 |
| CD27 | 1.15 (1.03 to 1.28) | 0.0100 | 1.14 (1.02 to 1.28) | 0.0267 |
| CD48 | 1.14 (1.03 to 1.28) | 0.0157 | 1.13 (1.01 to 1.27) | 0.0355 |
| CKAP4 | 1.14 (1.03 to 1.27) | 0.0123 | 1.13 (1.02 to 1.27) | 0.0239 |
| CKB | 1.16 (1.03 to 1.31) | 0.0117 | 1.16 (1.03 to 1.31) | 0.0177 |
| COL4A1 | 1.18 (1.07 to 1.30) | 0.0013 | 1.19 (1.07 to 1.32) | 0.0015 |
| CPXM2 | 1.16 (1.05 to 1.28) | 0.0040 | 1.14 (1.03 to 1.27) | 0.0116 |
| CRIM1 | 1.15 (1.03 to 1.28) | 0.0120 | 1.15 (1.02 to 1.29) | 0.0182 |
| CXCL16 | 1.13 (1.01 to 1.27) | 0.0285 | 1.12 (1.00 to 1.26) | 0.0484 |
| EDA2R | 1.16 (1.03 to 1.30) | 0.0119 | 1.16 (1.03 to 1.31) | 0.0170 |
| EPHB4 | 1.14 (1.03 to 1.26) | 0.0084 | 1.14 (1.02 to 1.26) | 0.0176 |
| FGL1 | 1.16 (1.03 to 1.31) | 0.0124 | 1.14 (1.01 to 1.29) | 0.0379 |
| FOLR2 | 1.17 (1.06 to 1.30) | 0.0028 | 1.17 (1.04 to 1.30) | 0.0080 |
| GDF15 | 1.13 (1.02 to 1.24) | 0.0177 | 1.10 (1.00 to 1.23) | 0.0610 |
| GM2A | 1.16 (1.03 to 1.30) | 0.0113 | 1.16 (1.02 to 1.31) | 0.0198 |
| HAVCR2 | 1.14 (1.03 to 1.27) | 0.0092 | 1.14 (1.03 to 1.27) | 0.0134 |
| IGFBP2 | 1.19 (1.05 to 1.35) | 0.0061 | 1.18 (1.04 to 1.34) | 0.0117 |
| IGFBP4 | 1.19 (1.07 to 1.31) | 0.0008 | 1.19 (1.07 to 1.32) | 0.0018 |
| IGFBPL1 | 1.13 (1.03 to 1.25) | 0.0089 | 1.11 (1.00 to 1.23) | 0.0402 |
| JAM2 | 1.18 (1.07 to 1.31) | 0.0011 | 1.17 (1.06 to 1.30) | 0.0025 |
| KRT19 | 1.13 (1.01 to 1.25) | 0.0295 | 1.15 (1.03 to 1.28) | 0.0144 |
| LAYN | 1.16 (1.05 to 1.28) | 0.0034 | 1.16 (1.05 to 1.29) | 0.0048 |
| LY6D | 1.15 (1.05 to 1.27) | 0.0028 | 1.17 (1.06 to 1.30) | 0.0020 |
| NCR3LG1 | 1.12 (1.02 to 1.23) | 0.0198 | 1.12 (1.01 to 1.24) | 0.0293 |
| NPC2 | 1.21 (1.08 to 1.35) | 0.0009 | 1.19 (1.06 to 1.34) | 0.0027 |
| OCLN | 1.13 (1.02 to 1.25) | 0.0167 | 1.13 (1.02 to 1.26) | 0.0223 |
| OGN | 1.16 (1.04 to 1.30) | 0.0096 | 1.14 (1.02 to 1.28) | 0.0265 |
| PIGR | 1.14 (1.02 to 1.28) | 0.0227 | 1.12 (0.99 to 1.27) | 0.0700 |
| PLAUR | 1.13 (1.01 to 1.27) | 0.0322 | 1.13 (1.00 to 1.27) | 0.0444 |
| PSAP | 1.21 (1.08 to 1.35) | 0.0010 | 1.21 (1.08 to 1.37) | 0.0011 |
| REG4 | 1.15 (1.03 to 1.29) | 0.0172 | 1.13 (1.00 to 1.27) | 0.0478 |
| SHISA5 | 1.16 (1.05 to 1.30) | 0.0054 | 1.15 (1.03 to 1.29) | 0.0131 |
| TFF3 | 1.15 (1.04 to 1.27) | 0.0054 | 1.14 (1.02 to 1.27) | 0.0228 |
| TFPI2 | 1.12 (1.03 to 1.23) | 0.0122 | 1.12 (1.01 to 1.24) | 0.0360 |
| TNFRSF10B | 1.17 (1.05 to 1.30) | 0.0035 | 1.18 (1.05 to 1.32) | 0.0055 |
| TNFRSF12A | 1.12 (1.02 to 1.23) | 0.0129 | 1.11 (1.01 to 1.22) | 0.0268 |
| TNFRSF19 | 1.16 (1.05 to 1.29) | 0.0053 | 1.17 (1.04 to 1.30) | 0.0068 |
| TNFRSF1B | 1.13 (1.02 to 1.25) | 0.0168 | 1.11 (1.00 to 1.23) | 0.0585 |
| WFDC2 | 1.16 (1.05 to 1.28) | 0.0047 | 1.14 (1.02 to 1.27) | 0.0166 |

Prevalence ratio (PR) for high ECV (≥30% among women and ≥28% among men) estimated per SD increment in indicated protein plasma level using modified Poisson regression, adjusting for age, sex, race, ethnicity, education, body mass index, systolic blood pressure, anti-hypertensive medication, dyslipidemia, diabetes, HIV, hepatitis C, current hazardous alcohol use, cumulative pack-years of smoking in prior 5 years, stimulant use in prior 5 years, opioid use in prior 5 years, and estimated glomerular filtration rate. 

PR=prevalence ratio; ECV=myocardial extracellular volume fraction; SD=standard deviation; CI=confidence interval; FDR=false discovery rate (Benjamini-Hochberg).

**SUPPLEMENTAL TABLE S11.** Sensitivity analysis: cross-sectional associations between plasma proteins of interest and T2 time among a subset of SMASH participants with measured T2 (*n*=81).

| Protein | Mean Difference in T2 time (ms) per SD Increment in Plasma Protein (95% CI) | *p*-value | FDR |
| --- | --- | --- | --- |
| ASAH1 | -0.23 (-1.55 to 1.10) | 0.737 | 0.959 |
| CD27 | 0.06 (-0.96 to 1.08) | 0.909 | 0.992 |
| CD48 | -0.59 (-1.67 to 0.49) | 0.287 | 0.959 |
| CKAP4 | -0.47 (-1.52 to 0.58) | 0.383 | 0.959 |
| CKB | 0.06 (-1.01 to 1.12) | 0.916 | 0.992 |
| COL4A1 | 0.31 (-0.81 to 1.42) | 0.590 | 0.959 |
| CPXM2 | -0.07 (-0.88 to 0.73) | 0.858 | 0.992 |
| CRIM1 | -0.20 (-1.10 to 0.70) | 0.666 | 0.959 |
| CXCL16 | -0.76 (-1.70 to 0.18) | 0.113 | 0.917 |
| EDA2R | -0.17 (-1.02 to 0.69) | 0.704 | 0.959 |
| EPHB4 | -0.19 (-1.25 to 0.88) | 0.734 | 0.959 |
| FGL1 | -0.26 (-1.31 to 0.78) | 0.621 | 0.959 |
| FOLR2 | 0.16 (-0.71 to 1.02) | 0.721 | 0.959 |
| GDF15 | -1.32 (-2.40 to -0.24) | 0.017 | 0.331 |
| GM2A | 0.24 (-0.57 to 1.06) | 0.559 | 0.959 |
| HAVCR2 | -0.53 (-1.64 to 0.58) | 0.350 | 0.959 |
| IGFBP2 | -0.03 (-1.07 to 1.00) | 0.952 | 0.995 |
| IGFBP4 | 0.00 (-0.94 to 0.94) | 1.000 | 1.000 |
| IGFBPL1 | 0.57 (-0.34 to 1.48) | 0.221 | 0.959 |
| JAM2 | 1.03 (0.21 to 1.85) | 0.014 | 0.331 |
| KRT19 | -0.62 (-1.68 to 0.43) | 0.248 | 0.959 |
| LAYN | -0.12 (-0.91 to 0.67) | 0.768 | 0.966 |
| LY6D | -0.69 (-1.62 to 0.23) | 0.141 | 0.917 |
| NCR3LG1 | -0.51 (-1.74 to 0.72) | 0.419 | 0.959 |
| NPC2 | 0.30 (-0.66 to 1.27) | 0.537 | 0.959 |
| OCLN | -0.34 (-1.42 to 0.74) | 0.536 | 0.959 |
| OGN | 0.49 (-0.35 to 1.32) | 0.255 | 0.959 |
| PIGR | -1.17 (-2.36 to 0.02) | 0.054 | 0.706 |
| PLAUR | -0.44 (-1.65 to 0.78) | 0.483 | 0.959 |
| PSAP | 0.10 (-0.79 to 1.00) | 0.820 | 0.992 |
| REG4 | 0.17 (-0.61 to 0.94) | 0.676 | 0.959 |
| SHISA5 | -0.18 (-1.13 to 0.76) | 0.704 | 0.959 |
| TFF3 | -0.02 (-0.87 to 0.84) | 0.970 | 0.995 |
| TFPI2 | -0.06 (-0.87 to 0.74) | 0.875 | 0.992 |
| TNFRSF10B | -0.80 (-1.82 to 0.22) | 0.124 | 0.917 |
| TNFRSF12A | -0.44 (-1.49 to 0.60) | 0.407 | 0.959 |
| TNFRSF19 | 0.17 (-0.62 to 0.96) | 0.674 | 0.959 |
| TNFRSF1B | -0.42 (-1.35 to 0.51) | 0.373 | 0.959 |
| WFDC2 | -0.24 (-1.06 to 0.57) | 0.559 | 0.959 |

Mean difference in T2 time (ms) estimated per SD increment in indicated protein plasma level using linear regression with robust variance, adjusting for age, sex, race, ethnicity, education, body mass index, systolic blood pressure, anti-hypertensive medication, dyslipidemia, diabetes, HIV, hepatitis C, current hazardous alcohol use, cumulative pack-years of smoking in prior 5 years, stimulant use in prior 5 years, opioid use in prior 5 years, and estimated glomerular filtration rate. 

PR=prevalence ratio; SD=standard deviation; CI=confidence interval; FDR=false discovery rate (Benjamini-Hochberg).

**SUPPLEMENTAL TABLE S12.** MESA participant characteristics (2010-2012) by analysis.

| CHARACTERISTIC | Median [IQR] or % (n) |  |
| --- | --- | --- |
|  | Cross-Sectional ECV Analysis (*n*=522) | Incident Event Analysis (*n*=3223) |
| *Demographics* |  |  |
| Age, years | 66 [60, 74] | 68 [61, 76] |
| Female sex at birth | 251 (48%) | 1693 (52%) |
| Race and ethnicity | .. | .. |
| Black, non-Hispanic | 120 (23%) | 733 (23%) |
| White, non-Hispanic | 326 (62%) | 1355 (42%) |
| Hispanic | 76 (15%) | 737 (23%) |
| Chinese | 0 (0%) | 398 (12%) |
| Education level ≥ high school | 484 (93%) | 2796 (87%) |
| *Clinical Factors* |  |  |
| Smoking status | .. | .. |
| Current | 48 (9%) | 236 (7%) |
| Former | 254 (49%) | 1504 (47%) |
| Never | 220 (42%) | 1483 (46%) |
| Pack-years of smoking | 0.1 [0.0, 15.5] | 0.0 [0.0, 13.0] |
| Current alcohol use | 295 (57%) | 1465 (46%) |
| Heavy alcohol use^ a^ | 56 (11%) | 271 (8%) |
| Body mass index, kg/m^2^ | 28.5 [25.4, 32.2] | 27.8 [24.6, 31.5] |
| Hypertension^ b^ | 305 (58%) | 1808 (56%) |
| Systolic blood pressure, mmHg | 119 [109, 133] | 119 [109, 136] |
| Diastolic blood pressure, mmHg | 69 [62, 75] | 68 [62, 75] |
| Blood pressure-lowering medication use | 277 (53%) | 1666 (52%) |
| Dyslipidemia^ c^ | 392 (75%) | 2392 (74%) |
| Total cholesterol, mg/dL | 176 [154, 203] | 182 [158, 208] |
| HDL-cholesterol, mg/dL | 51 [42, 62] | 53 [44, 64] |
| Lipid-lowering medication use | 223 (43%) | 1235 (38%) |
| Diabetes^ d^ | 92 (18%) | 589 (18%) |
| eGFR, CKD-EPI, mL/min/1.73m^2^ | 71 [62, 81] | 70 [59, 80] |
| *Cardiovascular Magnetic Resonance* |  |  |
| LV ejection fraction, % | 62.2 [57.0, 66.6] | .. |
| LV ejection fraction <50% | 24 (5%) | .. |
| LV mass indexed by BSA, mg/m^2^ | 64.6 [57.4, 74.0] | .. |
| LV end-diastolic volume indexed by BSA, mL/m^2^ | 63.2 [55.6, 72.7] | .. |
| LA volume indexed by BSA, mL/m^2^ | 35.0 [28.4, 42.6] | .. |
| Late gadolinium enhancement | 52 (10%) | .. |
| Extracellular volume fraction, % | 26.7 [24.6, 28.8] | .. |
| Elevated extracellular volume fraction^ e^ | 105 (20%) | .. |
| T1 time, ms | 975 [945, 1002] | .. |

^a ^Heavy alcohol use defined as ≥15 drinks per week among men and ≥8 drinks per week among women.

^b ^Hypertension is defined as use of antihypertensive medications or systolic blood pressure≥140 mmHg or diastolic blood pressure ≥90 mmHg.

^c ^Dyslipidemia is defined as use of lipid-lowering medications or fasting total cholesterol level ≥200 mg/dL or low-density lipoprotein cholesterol level ≥130 mg/dL or high-density lipoprotein cholesterol level <40 mg/dL or serum triglyceride level ≥150 mg/dL. 

^d ^Diabetes is defined as use of hypoglycemic medication or fasting serum glucose levels ≥126 mg/dL. 

^e^ Elevated extracellular volume fraction defined as ≥30% among women and ≥28% among men. 

IQR=interquartile range, reported as (25th, 75th) percentiles; LV=left ventricular; LA=left atrial; BSA=body surface area; HDL=high-density lipoprotein; eGFR=estimated glomerular filtration rate.

**SUPPLEMENTAL TABLE S13.** Cross-sectional associations between proteins of interest and elevated myocardial extracellular volume fraction in the Multi-Ethnic Study of Atherosclerosis (*n*=522)

| Protein | Model 1 |  |  | Model 2 |  |  |
| --- | --- | --- | --- | --- | --- | --- |
|  | Prevalence Ratio of High ECV per SD Increment in Plasma Protein (95% CI) | *p*-value | FDR | Prevalence Ratio of High ECV per SD Increment in Plasma Protein (95% CI) | *p*-value | FDR |
| ASAH1 | 0.85 (0.70 to 1.02) | 0.072 | 0.187 | 0.84 (0.71 to 1.01) | 0.060 | 0.181 |
| CD27 | 1.23 (1.03 to 1.46) | 0.022 | 0.084 | 1.21 (1.02 to 1.45) | 0.032 | 0.143 |
| CD48 | 1.18 (1.00 to 1.39) | 0.044 | 0.156 | 1.19 (1.02 to 1.40) | 0.030 | 0.143 |
| CKAP4 | 1.07 (0.90 to 1.29) | 0.441 | 0.567 | 1.03 (0.85 to 1.24) | 0.783 | 0.925 |
| CKB | 1.26 (1.04 to 1.52) | 0.016 | 0.076 | 1.23 (1.00 to 1.51) | 0.054 | 0.175 |
| COL4A1 | 1.09 (0.93 to 1.29) | 0.276 | 0.399 | 1.07 (0.90 to 1.28) | 0.438 | 0.760 |
| CPXM2 | 0.84 (0.70 to 1.02) | 0.081 | 0.187 | 0.83 (0.69 to 1.00) | 0.049 | 0.175 |
| CRIM1 | 1.22 (1.04 to 1.43) | 0.014 | 0.076 | 1.24 (1.05 to 1.45) | 0.010 | 0.122 |
| CXCL16 | 1.07 (0.90 to 1.27) | 0.465 | 0.567 | 1.02 (0.85 to 1.21) | 0.863 | 0.960 |
| EDA2R | 1.05 (0.85 to 1.30) | 0.648 | 0.737 | 0.99 (0.81 to 1.22) | 0.960 | 0.960 |
| EPHB4 | 1.12 (0.95 to 1.32) | 0.192 | 0.324 | 1.08 (0.92 to 1.27) | 0.345 | 0.673 |
| FGL1 | 1.13 (0.91 to 1.39) | 0.269 | 0.399 | 1.07 (0.88 to 1.31) | 0.499 | 0.760 |
| FOLR2 | **1.27 (1.08 to 1.49)** | **0.004** | **0.048** | **1.31 (1.11 to 1.55)** | **0.002** | **0.036** |
| GDF15 | 1.18 (1.00 to 1.41) | 0.057 | 0.185 | 1.07 (0.87 to 1.32) | 0.506 | 0.760 |
| GM2A | 1.13 (0.94 to 1.37) | 0.199 | 0.324 | 1.07 (0.88 to 1.31) | 0.468 | 0.760 |
| HAVCR2 | 1.14 (0.96 to 1.35) | 0.142 | 0.276 | 1.12 (0.93 to 1.34) | 0.223 | 0.512 |
| IGFBP2 | 1.27 (1.04 to 1.55) | 0.018 | 0.078 | 1.25 (1.01 to 1.56) | 0.044 | 0.173 |
| IGFBP4 | 1.19 (0.98 to 1.46) | 0.082 | 0.187 | 1.16 (0.94 to 1.43) | 0.172 | 0.420 |
| IGFBPL1 | 1.03 (0.86 to 1.24) | 0.721 | 0.781 | 1.01 (0.85 to 1.20) | 0.920 | 0.960 |
| JAM2 | 1.07 (0.90 to 1.27) | 0.452 | 0.567 | 1.04 (0.88 to 1.24) | 0.629 | 0.846 |
| KRT19 | 1.23 (1.05 to 1.44) | 0.011 | 0.074 | 1.18 (0.99 to 1.40) | 0.068 | 0.189 |
| LAYN | 1.10 (0.89 to 1.35) | 0.384 | 0.517 | 1.03 (0.84 to 1.27) | 0.782 | 0.925 |
| LY6D | 1.11 (0.92 to 1.34) | 0.272 | 0.399 | 1.12 (0.92 to 1.36) | 0.256 | 0.530 |
| NCR3LG1 | 1.21 (0.98 to 1.50) | 0.074 | 0.187 | 1.18 (0.94 to 1.48) | 0.145 | 0.377 |
| NPC2 | 1.11 (0.90 to 1.36) | 0.325 | 0.453 | 1.04 (0.85 to 1.29) | 0.682 | 0.887 |
| OCLN | 1.04 (0.87 to 1.24) | 0.661 | 0.737 | 0.99 (0.84 to 1.18) | 0.947 | 0.960 |
| OGN | 1.03 (0.83 to 1.29) | 0.770 | 0.790 | 1.02 (0.81 to 1.27) | 0.895 | 0.960 |
| PIGR | **1.33 (1.16 to 1.54)** | **5.97E-05** | **0.002** | **1.31 (1.10 to 1.55)** | **0.002** | **0.036** |
| PLAUR | 1.19 (0.98 to 1.44) | 0.081 | 0.187 | 1.13 (0.91 to 1.40) | 0.258 | 0.530 |
| PSAP | 0.85 (0.70 to 1.03) | 0.105 | 0.227 | 0.78 (0.64 to 0.95) | 0.013 | 0.122 |
| REG4 | 1.15 (0.97 to 1.38) | 0.115 | 0.236 | 1.06 (0.88 to 1.28) | 0.536 | 0.767 |
| SHISA5 | 1.15 (0.93 to 1.42) | 0.193 | 0.324 | 1.10 (0.88 to 1.37) | 0.406 | 0.754 |
| TFF3 | 1.21 (1.05 to 1.39) | 0.009 | 0.067 | 1.18 (1.03 to 1.36) | 0.019 | 0.122 |
| TFPI2 | 1.06 (0.89 to 1.27) | 0.512 | 0.605 | 1.03 (0.86 to 1.24) | 0.726 | 0.914 |
| TNFRSF10B | 1.14 (0.94 to 1.38) | 0.172 | 0.319 | 1.06 (0.87 to 1.31) | 0.551 | 0.767 |
| TNFRSF12A | 0.98 (0.81 to 1.19) | 0.844 | 0.844 | 0.93 (0.76 to 1.13) | 0.452 | 0.760 |
| TNFRSF19 | 1.03 (0.84 to 1.27) | 0.753 | 0.790 | 0.98 (0.80 to 1.22) | 0.886 | 0.960 |
| TNFRSF1B | 1.22 (1.06 to 1.40) | 0.007 | 0.067 | 1.21 (1.03 to 1.41) | 0.018 | 0.122 |
| WFDC2 | 1.34 (1.12 to 1.60) | 0.001 | 0.028 | 1.24 (1.02 to 1.52) | 0.033 | 0.143 |

Prevalence ratio for high ECV (≥30% among women and ≥28% among men) estimated using modified Poisson regression. Model 1 adjusts for study site, age, sex, race, ethnicity, and estimated glomerular filtration rate. Model 2 further adjusts for education, body mass index, systolic blood pressure, anti-hypertensive medication, hyperlipidemia, diabetes, smoking, and heavy alcohol use. 

ECV=myocardial extracellular volume fraction; SD=standard deviation; CI=confidence interval; FDR=false discovery rate (Benjamini-Hochberg). 

**Bold** indicates statistically significant following multiple testing correction (FDR<0.05).

**SUPPLEMENTAL TABLE S14.** Association between proteins of interest and time to incident clinical heart failure in the Multi-Ethnic Study of Atherosclerosis (*n*=118 incident cases among 3223 participants)

| Protein | Model 1 |  |  | Model 2 |  |  |
| --- | --- | --- | --- | --- | --- | --- |
|  | HR per SD Increment in Plasma Protein (95% CI) | *p*-value | FDR | HR per SD Increment in Plasma Protein (95% CI) | *p*-value | FDR |
| ASAH1 | 1.12 (0.94 to 1.34) | 0.196 | 0.207 | 1.08 (0.90 to 1.29) | 0.425 | 0.425 |
| CD27 | **1.68 (1.39 to 2.03)** | **6.35E-08** | **2.48E-07** | **1.66 (1.36 to 2.02)** | **5.33E-07** | **4.16E-06** |
| CD48 | **1.38 (1.16 to 1.65)** | **3.77E-04** | **0.001** | **1.35 (1.13 to 1.62)** | **0.001** | **0.002** |
| CKAP4 | **1.52 (1.25 to 1.84)** | **3.27E-05** | **6.45E-05** | **1.42 (1.16 to 1.75)** | **8.30E-04** | **0.001** |
| CKB | 1.18 (0.95 to 1.47) | 0.124 | 0.139 | **1.33 (1.06 to 1.66)** | **0.014** | **0.018** |
| COL4A1 | 1.11 (0.92 to 1.34) | 0.273 | 0.273 | 1.16 (0.96 to 1.41) | 0.117 | 0.124 |
| CPXM2 | **1.37 (1.13 to 1.66)** | **0.001** | **0.002** | **1.30 (1.06 to 1.59)** | **0.010** | **0.013** |
| CRIM1 | **1.45 (1.20 to 1.74)** | **1.00E-04** | **1.86E-04** | **1.40 (1.16 to 1.68)** | **4.03E-04** | **7.85E-04** |
| CXCL16 | **1.36 (1.12 to 1.67)** | **0.002** | **0.003** | **1.28 (1.05 to 1.57)** | **0.016** | **0.019** |
| EDA2R | **1.78 (1.41 to 2.26)** | **1.46E-06** | **4.07E-06** | **1.68 (1.31 to 2.15)** | **4.26E-05** | **1.11E-04** |
| EPHB4 | **1.38 (1.12 to 1.69)** | **0.002** | **0.003** | **1.32 (1.07 to 1.63)** | **0.008** | **0.012** |
| FGL1 | **1.37 (1.14 to 1.65)** | **0.001** | **0.001** | **1.36 (1.12 to 1.65)** | **0.002** | **0.003** |
| FOLR2 | **1.30 (1.07 to 1.57)** | **0.007** | **0.008** | **1.25 (1.03 to 1.51)** | **0.024** | **0.028** |
| GDF15 | **1.68 (1.40 to 2.01)** | **2.33E-08** | **1.30E-07** | **1.57 (1.28 to 1.93)** | **1.93E-05** | **5.80E-05** |
| GM2A | **1.38 (1.16 to 1.63)** | **1.94E-04** | **3.45E-04** | **1.28 (1.08 to 1.53)** | **0.005** | **0.008** |
| HAVCR2 | **1.44 (1.18 to 1.77)** | **4.44E-04** | **0.001** | **1.34 (1.09 to 1.65)** | **0.006** | **0.009** |
| IGFBP2 | 1.14 (0.92 to 1.43) | 0.235 | 0.241 | **1.30 (1.03 to 1.65)** | **0.027** | **0.030** |
| IGFBP4 | 2.07 (1.67 to 2.57) | **3.03E-11** | **1.18E-09** | **1.89 (1.51 to 2.38)** | **4.33E-08** | **8.43E-07** |
| IGFBPL1 | 1.39 (1.15 to 1.68) | **0.001** | **0.001** | **1.29 (1.06 to 1.57)** | **0.011** | **0.014** |
| JAM2 | 1.60 (1.29 to 1.97) | **1.22E-05** | **2.81E-05** | **1.58 (1.27 to 1.96)** | **4.76E-05** | **1.16E-04** |
| KRT19 | 1.60 (1.34 to 1.93) | **4E-07** | **1.2E-06** | **1.55 (1.28 to 1.87)** | **5.32E-06** | **1.73E-05** |
| LAYN | 1.85 (1.48 to 2.31) | **5.67E-08** | **2.45E-07** | **1.85 (1.48 to 2.33)** | **1.18E-07** | **1.53E-06** |
| LY6D | **1.24 (1.01 to 1.53)** | **0.041** | **0.049** | 1.19 (0.96 to 1.48) | 0.104 | 0.112 |
| NCR3LG1 | 1.21 (0.98 to 1.48) | 0.071 | 0.082 | 1.20 (0.97 to 1.47) | 0.086 | 0.096 |
| NPC2 | **1.70 (1.42 to 2.02)** | **3.71E-09** | **2.90E-08** | **1.58 (1.31 to 1.91)** | **1.81E-06** | **7.82E-06** |
| OCLN | **1.62 (1.37 to 1.91)** | **8.40E-09** | **5.46E-08** | **1.54 (1.30 to 1.83)** | **8.34E-07** | **4.65E-06** |
| OGN | **1.75 (1.38 to 2.21)** | **3.71E-06** | **9.04E-06** | **1.61 (1.26 to 2.07)** | **1.36E-04** | **3.02E-04** |
| PIGR | **1.51 (1.27 to 1.78)** | **2.27E-06** | **5.91E-06** | **1.46 (1.22 to 1.75)** | **3.72E-05** | **1.04E-04** |
| PLAUR | **1.52 (1.25 to 1.84)** | **2.50E-05** | **5.41E-05** | **1.43 (1.16 to 1.75)** | **6.64E-04** | **0.001** |
| PSAP | **1.35 (1.13 to 1.60)** | **0.001** | **0.001** | **1.28 (1.07 to 1.54)** | **0.006** | **0.009** |
| REG4 | **1.63 (1.35 to 1.96)** | **3.41E-07** | **1.11E-06** | **1.57 (1.30 to 1.90)** | **3.35E-06** | **1.19E-05** |
| SHISA5 | **1.73 (1.43 to 2.10)** | **2.89E-08** | **1.41E-07** | **1.63 (1.33 to 2.00)** | **3.34E-06** | **1.19E-05** |
| TFF3 | **1.32 (1.14 to 1.54)** | **3.25E-04** | **5.51E-04** | **1.34 (1.14 to 1.57)** | **3.67E-04** | **7.53E-04** |
| TFPI2 | 1.15 (0.95 to 1.40) | 0.154 | 0.167 | 1.15 (0.95 to 1.41) | 0.155 | 0.159 |
| TNFRSF10B | **1.84 (1.51 to 2.25)** | **1.59E-09** | **2.06E-08** | **1.70 (1.38 to 2.10)** | **6.98E-07** | **4.54E-06** |
| TNFRSF12A | **1.86 (1.52 to 2.28)** | **2.74E-09** | **2.67E-08** | **1.77 (1.43 to 2.19)** | **1.73E-07** | **1.68E-06** |
| TNFRSF19 | **1.67 (1.31 to 2.12)** | **3.31E-05** | **6.45E-05** | **1.62 (1.26 to 2.07)** | **1.40E-04** | **3.02E-04** |
| TNFRSF1B | **1.61 (1.35 to 1.92)** | **1.03E-07** | **3.66E-07** | **1.59 (1.32 to 1.92)** | **1.51E-06** | **7.37E-06** |
| WFDC2 | **1.90 (1.55 to 2.33)** | **7.44E-10** | **1.45E-08** | **1.84 (1.48 to 2.28)** | **2.98E-08** | **8.43E-07** |

Estimated using Cox proportional hazards. Model 1 adjusts for study site, age, sex, race, ethnicity, and estimated glomerular filtration rate. Model 2 further adjusts for education, body mass index, systolic blood pressure, anti-hypertensive medication, dyslipidemia, diabetes, smoking, and heavy alcohol use. N=118 incident adjudicated clinical heart failure events occurred over a median [interquartile range] follow-up period of 9.8 [1.0] years.

HR=hazard ratio; SD=standard deviation; CI=confidence interval; FDR=false discovery rate (Benjamini-Hochberg).

**Bold** indicates statistically significant following multiple testing correction (FDR<0.05).

**SUPPLEMENTAL TABLE S15.** Association between proteins of interest and time to incident clinical heart failure with preserved ejection fraction (HFpEF) and reduced ejection fraction (HFrEF) in the Multi-Ethnic Study of Atherosclerosis (*n*=3211)

| Protein | HFpEF (*n=*53 incident cases) |  |  | HFrEF (*n=*40 incident cases) |  |  |
| --- | --- | --- | --- | --- | --- | --- |
|  | HR per SD Increment in Plasma Protein (95% CI) | *p*-value | FDR | HR per SD Increment in Plasma Protein (95% CI) | *p*-value | FDR |
| ASAH1 | 1.14 (0.88 to 1.48) | 0.326 | 0.343 | 1.04 (0.75 to 1.43) | 0.822 | 0.891 |
| CD27 | **1.73 (1.29 to 2.34)** | **2.95E-04** | **0.001** | 1.34 (0.95 to 1.89) | 0.100 | 0.162 |
| CD48 | **1.40 (1.06 to 1.84)** | **0.017** | **0.026** | 1.37 (1.02 to 1.86) | 0.039 | 0.129 |
| CKAP4 | 1.37 (1.00 to 1.89) | 0.050 | 0.059 | 1.37 (0.97 to 1.93) | 0.070 | 0.140 |
| CKB | **1.48 (1.06 to 2.06)** | **0.021** | **0.030** | 1.14 (0.77 to 1.69) | 0.505 | 0.608 |
| COL4A1 | 1.15 (0.86 to 1.53) | 0.352 | 0.362 | 1.30 (0.95 to 1.78) | 0.107 | 0.166 |
| CPXM2 | **1.42 (1.07 to 1.89)** | **0.014** | **0.026** | 1.13 (0.79 to 1.62) | 0.506 | 0.608 |
| CRIM1 | **1.38 (1.04 to 1.83)** | **0.024** | **0.033** | 1.41 (1.02 to 1.94) | 0.036 | 0.129 |
| CXCL16 | 1.21 (0.90 to 1.64) | 0.212 | 0.230 | 1.37 (0.96 to 1.96) | 0.079 | 0.140 |
| EDA2R | **1.56 (1.08 to 2.24)** | **0.017** | **0.026** | **1.75 (1.16 to 2.63)** | **0.007** | **0.049** |
| EPHB4 | **1.38 (1.01 to 1.89)** | **0.041** | **0.049** | 1.35 (0.95 to 1.91) | 0.094 | 0.159 |
| FGL1 | **1.82 (1.37 to 2.42)** | **4.23E-05** | **2.75E-04** | 1.13 (0.83 to 1.55) | 0.441 | 0.573 |
| FOLR2 | **1.49 (1.14 to 1.96)** | **0.004** | **0.010** | 0.98 (0.70 to 1.38) | 0.926 | 0.926 |
| GDF15 | **1.83 (1.34 to 2.48)** | **1.25E-04** | **5.44E-04** | **1.62 (1.16 to 2.25)** | **0.004** | **0.041** |
| GM2A | **1.36 (1.06 to 1.74)** | **0.017** | **0.026** | 1.21 (0.87 to 1.68) | 0.267 | 0.385 |
| HAVCR2 | **1.79 (1.31 to 2.44)** | **2.28E-04** | **8.88E-04** | 0.98 (0.69 to 1.40) | 0.917 | 0.926 |
| IGFBP2 | **1.43 (1.01 to 2.02)** | **0.041** | **0.049** | 1.19 (0.79 to 1.77) | 0.406 | 0.545 |
| IGFBP4 | **2.06 (1.48 to 2.87)** | **1.91E-05** | **2.39E-04** | 1.41 (0.92 to 2.16) | 0.114 | 0.170 |
| IGFBPL1 | **1.41 (1.07 to 1.86)** | **0.016** | **0.026** | 1.37 (0.98 to 1.93) | 0.064 | 0.140 |
| JAM2 | **1.50 (1.07 to 2.09)** | **0.017** | **0.026** | 1.61 (1.10 to 2.37) | 0.014 | 0.062 |
| KRT19 | **1.71 (1.31 to 2.25)** | **9.62E-05** | **4.69E-04** | 1.36 (0.98 to 1.91) | 0.069 | 0.140 |
| LAYN | **1.99 (1.42 to 2.80)** | **7.62E-05** | **4.25E-04** | **1.81 (1.22 to 2.68)** | **0.003** | **0.041** |
| LY6D | 1.27 (0.93 to 1.73) | 0.129 | 0.144 | 1.11 (0.76 to 1.61) | 0.604 | 0.693 |
| NCR3LG1 | 1.31 (0.96 to 1.79) | 0.084 | 0.097 | 1.03 (0.73 to 1.47) | 0.865 | 0.912 |
| NPC2 | **1.60 (1.20 to 2.14)** | **0.002** | **0.005** | 1.39 (0.99 to 1.96) | 0.058 | 0.140 |
| OCLN | **1.76 (1.38 to 2.24)** | **4.94E-06** | **9.64E-05** | **1.47 (1.11 to 1.96)** | **0.008** | **0.049** |
| OGN | **1.61 (1.12 to 2.31)** | **0.009** | **0.020** | 1.54 (1.00 to 2.37) | 0.051 | 0.140 |
| PIGR | **1.46 (1.10 to 1.92)** | **0.008** | **0.017** | 1.47 (1.09 to 2.00) | 0.013 | 0.062 |
| PLAUR | **1.47 (1.07 to 2.00)** | **0.016** | **0.026** | 1.43 (1.02 to 2.01) | 0.040 | 0.129 |
| PSAP | **1.32 (1.01 to 1.72)** | **0.040** | **0.049** | 1.20 (0.86 to 1.66) | 0.285 | 0.397 |
| REG4 | **1.53 (1.16 to 2.01)** | **0.002** | **0.006** | **1.77 (1.26 to 2.47)** | **9.04E-04** | **0.018** |
| SHISA5 | **1.67 (1.23 to 2.28)** | **0.001** | **0.004** | 1.44 (0.98 to 2.11) | 0.064 | 0.140 |
| TFF3 | **1.42 (1.13 to 1.79)** | **0.003** | **0.007** | 1.12 (0.79 to 1.59) | 0.515 | 0.608 |
| TFPI2 | 1.03 (0.76 to 1.40) | 0.836 | 0.836 | 1.32 (0.97 to 1.79) | 0.073 | 0.140 |
| TNFRSF10B | **1.96 (1.43 to 2.69)** | **3.06E-05** | **2.39E-04** | 1.56 (1.10 to 2.22) | 0.013 | 0.062 |
| TNFRSF12A | **1.49 (1.07 to 2.08)** | **0.017** | **0.026** | **2.30 (1.67 to 3.17)** | **3.14E-07** | **1.23E-05** |
| TNFRSF19 | **1.50 (1.05 to 2.16)** | **0.027** | **0.037** | 1.57 (1.01 to 2.43) | 0.043 | 0.129 |
| TNFRSF1B | **1.95 (1.50 to 2.55)** | **8.34E-07** | **3.25E-05** | 1.08 (0.75 to 1.57) | 0.674 | 0.751 |
| WFDC2 | **1.95 (1.43 to 2.66)** | **2.72E-05** | **2.39E-04** | 1.43 (0.96 to 2.11) | 0.077 | 0.140 |

Estimated using Cox proportional hazards, adjusting for study site, age, sex, race, ethnicity, estimated glomerular filtration rate, education, body mass index, systolic blood pressure, anti-hypertensive medication, dyslipidemia, diabetes, smoking, and heavy alcohol use. N=53 incident adjudicated clinical HFpEF events and N=40 incident HFrEF events occurred over a median [interquartile range] follow-up period of 9.8 [1.0] years. N=13 HFmEF cases observed but not analyzed due to inadequate statistical power, and N=12 incident heart failure cases excluded due to lack of ejection fraction data availability for subtyping. HFpEF=HF with left ventricular ejection fraction (LVEF) ≥50%; HFrEF=LVEF <40%; HFmEF=LVEF 40-49%.

HR=hazard ratio; SD=standard deviation; CI=confidence interval; FDR=false discovery rate (Benjamini-Hochberg).

**Bold** indicates statistically significant following multiple testing correction (FDR<0.05).

**SUPPLEMENTAL FIGURE S1.** Flow diagram of SMASH study participants included in analysis sample.

Selection SMASH Olink proteomics substudy included complete cardiovascular magnetic resonance (CMR) with late gadolinium enhancement for the purpose of additional analyses and cost effectiveness.

**SUPPLEMENTAL FIGURE S2.** Spearman’s correlation between plasma abundances of 39 proteins independently associated with both HIV seropositivity and elevated myocardial extracellular volume fraction, concordant directionality, among PWH and PWOH in the United States (*n*=342).

Also viewable as table (Supplemental Table S6) in ‘Supplemental Tables.xlsx’

**SUPPLEMENTAL FIGURE S3.** Unadjusted distribution of plasma protein cluster level by HIV serostatus and plasma HIV RNA in SMASH.

PWOH=people without HIV; PWH=people with HIV; undetectable HIV RNA=plasma RNA ≤50 copies/mL; detectable HIV RNA=plasma RNA >50 copies/mL.

Multivariable adjusted mean  difference in plasma cluster level by HIV serostatus was previously published in Peterson TE, Hahn VS, Moaddel R, *et al.* Proteomic signature of HIV-associated subclinical left atrial remodeling and incident heart failure. *Nat Commun* **16**, 610 (2025). [https://doi.org/10.1038/s41467-025-55911-0](https://doi.org/10.1038/s41467-025-55911-0)

Estimated using linear regression with robust variance, adjusting for age, sex, race, ethnicity, education level, estimated glomerular filtration rate, hepatitis C virus infection, current hazardous alcohol use, pack-years of smoking in prior 5 years, stimulant use in prior 5 years, and opioid use in prior 5 years.

Mean difference in plasma level comparing PWH to PWOH: 0.48 SD (95% CI: 0.28‒0.67; p=3.99E-06).

Comparing PWH with undetectable viral load (HIV plasma RNA ≤50 copies/mL) to PWOH: 0.42 SD (95% CI: 0.21‒0.64; p=1.02E-04).

**SUPPLEMENTAL FIGURE S4.** Associations between identified HIV-associated proteomic signature of myocardial fibrosis and clinical characteristics in SMASH (*n*=342)  

Standardized mean difference in plasma protein abundance among participants with vs. without dichotomous characteristic or per standard deviation increment in continuous characteristic, estimated using linear regression. **p*<0.05, ***p*<0.01, and ****p*<0.001.

LV=left ventricular; LGE=presence of late gadolinium enhancement; HCV=hepatitis C infection; ART=antiretroviral therapy; MEbrown=weighted average of protein cluster plasma abundance.

**SUPPLEMENTAL FIGURE S5.** Flow diagram of MESA study participants included in cross-sectional and longitudinal analysis samples.

Reflects MESA data availability as of January 2025. CMR=cardiovascular magnetic resonance imaging; Gd=gadolinium-enhanced; ECV=myocardial extracellular volume fraction; HF=heart failure.

**SUPPLEMENTAL FIGURE S6.** Associations between clinical characteristics and validated HIV-associated proteomic signature of myocardial fibrosis and incident heart failure in MESA (*n*=3223)

Standardized mean difference in plasma protein abundance among participants with vs. without dichotomous characteristic or per standard deviation increment in continuous characteristic, estimated using linear regression. **p*<0.05, ***p*<0.01, and ****p*<0.001.

LV=left ventricular; LGE=presence of late gadolinium enhancement; MEbrown=weighted average of protein cluster plasma abundance.
